# Bioinspired Zinc Macrocycles Confined in UiO‐67: A Heterogeneous Synzyme for Sustainable CO_2_ Valorization

**DOI:** 10.1002/cssc.71010

**Published:** 2026-08-27

**Authors:** J. E. Chiva, J. E. Sánchez‐Velandia, F. Esteve, F. G. Cirujano, C. Vicent, N. Martín, B. Altava, E. García‐Verdugo

**Affiliations:** ^1^ Supramolecular and Sustainable Research Group Inorganic and Organic Department Universitat Jaume I Castelló de la Plana Spain; ^2^ Laboratoire de Chemie Supramoléculaire Institut de Science et d’ingénierie supramolécularies (ISIS) Supramolecular and Sustainable Research Group Inorganic and Organic université de Strasbourg Strasbourg France; ^3^ SCIC Universitat Jaume I Castelló de la Plana Spain

**Keywords:** CO_2_ activation, cyclic carbonate, macrocycle, metal organic framework, synzyme

## Abstract

Nature relies on multifunctional microenvironments to combine substrate recognition, activation, and catalysis with remarkable efficiency. Inspired by this principle, we report heterogeneous synzymes generated by the supramolecular immobilization of pseudopeptidic zinc macrocyclic complexes onto UiO‐67 for CO_2_ activation and valorization. The zinc chiral pseudopeptidic macrocycles can be selectively prepared through solvent‐directed macrocyclization, exploiting the conformational preorganization of the precursors to create enzyme‐inspired catalytic motifs. Comprehensive structural and computational studies of hybrid materials reveal a defect‐driven encapsulation mechanism. The bulky macrocycles partially perturb the UiO‐67 framework and reduce porosity, but also markedly improve the thermal stability of the hybrid materials. Despite this porosity loss, the hybrids display enhanced catalytic activity in the additive‐free cycloaddition of CO_2_ to epoxides at ambient pressure. C3@UiO‐67 outperforms both the pristine MOF and the molecular zinc complex, revealing a cooperative microenvironment where hydrogen‐bond‐stabilized “naked” iodides promote epoxide ring opening and secondary amines assist CO_2_ activation. Overall, this work shows that looking to nature for catalytic design principles can deliver bioinspired MOF–macrocycle synzymes as robust platforms for mild and sustainable CO_2_ coactivation and valorization.

## Graphical abstract

By mimicking nature’s use of weak supramolecular interactions, zinc macrocycles are assembled within defective UiO‐67 to create cooperative synzymes for CO_2_ valorization. Confined amines, hydrogen‐bonding networks and accessible iodides work in concert to activate CO_2_ and epoxides at ambient pressure, delivering additive‐free catalysis that outperforms the isolated MOF and molecular complex.

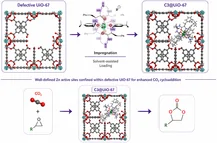

## 1 Introduction

The catalytic conversion of carbon dioxide into value‐added products remains one of the central challenges in sustainable chemistry. As an abundant, inexpensive, nontoxic, and renewable C1 feedstock, CO_2_ is an attractive carbon source [1]. However, its high thermodynamic stability and kinetic inertness demand the development of highly efficient catalytic systems capable of promoting its activation under mild conditions [2]. Among the different CO_2_‐based transformations, the cycloaddition of CO_2_ to epoxides is particularly appealing because it provides cyclic carbonates with 100% atom economy, often under solvent‐free conditions [3]. These products are relevant intermediates and solvents in fine chemistry, polymer synthesis, electrolytes, and other industrial applications [4, 5, 6].

**FIGURE 1:**
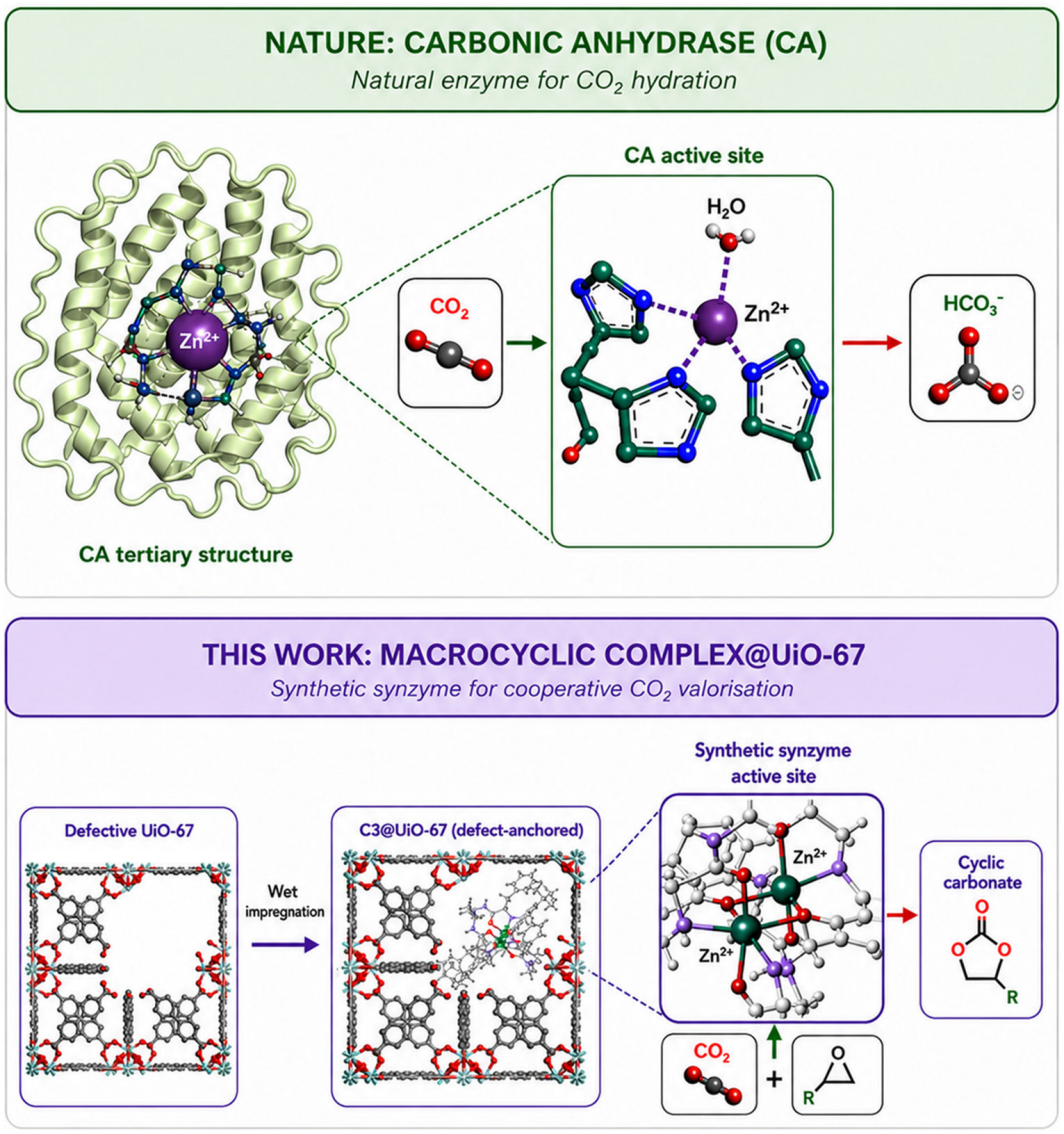
Bioinspired heterogenization strategy for cooperative CO_2_ valorization. A pseudopeptidic Zn(II) macrocycle was immobilized within UiO‐67 MOF to mimic enzymatic cooperative effects in a confined microenvironment for the cycloaddition of CO_2_ to epoxides.

State‐of‐the‐art catalytic approaches for CO_2_ activation and conversion commonly rely on Lewis acidic or Lewis basic sites in combination with nucleophilic co‐catalysts. Although this strategy has afforded highly active catalytic systems, it often requires external additives, elevated temperatures, high pressures, or structurally complex catalyst designs [7, 8]. In this context, enzymatic catalysis has emerged as a powerful source of inspiration for the development of more efficient and selective catalytic platforms [9, 10]. Carbonic anhydrase (CA), a highly efficient zinc‐dependent metalloenzyme, is particularly relevant in this regard owing to its ability to rapidly and reversibly activate CO_2_ under mild conditions (Figure 1) [11]. However, the direct implementation of native CA in industrial processes remains limited by its high cost, modest operational stability, and sensitivity to changes in temperature and pH [12].

These limitations have motivated extensive efforts to translate the key structural and functional features of the CA active site into robust synthetic catalysts. In CA, a Zn(II) center operates cooperatively with the surrounding amino acid residues, creating a highly organized microenvironment for substrate binding and activation. Reproducing this cooperative architecture has led to the development of multifunctional catalytic systems capable of synergistic CO_2_ activation [13, ‍^14^]. Representative examples include small‐molecule metal complexes, which offer high structural tunability [15]; supramolecular frameworks, which can provide enhanced thermal stability [16]; and nanomaterials, which introduce confined and porous microenvironments conducive to cooperative catalysis [17].

Building upon this biomimetic approach, we recently demonstrated that the use of highly preorganized pseudopeptidic Zn(II) macrocycles can promote exceptionally high conversions under homogeneous conditions. These systems behave as structural analogues of the CA‐active site, reproducing key aspects of its cooperative mode of action and enzyme‐like supramolecular interactions [18]. Traditionally, the immobilization of homogeneous catalysts onto solid supports has been pursued primarily to facilitate catalyst recovery and reuse. However, the use of metal–organic frameworks (MOFs) as porous platforms offer a more sophisticated opportunity as the support may actively contribute to catalysis rather than acting merely as an inert scaffold [19].

In this regard, the integration of well‐defined molecular active sites within a confined, CO_2_‐philic crystalline matrix can generate catalytic microenvironments capable of modulating the reactivity of the overall system [20, 21]. MOF‐based composites have been widely investigated for the cycloaddition of CO_2_ with epoxides [22, 23, 24]; however, pristine MOFs often suffer from limited stability, modest product yields, and the need for relatively harsh reaction conditions to achieve efficient conversion. These limitations can be mitigated through the incorporation of transition‐metal sites or additional functional components into the framework [25, 26, 27, 28, 29, 30], which may not only combine the individual properties of each component but also promote synergistic interactions that enhance the catalytic performance.

To address these challenges and further advance the concept of enzyme‐inspired catalysis, we have designed a new multifunctional amino‐functionalized pseudopeptidic Zn(II) macrocycle (Figure 1). In contrast to our previously reported structural analogue, this new catalyst incorporates multiple active elements, combining Lewis acidic Zn(II) centers with a central amine functionality specifically introduced to promote CO_2_ capture and preorganization. By embedding this carbonic‐anhydrase‐inspired macrocycle into UiO‐67 through a straightforward postsynthetic wet‐impregnation approach, we generate a cooperative host–guest catalytic system. Within the MOF pores, the framework provides a protective and CO_2_‐enriched microenvironment, while the central amine acts as a proximal binding site that concentrates and directs CO_2_ toward the Zn(II) catalytic centers [31]. This spatially organized arrangement is expected to enhance cooperative substrate activation and, consequently, improve the overall catalytic activity of the hybrid material.

In this work, we evaluate the catalytic performance of this newly developed heterogenized system and compare it with that of its previously reported structural analogue lacking the central amine. Rather than considering heterogenization as a merely practical strategy for transferring a homogeneous catalyst onto a solid matrix, this study focuses on how targeted modifications in the molecular structure of the catalyst, specifically, the incorporation of a central amine group, influence catalyst–support interactions and modulate the catalytic behavior of the resulting hybrid material. In particular, we investigate the role of a proximal amine‐based CO_2_‐binding site in promoting cooperative CO_2_ activation within the confined pores of UiO‐67. By establishing a structure–function relationship between the molecular catalyst, the MOF host, and the catalytic response, this work aims to demonstrate that the rational incorporation of bioinspired mimicking catalysts into porous crystalline matrices can generate synergistic effects across the molecular and material scales, thereby offering a powerful platform for enhancing CO_2_ conversion under milder and more efficient conditions.

## 2 Results and Discussion

### 2.1 Synthesis and Characterization of the Pseudopeptidic Macrocycles Catalyst

**FIGURE 2:**
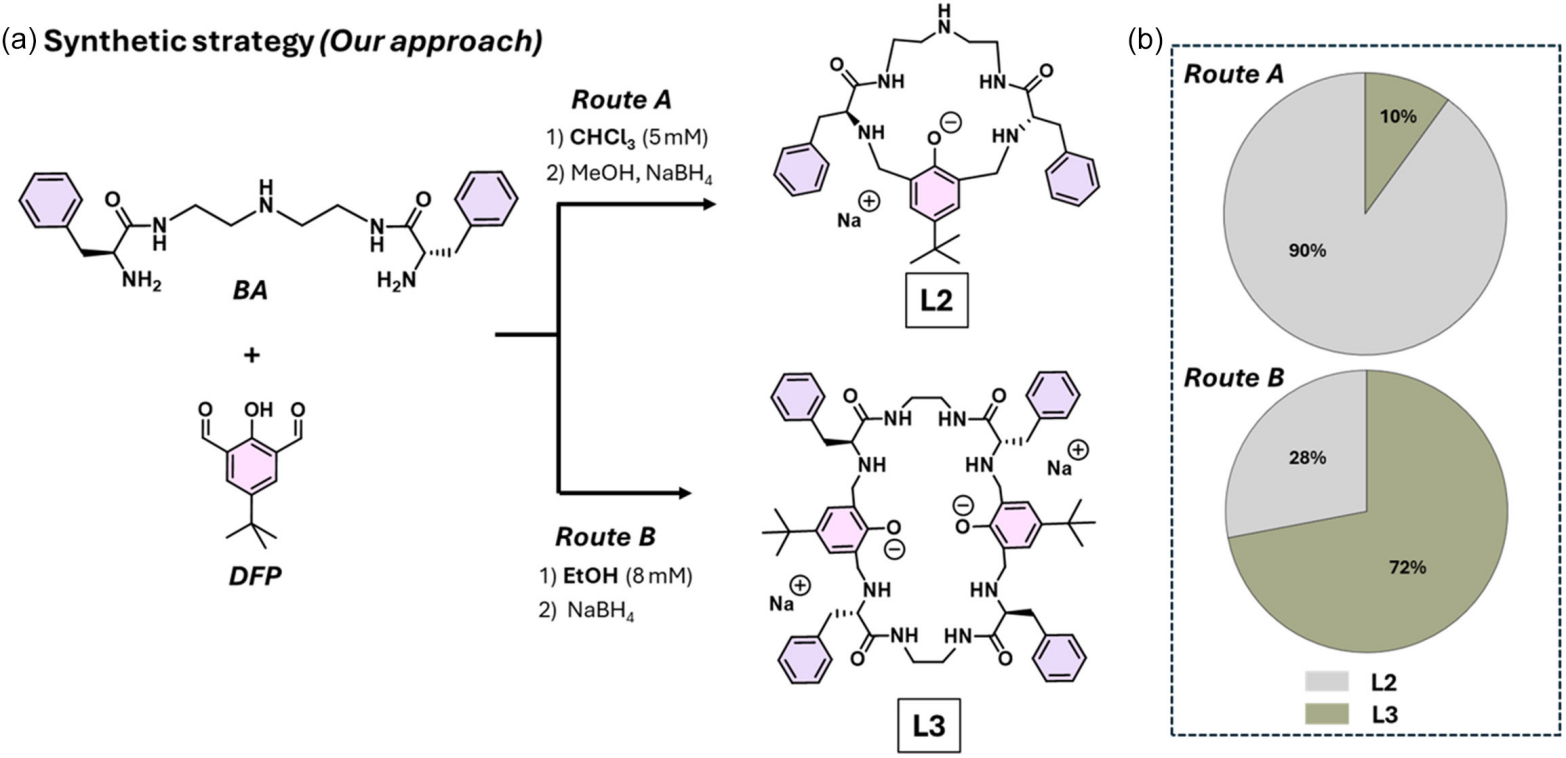
(a) General synthetic routes of macrocycles **L2** and **L3.** See the Supporting Information for synthetic and characterization details of the new pseudopeptidic macrocyclic ligands and (b) **L2/L3** molar ratio selectivity.

The synthesis of the family of macrocyclic compounds was carried out following the procedure previously described for the analogous macrocycle **L1** (see the structure in Figure S1) [18], via the imine condensation of the open‐chain C2‐pseudopeptidic bisaminoamide (**BA**) with 5‐(*tert*‐butyl)‐2‐hydroxyisophthalaldehyde (**DFP**) in CHCl_3,_ followed by in situ imine reduction (Figure 2, Route A). Under these conditions, the [1 + 1] macrocycle (**L2**) was obtained as the major product, alongside a minor amount of the [2 + 2] macrocycle (**L3**) and oligomeric byproducts (90% vs. 10% for **L2** and **L3**, respectively, Figure 2 Route A and Figure S2a). The ^1^H NMR characterization unveiled a duplicated set of signals for the [1 + 1] product **L2,** which were assigned to two distinct [1 + 1] macrocyclic conformers (denoted as **L2A** and **L2B**) that could be isolated by flash chromatography, being one of them dominant (molar ratio **L2A**/**L2B** 9/1). It is well established that solvent polarity influence critically the macrocyclization reaction outcome of such pseudopeptidic systems [32]. Formation of **L2** was highly favored in CHCl_3_ due to the intrinsic precursor U‐turn preorganization (sustained by H‐bonding from the amide and amine groups), rendering the [1 + 1] architecture as the thermodynamically favored product [33, 34].

**FIGURE 3:**
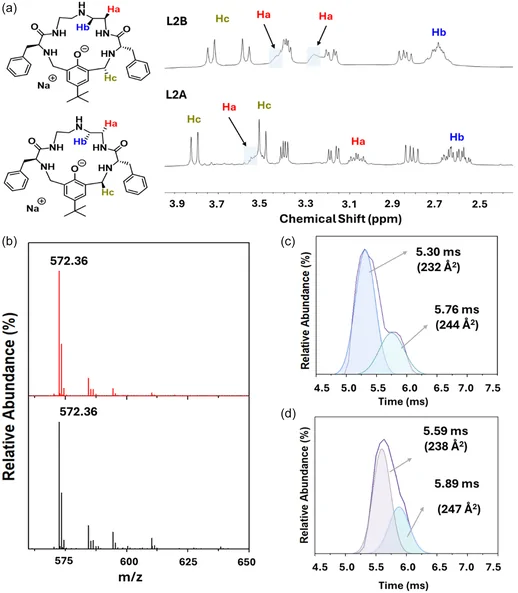
(a) Partial ^1^H NMR spectra (400 MHz, CDCl_3_, 25º C) of **L2A** and **L2B** conformers of the [1 + 1] macrocycle. (b) Positive ESI mass spectra of isolated conformers **L2A** (bottom) and **L2B** (top) in methanol. (c) and (d) Ion mobilograms of the species at *m*/*z* 572 of conformers **L2B** (c) and **L2A** (d); peak annotations are the CCS values (in Å^2^) derived from the observed drift times (see the Supporting Information for details).

Notably, the most significant variation in the ^1^H NMR between both conformers was observed in the chemical shift separation of the diastereotopic protons of the ethylene bridge moieties, which point to a restricted C─C rotation around such bond (Figure 3a). Conformer **L2A** exhibits a larger chemical shift difference between these signals, suggesting a more conformationally constrained structure compared to **L2B** in an aprotic nonpolar solvent such as CDCl_3_. Surprisingly, no interconversion between these two conformers was observed in CD_3_OD even when heated at 60 °C or subjected to acidic conditions (see Figure S3). Such conformers are ubiquitous in analogous macrocycles featuring phenyl or naphtanyl bridges and are associated with a dynamic flipping of the aromatic bridging group (free C─C rotation) with respect to the macrocyclic main plane as well as to transannular interactions such as hydrogen bonds and some other hydrophobic effects [35, 36, 37, 38, 39]. The successful isolation of both conformers could arise from the restricted rotation about such benzylic C─C bond (see Figure 3) caused by the presence of the bulky *tert*‐butyl group being oriented either outward or inward from the cavity of the **L2** macrocycle.

Both conformers exhibited the expected [M + 2H − Na]^+^ pseudo‐molecular peak at *m/z* 572.3 as the base peak (Figure 3b). Ion mobility‐mass spectrometry MS (IM‐MS) studies were carried out to gain insights on the shape and size of both conformers that proved to be crucial to unambiguously assign each specific conformer (see the Supporting Information for details) [39, 40, 41]. The mobility trace of the [M+2H − Na]^+^ peak at *m/z* 572.3 from conformer **L2A** (see Figure 3c) revealed a mobility peak associated to a species with a CCS (collisional cross section) of 238 Å^2^ (and a barely observed shoulder of 247 Å^2^), whereas the same species at *m/z* 572.3 from conformed **L2B** displayed a mobility trace associated to a species with a smaller CCS 232 Å^2^ (accompanied by a second mobility trace associated to a species of CCS 244 Å^2^) (see Figure 3d). Such a significant difference in CCS clearly indicates that conformer **L2A** adopts a more extended topology (associated to the bulky groups bulky *tert*‐butyl group being oriented outward from the cavity of the **L2** macrocycle) than its **L2B** congener [42].

When the cyclization reaction was performed in a protic solvent (EtOH), the [2 + 2] macrocycle **L3** was successfully obtained as the major product (72% vs 28% for **L3** and **L2**, respectively, Figures 2, Route B and S2b). This shift in product distribution is due to the protic solvent‐mediated disruption of intramolecular H─bond interaction in the bis(amino amide) precursor, thus disrupting the characteristic U‐turn conformation and producing a more extended conformation [43]. This different conformational behavior in apolar versus protic solvents was also evidenced by routine ^1^H NMR characterization of the bis(amino amide) precursor. For example, in CDCl_3,_ the chemical shift difference of the benzylic protons decreases from Δ*δ* = 0.51 ppm to Δ*δ* = 0.13 ppm in CD_3_OD, consistent with a more rigid conformation due to the formation of intramolecular hydrogen bonding interactions (Figure ‍S4). Calculated equilibrium conformations (GFN‐xTB/CREST) of the intermediate reaction in chloroform and methanol were also performed (Figure S5). The calculations reveal a solvent‐induced conformational change, in chloroform, where the unreacted amine and aldehyde groups are brought into close proximity (7.5 Å), preorganizing the system for the 1 + 1 intramolecular macrocyclization. In contrast, the larger distance (12.5 Å) observed in methanol hinders the direct ring closure, thereby promoting the formation of the 2 + 2 macrocycle. Overall, these findings emphasize how solvent‐induced changes in the conformational preorganization of the precursor can be exploited to control the outcome of macrocyclization processes governed by dynamic covalent chemistry [44].

**FIGURE 4:**
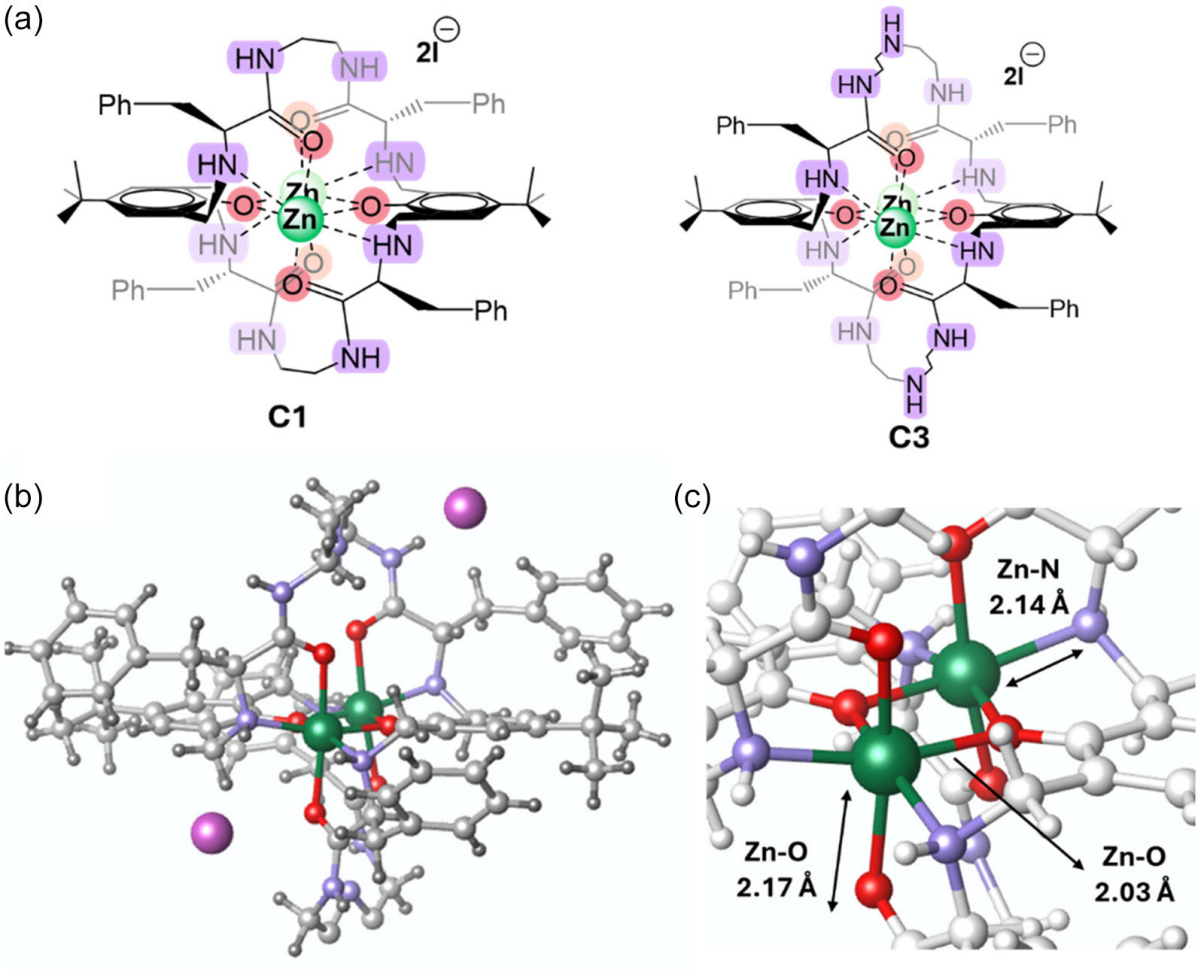
(a) Bimetallic Zn complexes **C1** and **C3** studied in this work. See the Supporting Information for synthetic and characterization details of the new bimetallic complex **C3**. (b) and (c) Solid‐state structure for **C3**. Iodide anions are highlighted in violet. Distances between atoms and metallic cores are highlighted.

For the synthesis of the corresponding metal complexes, the sodium salts of macrocycles **L2** ([1 + 1] system) and **L3** ([2 + 2] system) were reacted with ZnI_2_ as described previously for the synthesis of the bimetallic complex **C1** (Figure 4) [18]. Metal coordination to the smaller macrocycle [1 + 1]**L2** was studied by NMR and MS methods. A significant broadening of the signals in the ^1^H NMR spectrum (Figure S6b) was observed, which is indicative of aggregation processes in solution, as commonly observed in metalo‐supramolecular systems forming polymeric assemblies [45]. The FT‐IR confirmed the formation of the supramolecular metal polymeric system by shifting to a low wavenumber of the CO vibrational bands. Furthermore, ESI‐MS analysis corroborated the lower stability, as different peaks associated to the free macrocycle **L2** were observed at *m*/*z* 286.69 and 572.37 for [**L2**+3H‐2Na]^2+^ and [**L2** + 2H‐2Na]^+^ species, respectively, while a minor peak corresponding to the [**L2 **− 2Na + Zn]^+^ species at *m*/*z* 636.28 was detected (Figure S6b).

In the case of **L3**, the expected bimetallic pseudopeptidic complex **C3** could be isolated in an 85% yield (Figure 4a). The formation of this bimetallic complex was confirmed by ^1^H NMR spectroscopy. MS and IM‐MS characterization (Figure S6a) confirm the formation of a single product which display prominent peaks associated to the [L3Zn_2_ − 2Na]^2+^ adduct at *m*/*z* 635.28, the [L3Zn_2_ + H − 2Na]^3+^ adduct at *m*/*z* 424.52, and the [L3Zn_2_+2H − 2Na]^4+^ adduct at *m*/*z* 317.65, all of them displaying a single, narrow and Gaussian‐shaped mobility trace in its Ion mobility MS analysis.

The solid‐state structure of the bimetallic complex **C3** (Figure 4b) provided unequivocal evidence of its formation, revealing a geometry analogous to that previously reported for complex **C1** [18]. Notably, despite the increased functionalization of the macrocyclic framework, the overall coordination motif was preserved. As in the analogous system, the two phenoxy groups bridge the zinc centers, leading to an intermetallic Zn─Zn distance of 3.10 Å. Each metal center adopts a distorted octahedral geometry, in which the equatorial plane is defined by the amino donors of the ligand, while the amide oxygen atoms occupy the axial positions, with Zn–ligand bond distances in the narrow range of 2.13–2.18 Å. Importantly, the central secondary amine remains noncoordinating, indicating that its incorporation does not perturb the primary coordination environment. Consistent with the behavior observed for the **C1** analogue, the iodide counterions do not directly coordinate with the zinc centers. Instead, they are stabilized through hydrogen‐bonding interactions with the NH groups of the macrocycle. Although a slight asymmetry is observed in their spatial disposition (Zn···I distance of 4.52 and 7.68 Å), the anions remain effectively isolated within the supramolecular framework. This arrangement supports the preservation of their “naked” character and, consequently, their high nucleophilicity.

### 2.2 Synthesis and Characterization of the Macrocycle‐MOF Catalytic Systems

**FIGURE 5:**
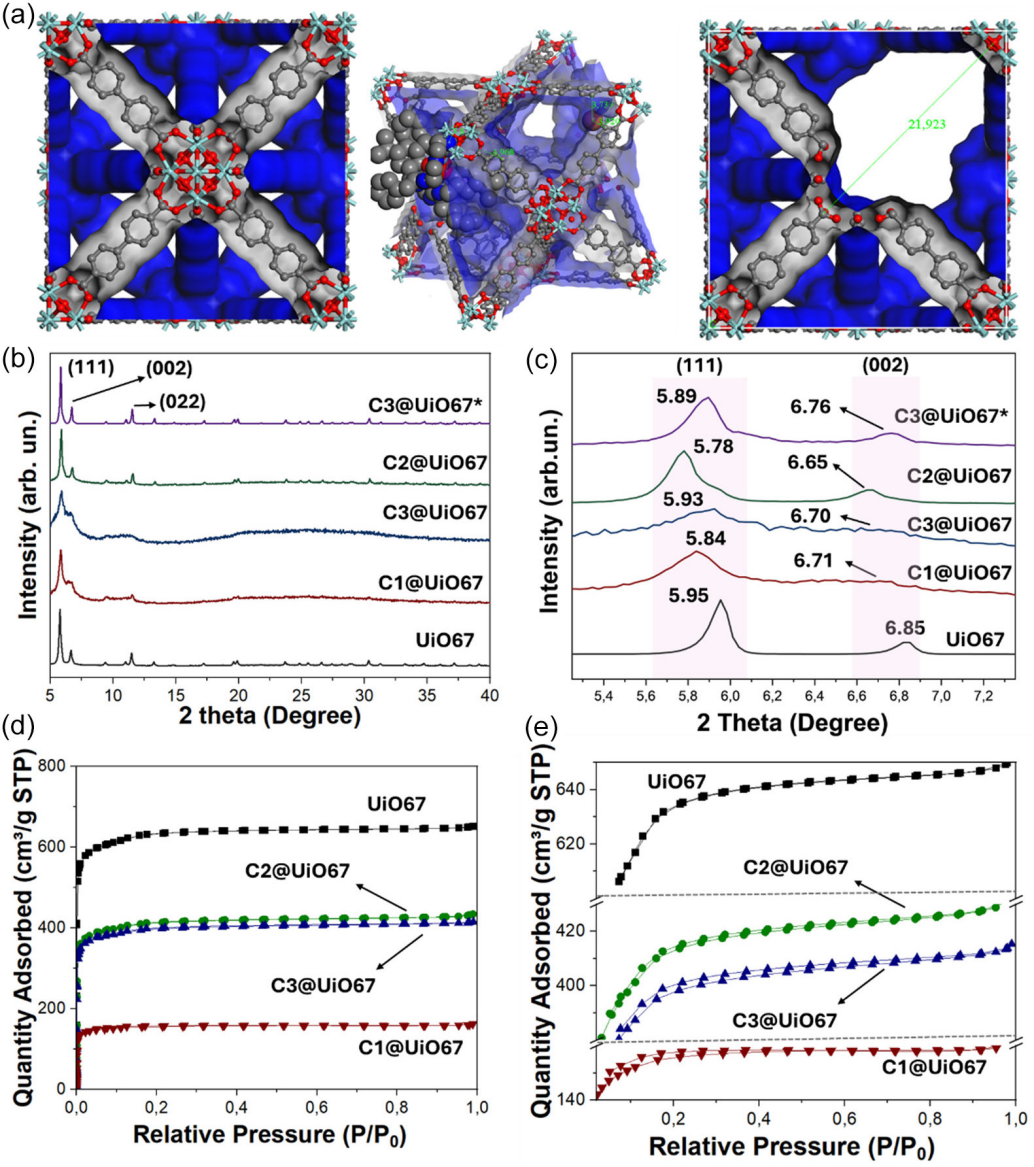
(a) Molecular Mechanics modeling of an ideal **UiO‐67** lattice and the proposed defective framework, highlighting the generation of missing linkers and metal nodes showing the expanded internal free volume that successfully accommodates the bulky guest complex; (b) wide‐angle XRD pattern; (c) 5°–8° (2*θ*) extended XRD pattern; (d) and (e) N_2_ adsorption–desorption isotherms at 77 K highlighting the hysteresis loop attributed to defect‐induced mesoporosity.

The wet impregnation method was used to modify the structure of UiO‐67 with macrocyclic supramolecular catalysts [46]. To further understand the immobilization mechanism at the molecular level, structural models of the MOF–macrocycle system were evaluated (Figure 5a). Molecular mechanics allow comparing an ideal **UiO‐67** lattice with a defective one, revealing that the pristine cavities are insufficiently sized to accommodate the bulky macrocyclic complexes (left part of Figure 5a). Instead, the simulations suggest that their successful encapsulation relies on the generation of framework defects, specifically missing linkers and metal nodes, which expand the internal free volume (the middle and right parts of Figure 5a). This defect‐driven immobilization aligns with the framework perturbation and loss of crystallinity previously observed in XRD analyses.

The **UiO‐67** structural and morphological modifications triggered by the macrocycle impregnation were evaluated by XRD, N_2_ adsorption–desorption, thermogravimetric analysis (TGA), scanning electron microscopy (SEM), and TEM. The isolated macrocyclic complexes **C1**, **C2**, and **C3** did not present specific XRD patterns when prepared under identical conditions prior to the impregnation step. Upon immobilization, the XRD analyses of the hybrid systems generally showed the expected pattern of pristine UiO67, with the most intense peaks corresponding to the characteristic [111,002] and [022] crystallographic planes (space group symmetry Fm‐3m, Figure 5b). Whereas the crystallinity of **UiO‐67** was not significantly affected by the incorporation of the smaller [1 + 1] macrocycle complex **C2**, its impregnation with the larger [2 + 2] compounds (**C1** and **C3**) via mechanical stirring led to a noticeable loss in crystallinity, consistent with the known susceptibility of MOFs to mechanically induced amorphization and structural degradation [47]. This is evidenced by the broadening and intensity drop of the peaks (**C1@UiO‐67** and **C3@UiO‐67**, Figure 5b), indicating a perturbation of the 3D arrangement, as well as alterations in lattice parameters and crystallite size distribution due to linker defects. A shift of the [111] and [002] planes to lower angles was observed upon incorporation of the macrocycles complexes into the UiO67 framework (Figure 5c). Interestingly, when the immobilization of **C3** was performed using milder orbital shaking instead of mechanical stirring (C3@UiO‐67*, the purple pattern in Figure 5b), the high crystallinity of the MOF was fully preserved. This suggests that the structural modification observed earlier is the combined result of the bulkier sizes of **C1** and **C3** and the harsh mechanical forces applied during conventional stirring.

Regarding the textural properties, N_2_ adsorption–desorption (Figure 5d) studies revealed a significant decrease in the surface area upon macrocycle immobilization, following the order: pristine UiO‐67 > C2@UiO‐67 = C3@UiO‐67 > C1@UiO‐67. In all cases, the profiles exhibited Type I isotherms, which are in good agreement with the preservation of the microporous nature of the frameworks (Figure 5d). The average pore volumes followed a similar downward trend, with values of 0.95, 0.62, 0.60, and 0.24 cm^3^/g for UiO‐67, C2@UiO‐67, C3@UiO‐67, and C1@UiO‐‍67, respectively (Table S1). Notably, both the C2@UiO‐67 and C3@UiO‐67 systems retain approximately 62% of their original microporous area and around 68% of their external surface area; this proportional reduction supports a partial pore‐ and surface‐blockage model rather than total internal confinement.

The parent UiO‐67 exhibits a typical microporous adsorption behavior, characterized by a type‐I isotherm without any noticeable hysteresis loop, consistent with its highly ordered and defect‐poor framework (Figure 5e). However, upon incorporation of a macrocyclic species within the cavities of the UiO MOF structure, a deviation from this ideal behavior is observed. The presence of the bulky macrocycle imposes steric constraints during framework formation or postsynthetic modification, promoting the generation of structural defects such as missing linkers or missing metal clusters. These defects disrupt the long‐range periodicity of the lattice and locally expand the pore environment, giving rise to larger cavities within the mesoporous regime. As a result, the adsorption–desorption isotherm displays a hysteresis loop, indicative of capillary condensation in these defect‐generated mesopores, in contrast to the absence of hysteresis in pristine UiO‐67. This effect is more pronounced in the C1‐ and C3‐containing materials, which exhibit a lower degree of crystallinity, as evidenced by the attenuation and broadening of characteristic XRD reflections (see Figure 5b,c). The increased structural disorder in these samples points to a higher defect density, which in turn enhances mesopore formation, thereby explaining the more significant hysteresis observed and reinforcing the correlation between reduced crystallinity and defect‐induced mesoporosity in the modified framework.

**FIGURE 6:**
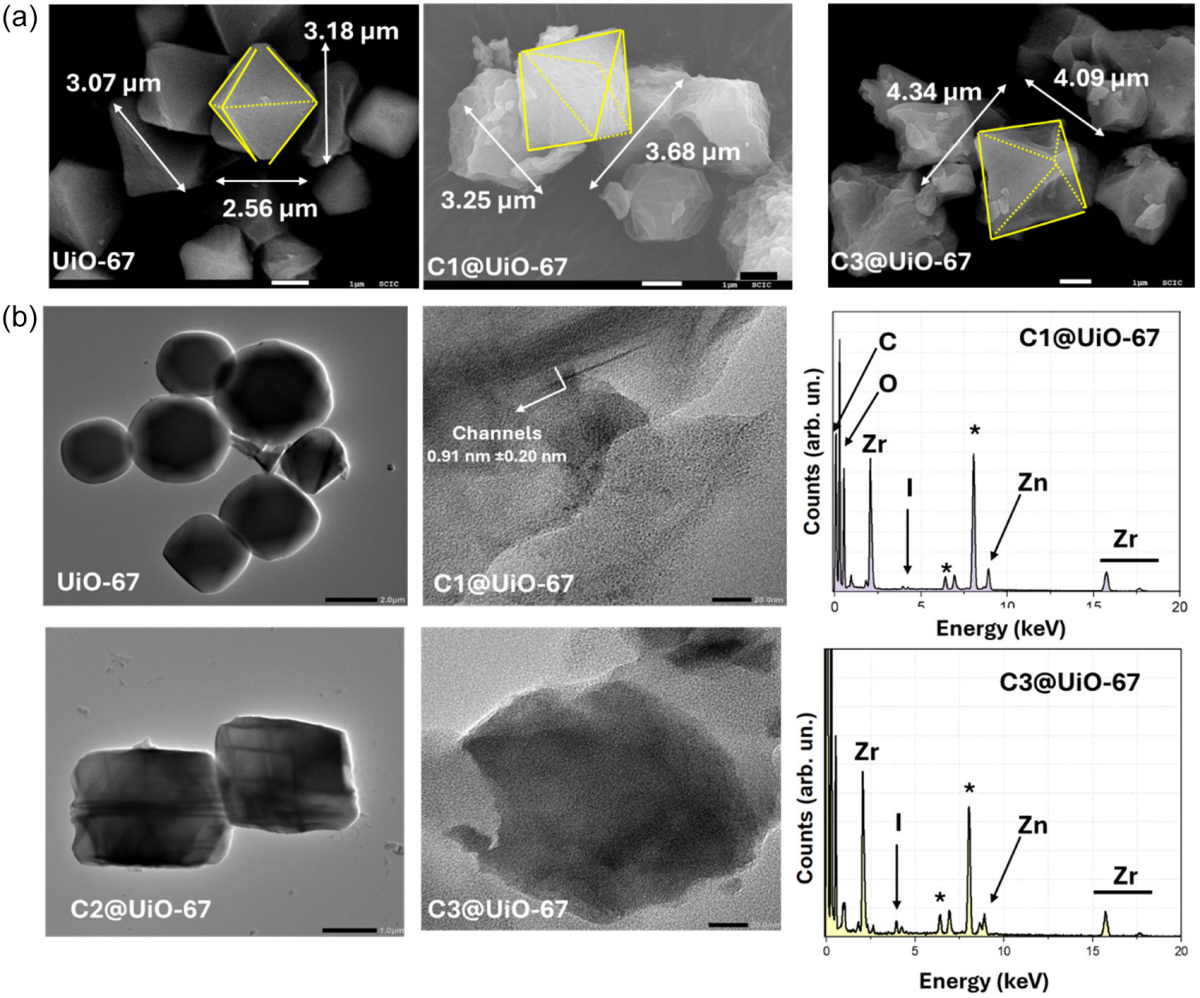
(a) SEM images of UiO‐67, C1@UiO‐67 and C3@UiO‐67. (b) TEM images and EDX spectra of the corresponding UiO‐67 and hybrid materials showing the elemental composition. In the EDX spectra, the asterisk indicates the presence of either Fe or Cu which are present in the sample holder during the TEM analysis.

Furthermore, SEM was used to study the morphological changes and component distribution at the MOF–macrocycle interphase (Figure 6a). As expected, well‐defined ortho‐octahedral crystals were observed for pristine UiO‐67. However, SEM analysis of the impregnated samples showed a higher level of perturbation and roughness on the surface of the MOF crystals. Measurements of the octahedral crystals indicated a substantial size difference before and after impregnation with the macrocyclic compounds. The postmodified UiO‐67 exhibited a significant increase in both dimensions and shape, suggesting lattice expansion and a loss of crystallinity. Such morphological modifications corroborate the deposition of the macrocycles and explain the significant decreases in the surface area and crystallinity observed across the hybrid samples. Furthermore, localized TEM‐EDX point analysis for C1@UiO‐67 and C3@UiO‐67 was performed on a representative hybrid crystal, confirming the presence of Zn and I, which were completely absent in the pristine MOF. These localized signals corroborate the successful incorporation of the macrocyclic complexes within the framework (Figures 6b and S8 for pristine UiO‐67).

TGA was performed to evaluate the thermal stability of the materials (Figures S9 for TGA and S8 for DTG). Following an initial minor mass loss, typically ascribed to the desorption of solvent molecules or uncoordinated species, pristine UiO‐67 showed a sharp mass loss centered at 555 °C (Figure S10, black line), corresponding to the decomposition of its organic linkers. In contrast, the hybrid systems exhibited a two‐step major decomposition profile. The first significant mass loss, occurring in the 500–530 °C range, is associated with the degradation of the macrocycles, while the second step at ~550 °C corresponds to the support breakdown. Notably, the isolated C1–C3 macrocyclic complexes undergo thermal degradation at much lower temperatures (between 330 and 450 °C). The pronounced increase in their decomposition temperature within the hybrid materials clearly proves their successful immobilization and thermal stabilization by the MOF framework, as confinement within porous hosts and host–guest interactions are known to restrict molecular mobility and improve resistance to thermal degradation [48].

Furthermore, the final inorganic residue of ca. 56% observed for pristine UiO‐67 decreases to approximately 50% in the hybrid systems. This reduction in the relative mass of inorganic species is consistent with the successful incorporation of the bulky, organic‐rich macrocyclic complexes, which increases the overall organic fraction of the final materials.

To assess the CO_2_ uptake capacity of the hybrid catalytic systems, adsorption isotherms were measured and compared with those of pristine **UiO‐67** (Figure S11). Anchoring the macrocyclic complexes onto the MOF leads to a decrease in CO_2_ adsorption capacity, likely due to partial pore blockage or the occupation of internal cavities. However, this reduction is notably more drastic for the **C1@UiO‐67** system (from ca. 3.3–1.34 mmol·g^−1^, see Table S1). This distinct behavior can be attributed to the nature of the complexed ligands, whereas the **C2** and **C3** complexes feature a free central secondary amine that provides intrinsic chemical affinity for CO_2_ capture (with a similar uptake of c.a. 2.3 mmol·g^−1^, see Table S1). Indeed, while the porosity (i.e., BET surface area and micropore volume) decreases up to 38% upon the incorporation of C3 within the framework, the CO_2_ adsorption capacity only decreases by 28%. Thus, the drastic capacity loss in **C1@UiO‐67** can be associated to the synergistic result of both physical steric hindrance (pore blockage), despite the similar CO_2_–philic nature of the parent and macrocíclic‐loaded MOF (with CO_2_ absorption energies of ca. 18 kJ·mol^−1^, using the Dubinin–Radushkevich method in Table S1). Therefore, despite the higher CO_2_ uptake observed for the macrocycle‐loaded UiO‐67, the CO_2_ adsorption energies remain essentially unchanged (~18 kJ·mol^−1^), indicating that the intrinsic CO_2_–framework interactions are comparable to those of the parent material within the UiO MOF system, confirming the double role of MOF as support and CO_2_ adsorbent. This suggests that the increased uptake is not driven by enhanced CO_2_ affinity but rather by structural modifications—namely, defect‐induced mesoporosity—that improve pore accessibility and facilitate diffusion and adsorption of CO_2_ molecules within the framework.

The interaction of **C3** with CO_2_ was investigated by ^1^H NMR spectroscopy. Upon exposure to CO_2_ at 5 bar, the resonances of the CH_2_ groups adjacent to the lateral amine groups shifted upfield, whereas those of the CH_2_ groups adjacent to the central secondary amine shifted downfield (Figure S15). Downfield shifts were also observed for the aromatic proton located close to the phenolic group and for the amide NH resonances. Upon depressurization, these signals shifted back to their original positions, supporting a partially reversible interaction between CO_2_ and the macrocyclic complex, accompanied by conformational changes in its coordination environment.

### 2.3 Catalytic Activity

**FIGURE 7:**
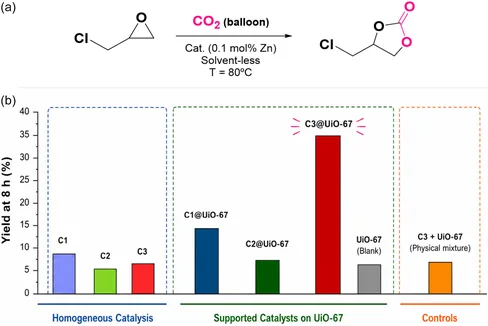
(a) Cycloaddition of CO_2_ to epichlorohydrin considered as the benchmark reaction in this work. (b) Catalytic evaluation for the different systems. Conversions determined after 8 h of reaction with selectivities >99%. Reaction conditions: 0.1 mol% Zn, epichlorohydrin (12.8 mmol), CO_2_ (balloon), solvent‐less, 80 °C.

With the hybrid materials fully characterized, we evaluated their catalytic performance in the cycloaddition of CO_2_ to epichlorohydrin under mild conditions (80 °C, 1 atm CO_2_ balloon; Figure 7a). Control experiments using the homogeneous macrocycles, under the tested conditions of 0.1 mol% Zn, revealed negligible activity for **C1, C2,** and **C3** (ca. 5%–8% yield) after 8 h, Figure ‍7b. Similarly, the pristine **UiO‐67** support displayed very poor background activity (< 7% after 8h, using 7.85 mg of UiO‐67/mmol epoxide). When evaluating the immobilized systems using 0.1 mol% Zn (which correspond to ca. 7.85 mg of **Cx@UiO‐67/**mmol epoxide), after 8h of reaction, the hybrid **C2@UiO‐67** performed poorly, mirroring the low activity of the bare MOF (< 9%), while **C1@UiO‐67** showed only a yield of ca. 15%, which is the sum of its individual component activity (**UiO‐67** and **C1)**. In striking contrast, the **C3@UiO‐67** material triggered an exceptional catalytic boost, reaching a moderate yield of approximately 35% under identical conditions (Figure 7b). This remarkable jump in activity highlights a profound cooperative effect [49, 50], and the performance of **C3@UiO‐67** vastly exceeds the sum of its individual components (homogeneous **C3** + bare **UiO‐67**), an enhancement that is completely absent in the analogous hybrid **C1‐UiO‐67** system.

In situ FTIR monitoring of the reaction kinetics (Figure S12) after 8h of reaction exhibited a linear dependence of product yield over time (Figure S14). Remarkably, the multifunctional **C3@UiO‐67** catalyst achieved a yield of 90% after 24 h (TON value 883 and TOF value 37 h^−1^, Table S2), an outstanding result given the mild temperature and low CO_2_ pressure employed, while its homologous **C1@UiO‐67** achieved an 82% conversion after 24h (TON value 805 and TOF value 33 h^−1^) and the pristine **UiO‐67 achieved** a lower yield of 71% (Table S2). We attribute this extraordinary synergistic performance to a cooperative host–guest interplay. On the one hand, the specific structural features of **C3**, particularly the presence of additional basic sites such as the central secondary amine, likely facilitate CO_2_ activation via transient carbamate formation. On the other hand, the microporous **UiO‐67** matrix acts as a highly effective CO_2_ adsorbent, drastically increasing the local gas concentration around the confined active sites. This dual activation pathway, uniquely enabled by the efficient confinement of **C3** in the MOF, perfectly rationalizes the massive gap in activity between the homogeneous **C3** catalyst and the heterogeneous **C3@UiO‐67** composite.

To prove the importance of cooperativity within the hybrid material, a control experiment was performed by physically mixing pristine **UiO‐67** (90 mg) and **C3** (10 mg, 0.007 mmol). In this unanchored system, direct interactions between the different species involved in the reaction are less favored. Under identical reaction conditions, the yield of the desired carbonate dropped to 7.5% (Figure 7b, the orange bar). Indeed, this conversion is even lower than the sum of the activities exhibited by **UiO‐67** and **C3** when tested individually (Table S2). This result confirms the relevance of the proposed heterogeneous synzymatic system, which ensures the close proximity of the species involved in the catalytic cycle.

As mentioned before, the presence of a clear hysteresis loop over the 0 < *P*/*P*_0_ < 1 range for the C3@UiO‐67 material can be associated with the emergence of defect‐induced mesoporosity within the UiO MOF (see Figure S7). The hysteresis indicates the formation of mesopores arising from structural defects such as missing linkers or clusters, which generate enlarged and more accessible pore domains compared to those of the purely microporous parent structure. These mesoporous features can facilitate the diffusion of reactant molecules, such as CO_2_ and epoxides, by reducing mass transport limitations within the framework. As a result, improved accessibility to active sites and enhanced molecular transport in the more defective structure likely contribute to the higher catalytic activity observed for the C3‐functionalized material. The induction period observed for C3@UiO‐67 can be rationalized by the time required to generate the fully active confined MOF–macrocycle microenvironment. During the early stage, diffusion of CO_2_ and epoxide into the UiO‐67 pores, substrate adsorption, and conformational reorganization of the confined macrocycle likely occur. After this activation stage, the hybrid system benefits from a cooperative mechanism in which the Zr nodes of UiO‐67 polarize the epoxide, the porous framework concentrates CO_2_, and the macrocycle stabilizes or assists the ring‐opening intermediate. This synergy explains why C3@UiO‐67 becomes significantly more active than either C3 or UiO‐67 alone.

**FIGURE 8:**
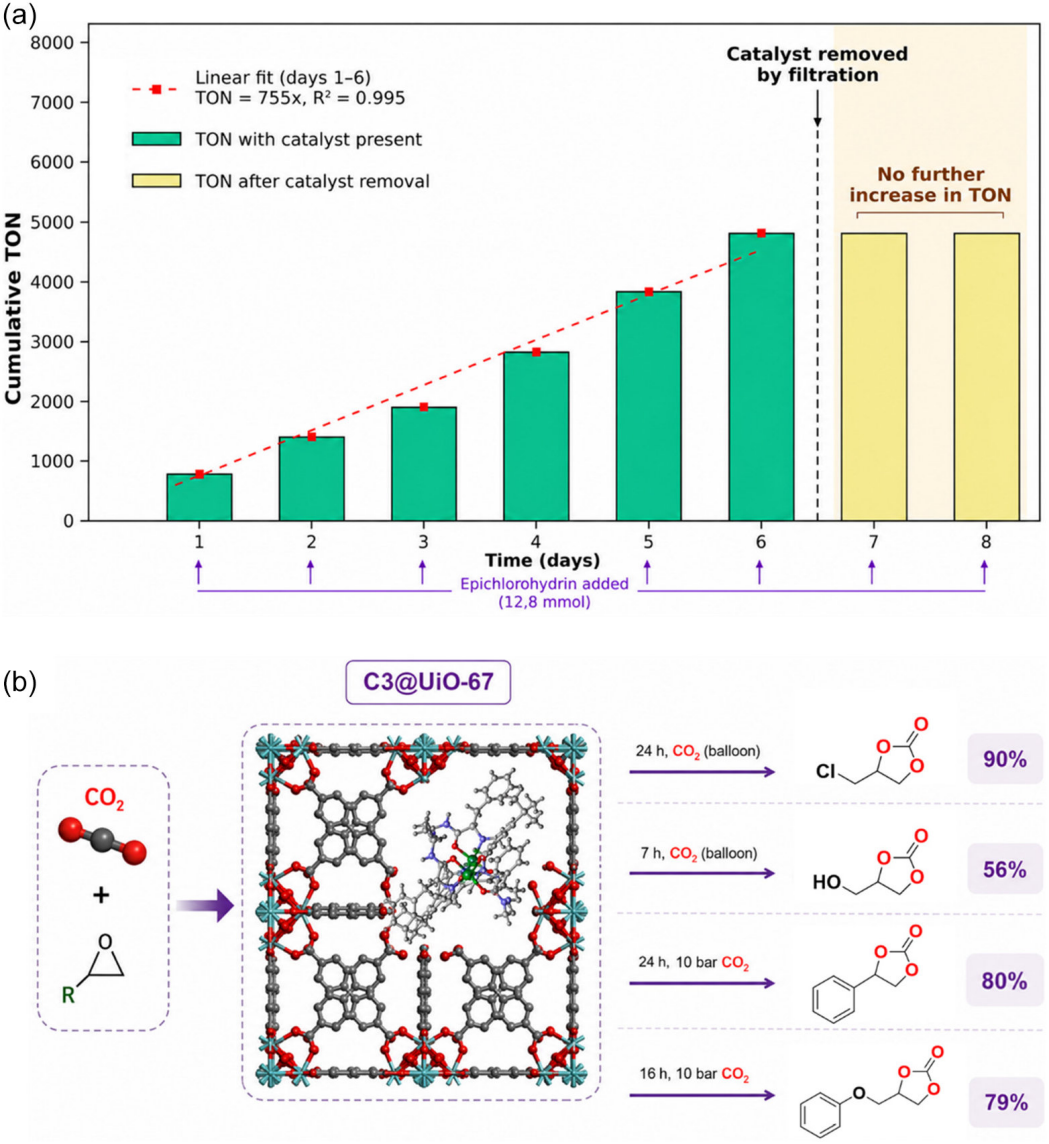
(a) TON evolution during successive additions of epichlorohydrin. TON values were calculated relative to the molar amount of the bimetallic Zn macrocyclic complex. The experimental TON values show an approximately linear increase over six reaction cycles. After removal of the solid catalyst by filtration on Day 6, no further increase in TON was observed. (b) Cycloaddition products and conversions at defined reaction time and pressure studied using C3@UiO‐67 as catalyst. Reaction conditions: 7.85 mg of C3@UiO‐67/mmol epoxide; 80 °C. Yields determined by ^1^H‐NMR.

To evaluate the stability and recyclability of the catalyst under the reaction conditions, successive additions of epichlorohydrin were performed over several catalytic cycles while monitoring the evolution of the TON (Figure 8a). A progressive increase in TON was observed after each substrate addition, following a linear trend during the first six consecutive reaction cycles. A yield for the same time intervals of ca. 75% was consistently obtained after each cycle. This behavior indicates that the catalyst remains active throughout the consecutive cycles without significant deactivation. The experimental TON values remained close to the theoretical values, considering the yield obtained in the first cycle as the basis for the calculation, further supporting the sustained catalytic performance. An accumulative TON of ca. 4800 were achieved. This is a significant value considering that no additional co‐catalysts are required. After the sixth cycle, the solid catalyst was separated from the reaction mixture by filtration to determine whether the active catalytic species remained associated with the solid phase or had leached into solution. No additional increase in yield or TON was observed during the following 2 days, demonstrating that the catalytic activity ceased upon removal of the solid material. These results indicate that the active species are retained on the solid catalyst and support the heterogeneous nature of the catalytic system. By contrast, recovery of the finely divided material by filtration, washing, and drying resulted in lower activity upon reuse and a decrease in the Zn/Zr mass ratio from 0.0211 to 0.0116 despite the absence of significant changes in its FTIR spectrum. These results suggest that the main limitation arises from recovery‐associated loss or reorganization of the immobilized macrocycle and/or iodide species rather than from intrinsic deactivation during the catalytic operation.

Additionally, to further demonstrate the synthetic utility and substrate tolerance of the optimized **C3@UiO‐67** catalyst, the system was applied to the CO_2_ cycloaddition of a family of terminal epoxides under the same reaction conditions (80 °C, CO_2_balloon, and solventless) (Figure 8b). Thus, the reactivity of glycidyl oxide renders conversions of 74% in 7 h with a selectivity for the corresponding cyclic carbonate of 56%. Although the reaction with the sluggish styrene oxide led only to a 9% yield in 24 h and no conversion was observed for phenyl glycidyl oxide (see the Supporting Information for product characterization). When the cycloaddition reaction was carried out under pressure (10 bar), the reactions with styrene and phenyl glycidyl oxide afforded yields of 80% and 79% after 24 and 16 h of the reaction, respectively (Figure 8b).

## 3 Conclusion

We have successfully developed a novel class of heterogeneous synzymatic materials through the supramolecular immobilization of bioinspired zinc macrocycles within a **UiO‐67** MOF. The design of the macrocyclic ligands was strategically controlled from the molecular level via a solvent‐directed macrocyclization; furthermore, the solid‐state characterization of the bimetallic **C3** complex showed the preorganization of hydrogen‐bond‐stabilized “naked” iodides. Moreover, experimental and computational analyses of the hybrid materials revealed a defect‐driven encapsulation mechanism; although this localized framework perturbation reduced the intrinsic porosity of the MOF, it successfully accommodated the bulky complexes and conferred remarkable thermal stabilization. Ultimately, the **C3@UiO‐67** hybrid presented an outstanding catalytic performance for the additive‐free cycloaddition of CO_2_ to epoxides at ambient pressure, exhibiting broad substrate tolerance under harder reaction conditions. The enhanced activity observed for **C3@UiO‐67** arises from potent cooperative effects between the localized CO_2_ capture by the secondary amines and the nucleophilic iodide species. Such synergistic behavior is highly reminiscent of enzymatic systems, where the optimal 3D arrangement of the protein backbone (the **UiO‐67** structure) provides a confining binding pocket for the active cofactor (the **C3** macrocycle). Thereby, these findings pave the way for the rational design of robust, bioinspired multicomponent supramolecular platforms for sustainable and efficient chemical transformations.

## Funding

This study was supported by MICINN‐FEDER‐AEI (PID2022‐142897OA‐I00 and PID2024‐159264OB‐C21), the Generalitat Valenciana (CISEJI/2023/78, CIACIF/2023/229), and MCIN/AEI/10.13039/501100011033 (RYC2020‐028681‐I).

## Conflicts of Interest

The authors declare no conflicts of interest.

## Supporting information

Detailed experimental procedures, figures, and characterization data are available online in the Supporting Information section.

## Data Availability

All the data supporting the findings of this work are available from the corresponding authors on request. Crystallographic data for the structure reported in this paper have been deposited in the Cambridge Structural Database with the CCDC Number 2550493.
