# Supplementary material for: Bioinspired Zinc Macrocycles Confined in UiO‐67: A Heterogeneous Synzyme for Sustainable CO2 Valorization
Source: ChemSusChem. 2026 Aug 27;19(17):e71010. doi: 10.1002/cssc.71010 (PMC13520813; doi:10.1002/cssc.71010)
Supplement: Supplementary file 1 [file CSSC-19-e71010-s001.pdf]

# Bioinspired Zinc Macrocycles Confined in UiO-67: A Heterogeneous Synzyme for Sustainable $CO_2$ Valorization

J. E. Chiva[a], J. E. Sánchez-Velandia[a], F. Esteve[b], F. G. Cirujano[a], C. Vicent[c], N. Martín[a], B. Altava[a]*, E. García-Verdugo[a]*

## Content

1. SYNTHESIS AND CHARACTERIZATION OF PSEUDOPEPTIDIC MACROCYCLIC COMPLEXES ........ 1
2. CHARACTERIZATION OF SUPPORTED CATALYSTS ON UiO-67 ........ 8
3. DETERMINATION OF CATALYTIC ACTIVITY ........ 13
4. EXPERIMENTAL METHODOLOGY ........ 16
4.1. General: ........ 16
4.2. Synthesis of the macrocyclic pseudopeptidic ligands ........ 16
4.3. Synthesis of the organometallic pseudopeptidic complexes ........ 17
4.4. Synthesis and impregnation of MOF UiO-67 ........ 17
4.1.1 Characterization of the materials and supramolecular ligands/complexes: ........ 17
4.5. General procedure for the $CO_2$ cycloaddition to epichlorohydrin: ........ 18
4.6. Procedure for stability and recyclability evaluation: ........ 18
4.7. General procedure for FTIR conversion determination: ........ 18
4.8. General procedure for $^1$H NMR conversion determination: ........ 18
4.9. Crystal structure: ........ 19
4.10. Computational Conformer Analysis: ........ 19
5. SUBSTRATES CHARACTERIZATION ........ 20
6. CRYSTAL STRUCTURE OF C3 ........ 34

## 1. SYNTHESIS AND CHARACTERIZATION OF PSEUDOPEPTIDIC MACROCYCLIC COMPLEXES

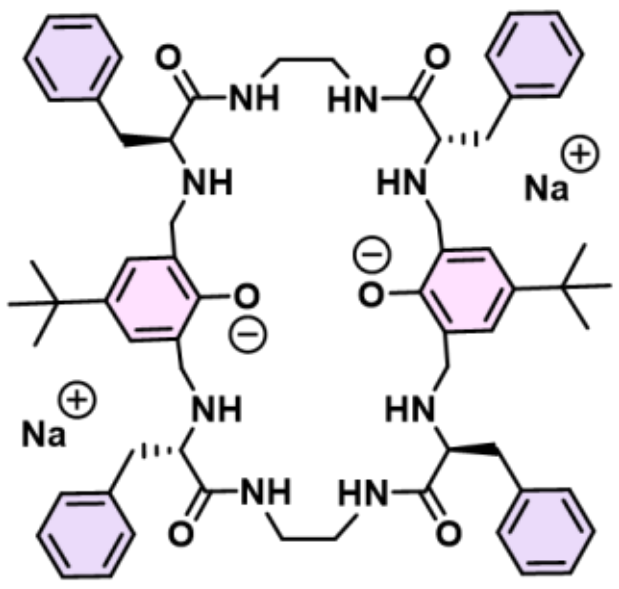

L1

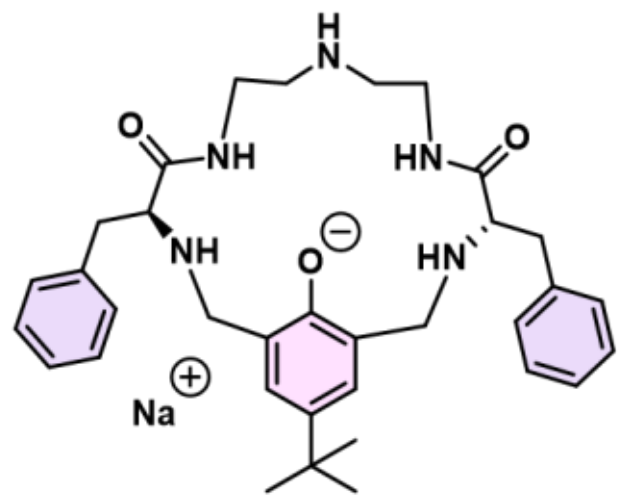

L2

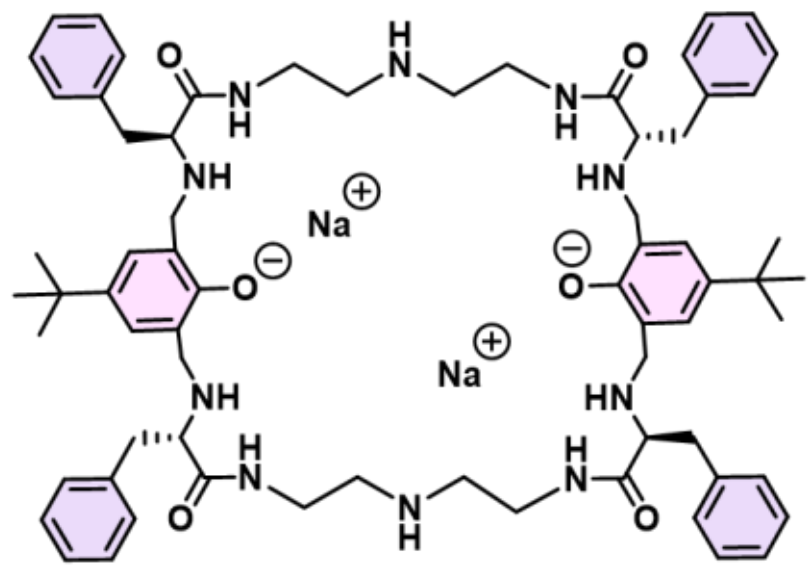

L3

**Figure S1.** Structures of the pseudopeptidic macrocyclic ligands described in this work.

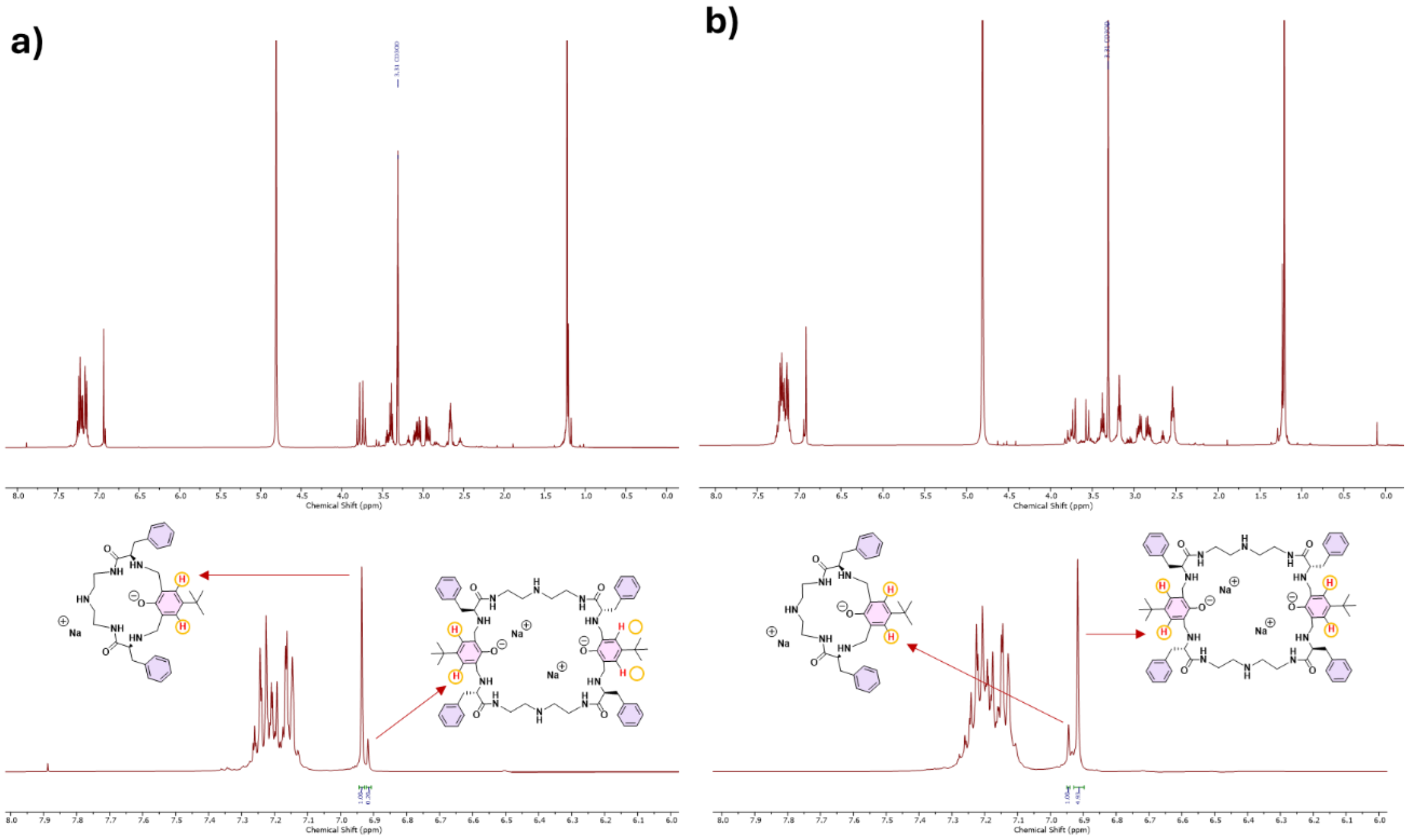


**Figure S2.** a) $^1$H NMR (400 MHz, 25ºC) spectra of crude product after the imine condensation of **BA** with **DFP** in $CHCl_3$, followed by in situ imine reduction. b) $^1$H NMR (400 MHz, 25ºC) spectra of crude product after the imine condensation of **BA** with **DFP** in ethanol, followed by in situ imine reduction.

a)

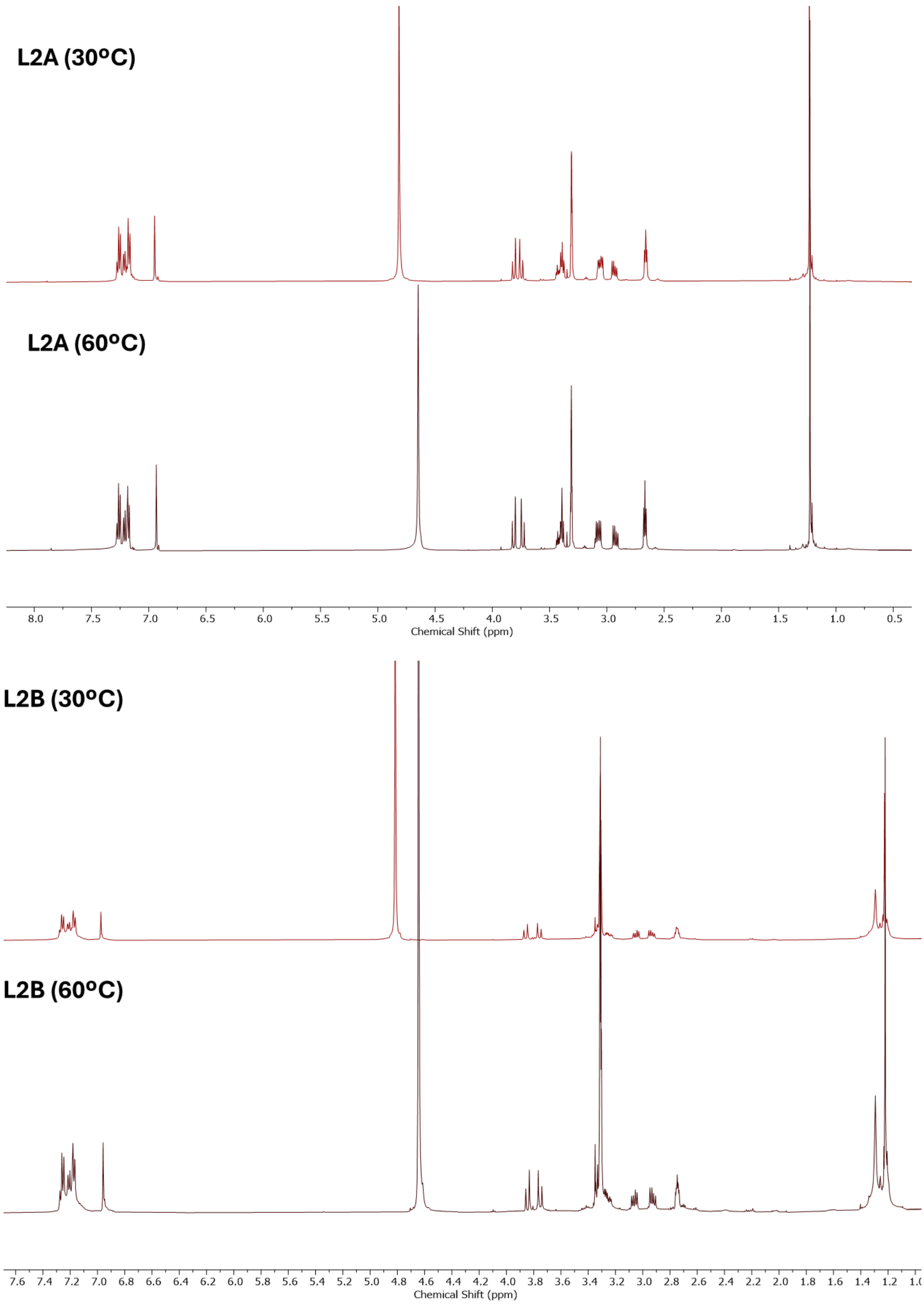
L2A (30ºC)
L2A (60ºC)
8.0 7.5 7.0 6.5 6.0 5.5 5.0 4.5 4.0 3.5 3.0 2.5 2.0 1.5 1.0 0.5
Chemical Shift (ppm)
L2B (30ºC)
L2B (60ºC)
7.6 7.4 7.2 7.0 6.8 6.6 6.4 6.2 6.0 5.8 5.6 5.4 5.2 5.0 4.8 4.6 4.4 4.2 4.0 3.8 3.6 3.4 3.2 3.0 2.8 2.6 2.4 2.2 2.0 1.8 1.6 1.4 1.2
Chemical Shift (ppm)

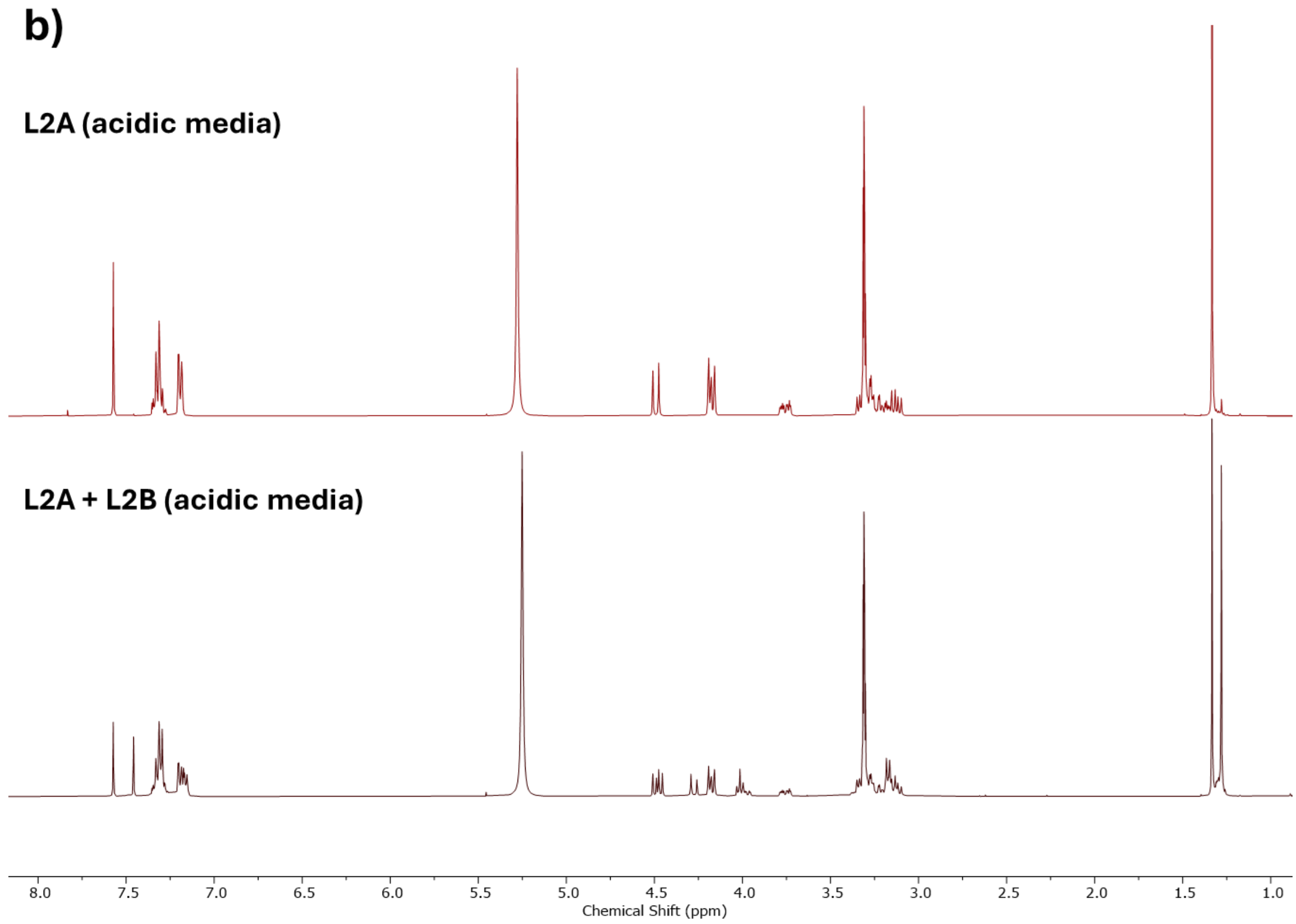


**Figure S3**. a) $^{1}H$ NMR (500 MHz, $CD_3OD$) spectra of both conformers **L2A** and **L2B** at 30 ºC and 60 °C. b) $^{1}H$ NMR (400 MHz, $CD_3OD$, 25 ºC) spectra of conformer **L2A** and a mixture of **L2A** and **L2B** in acidic media ($CF_3CO_2D$).

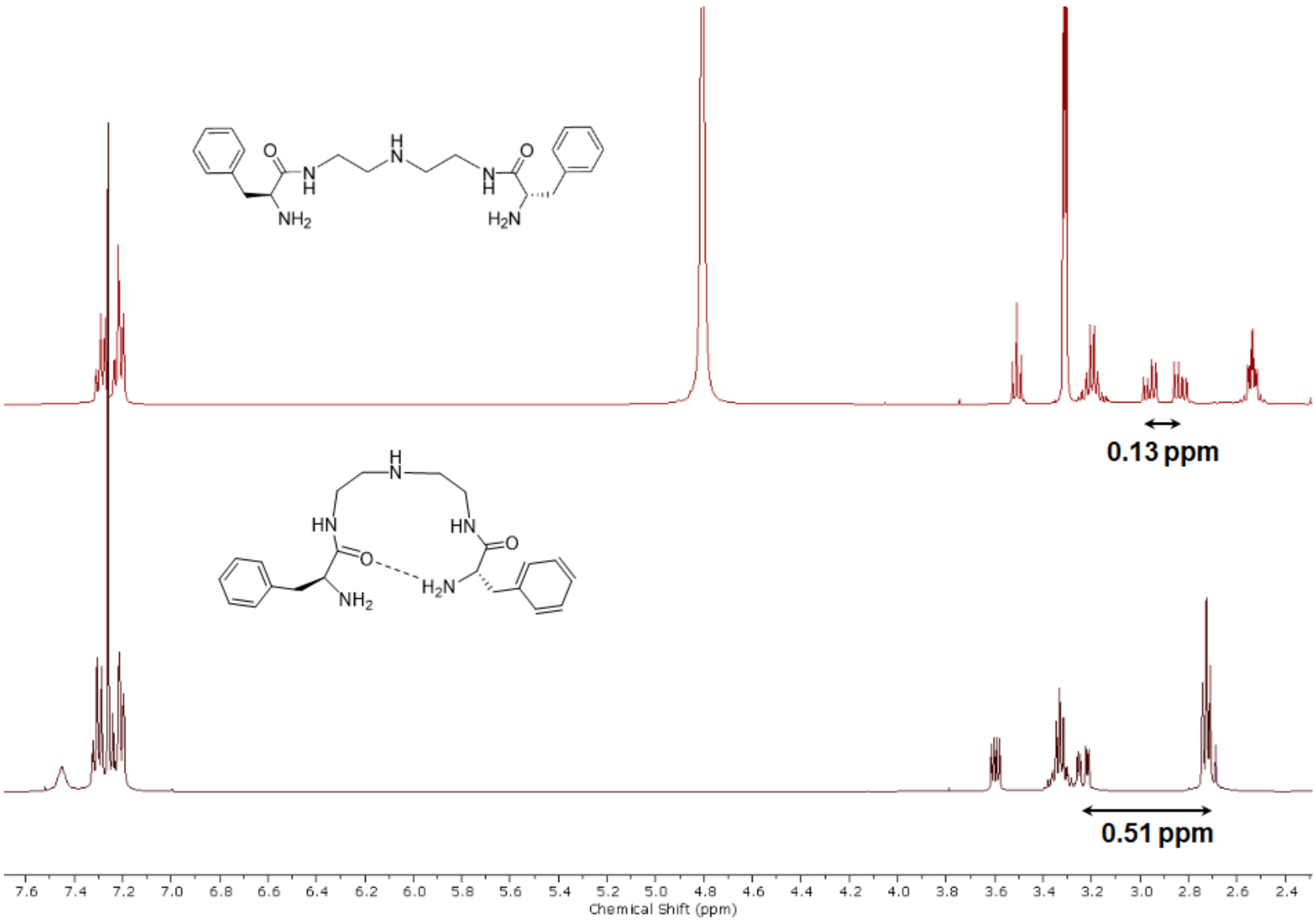


**Figure S4.** $^{1}$H NMR (400 MHz, 25ºC) spectra of bis(aminoamide) **BA** recorded in $CDCl_3$ (down) and $CD_3OD$ (down), highlighting the diastereotopic protons of the benzylic methylene group.

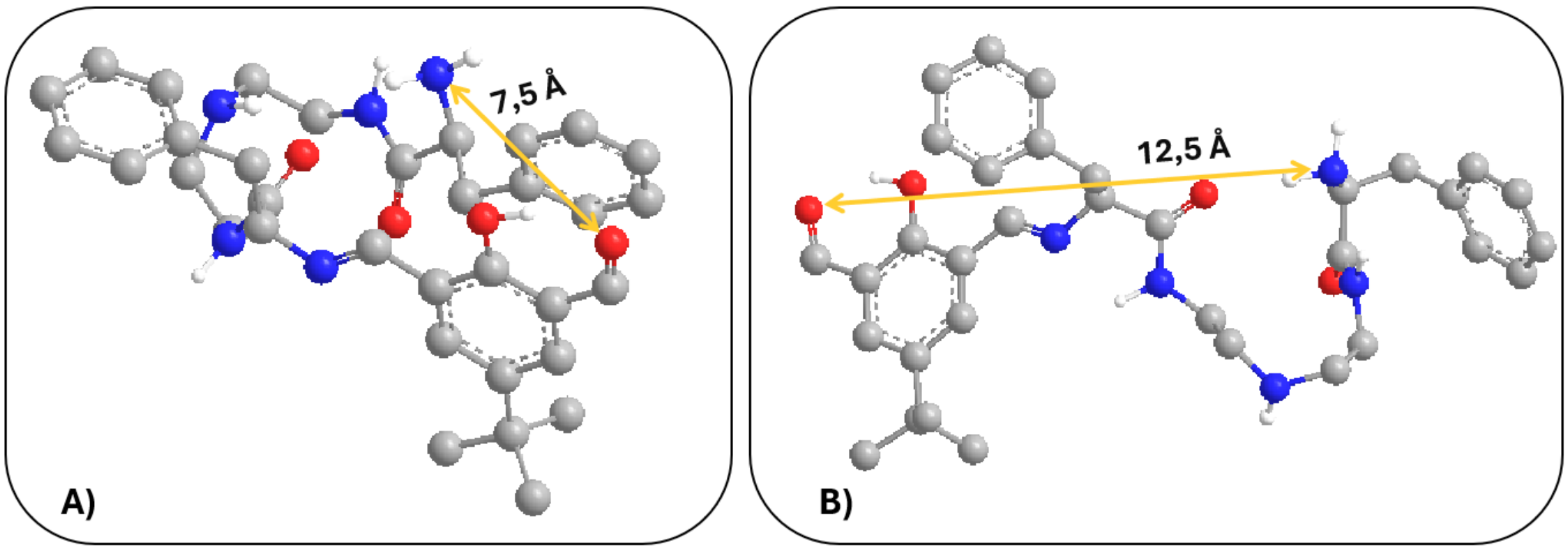


**Figure S5.** Equilibrium conformation of the reaction intermediate for the cyclization of **BA** with **DFP**. A) in chloroform and B) in ethanol. GFN2-xTB (CREST program).

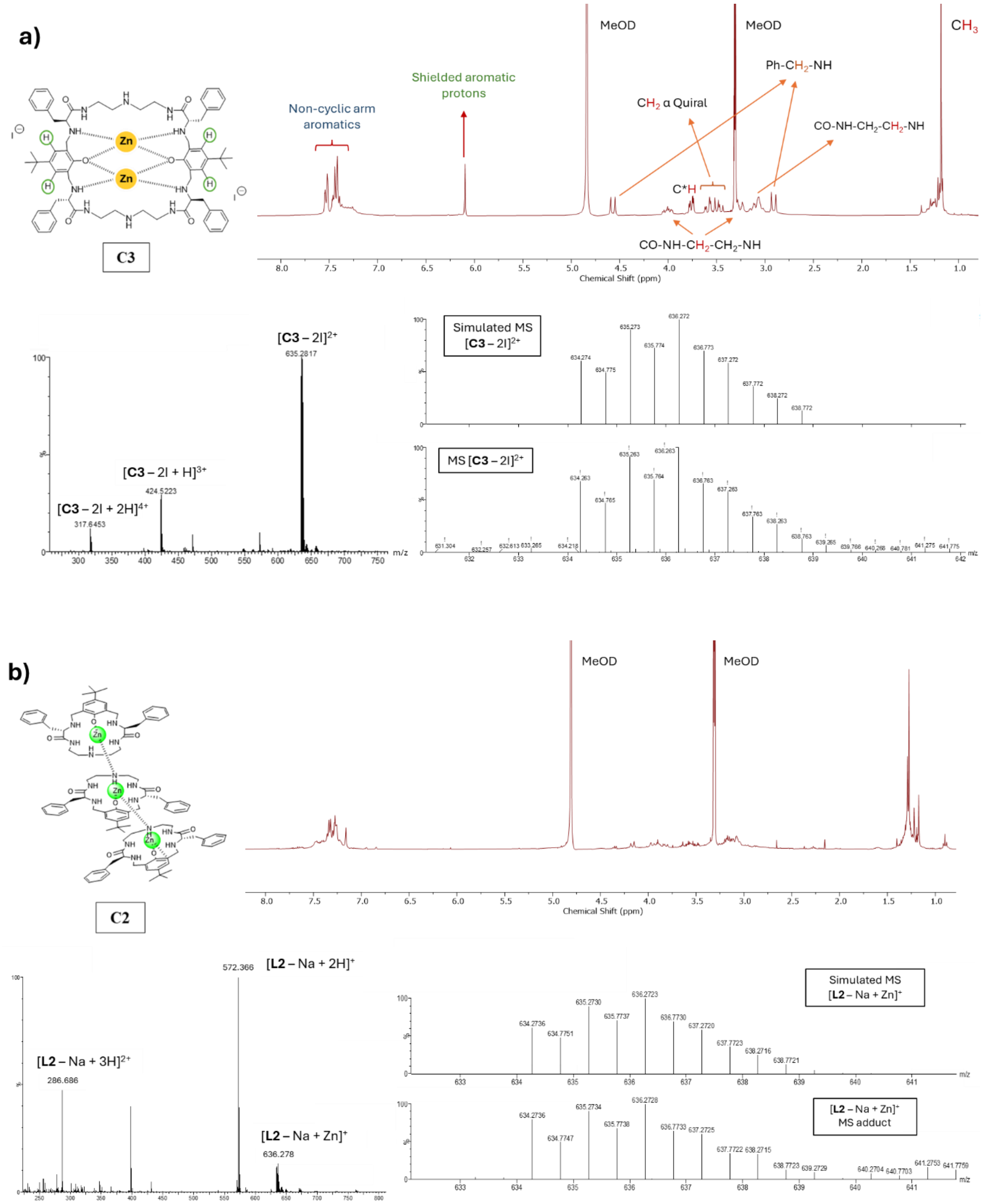


**Figure S6.** $^1$H NMR (400 MHz, $CD_3OD$, 25 ºC) and ESI-MS spectra of a) bimetallic complex **C3** and b) supramolecular polymeric complex **C2**.

## 2. CHARACTERIZATION OF SUPPORTED CATALYSTS ON UiO-67

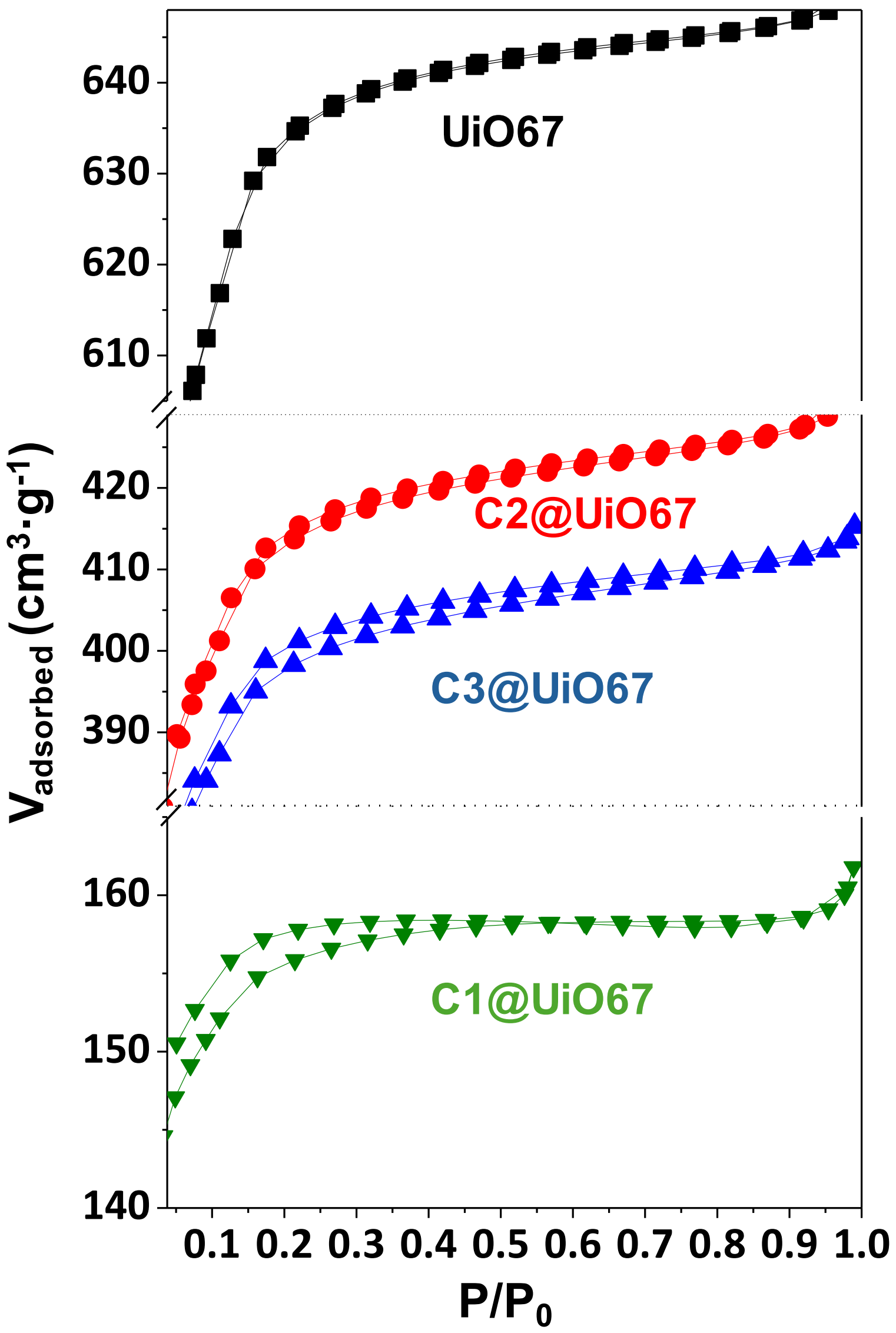


**Figure S7**. $N_2$ physisorption isotherms at 77K, highlighting the hysteresis loop attributed to defect-induced mesoporosity.

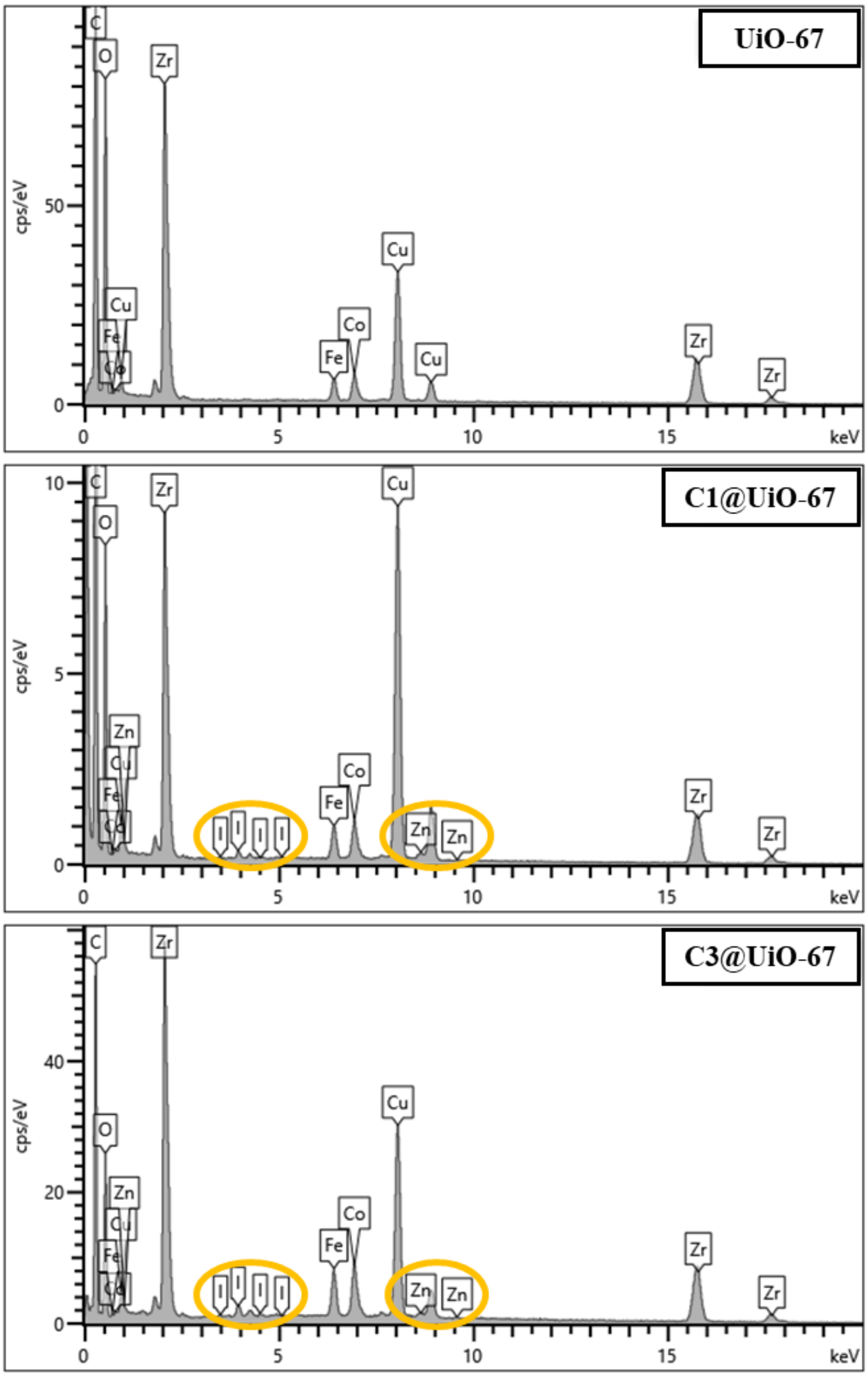


**Figure S8.** EDX spectra of the MOF **UiO-67** and the impregnated MOFs **C1@UiO-67** and **C3@UiO-67**.

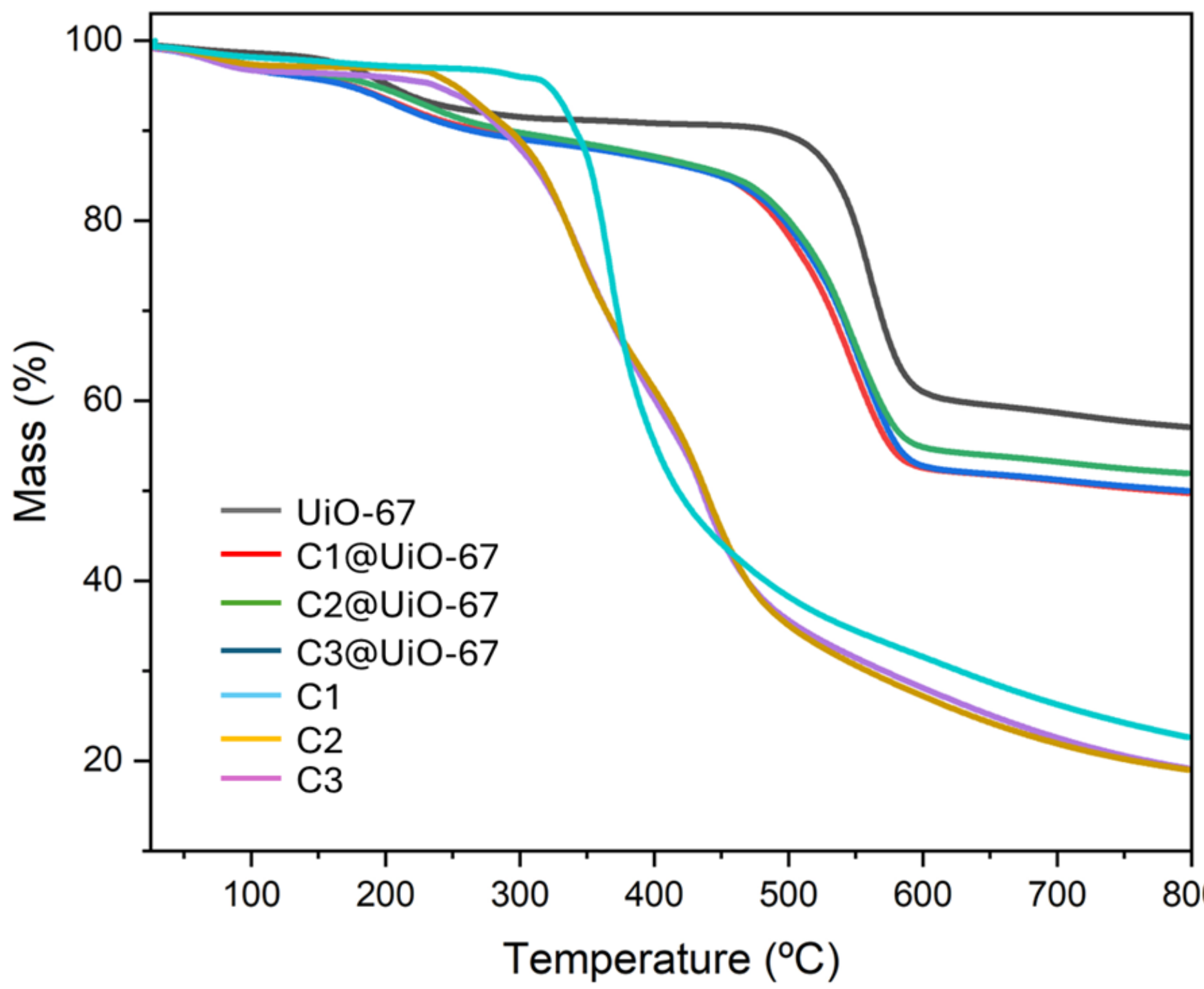


**Figure S9.** TGAs for all the different complexes and MOFs studied.

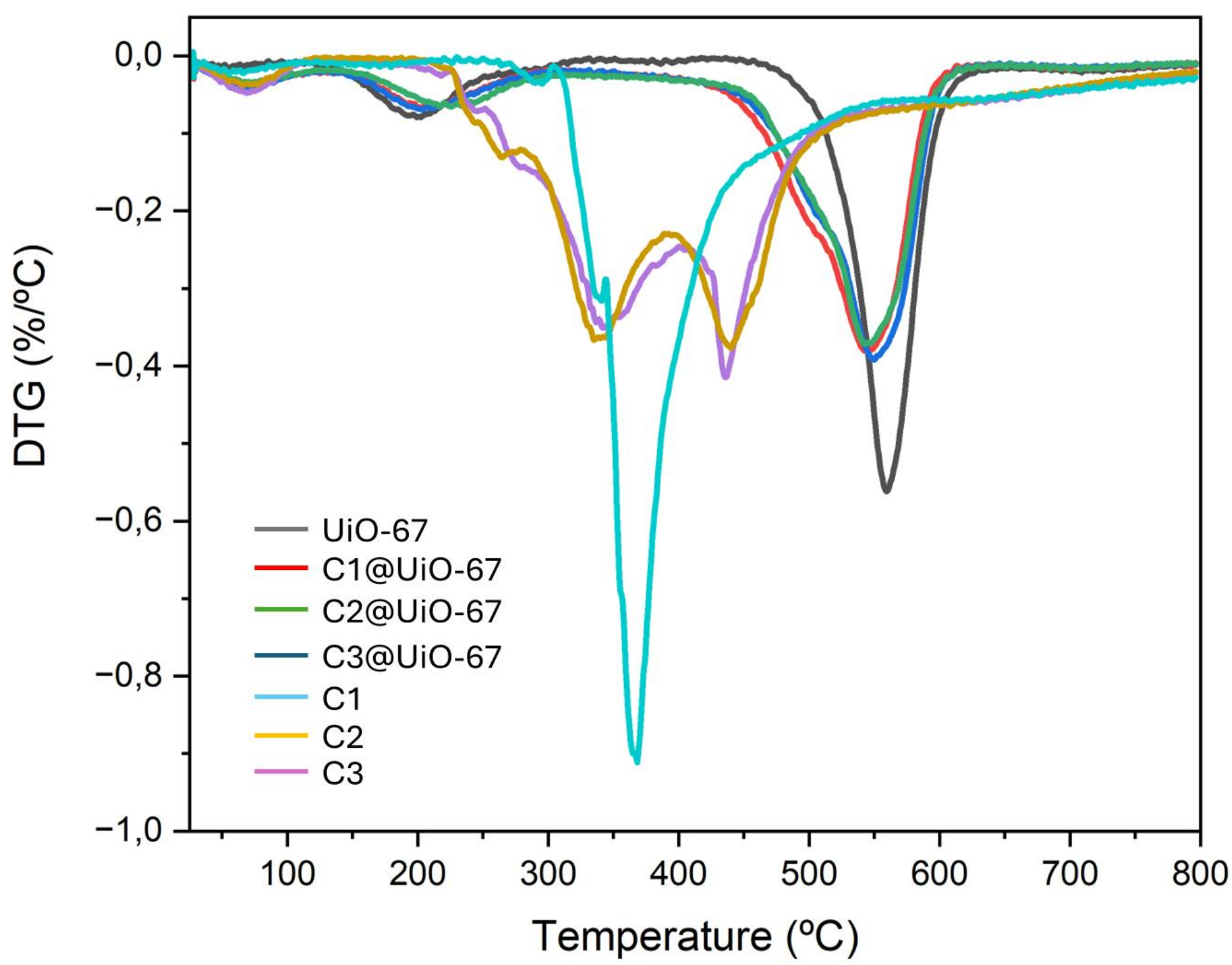


**Figure S10.** DTGs for all the different complexes and MOFs studied.

**Table S1.** Textural properties of UiO-67 and the impregnated derivatives derived from $N_2$ / $CO_2$ adsorption-desorption measurements.

| | $N_2$ adsorption-desorption Studies | | | | $CO_2$ adsorption-desorption Studies | |
|---|---|---|---|---|---|---|
| **Sample** | **BET Surface ($m^2/g$)** | **Micropore Volume ($cm^3/g$)** | **Micropore Area ($m^2/g$)** | **External Surface ($m^2/g$)** | **Qads (mmol/g)** | **Eads (kJ/mol)** |
| UiO-67 | 2457 | 0.95 | 2383 | 74 | 3.26 | 17.59 |
| C2@UiO-67 | 1585 | 0.62 | 1534 | 51 | 2.27 | 17.67 |
| C3@UiO-67 | 1531 | 0.60 | 1481 | 50 | 2.32 | 17.74 |
| C1@UiO-67 | 609 | 0.24 | 601 | 8 | 1.34 | 18.35 |

The adsorption energy, $E_{ads}$, was calculated using the Dubinin-Radushkevich method, according to the following equation. The required parameters were obtained from the $CO_2$ adsorption isotherms measured experimentally.

$$\boldsymbol{logV = logV_0 - B\left(\frac{T}{\beta}\right)^2 log^2\left(\frac{P_0}{P}\right)}$$

where $V$ represents the volume of $CO_2$ adsorbed at a given pressure, and $V_0$ corresponds to the micropore capacity, i.e., the maximum volume of adsorbate that can be accommodated within the micropores of the material. $T$ is the adsorption temperature (273 K), $\beta$ is the affinity factor (β = 0.48 for $CO_2$), and $P_0/P$ is the inverse of $P/P_0$ (relative pressure).

This model is applied in the low relative pressure range ($P/P_0 < 0.01$), where micropore filling is predominant. The parameter $B$ is related to the adsorption energy and can be expressed by equation:

$$\boldsymbol{B} = -2.303\left(\frac{\boldsymbol{R}^2}{\boldsymbol{E}_{\boldsymbol{ads}}^2}\right)$$

from which the characteristic adsorption energy ($E_{ads}$) can be derived. $R$ is the universal gas constant (R = 8.314 J/mol·K).

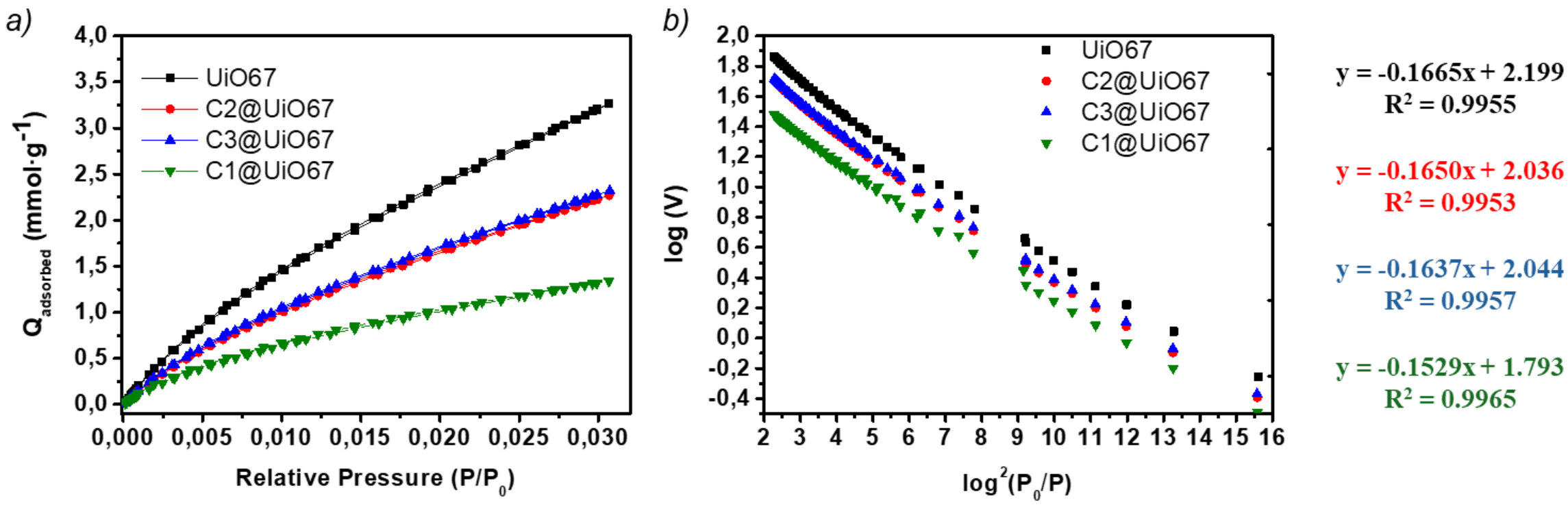


**Figure S11.** (a) $CO_2$ physisorption isotherms of pristine **UiO-67** and the hybrid **macrocycle-UiO-67** systems at 273K. (b) Dubinin-Radushkevich plot (log V vs. [$\log^2(P_0/P)$]) used for the determination of the adsorption energy ($E_{ads}$).

## 3. DETERMINATION OF CATALYTIC ACTIVITY

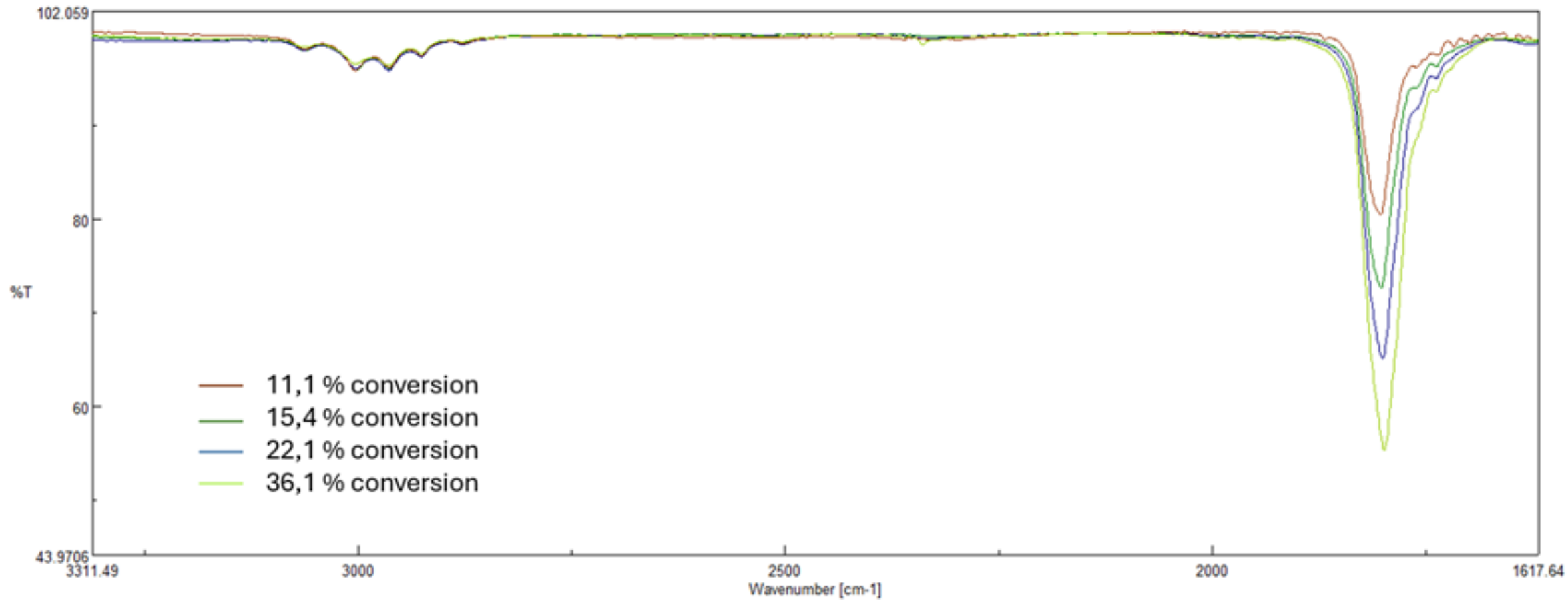


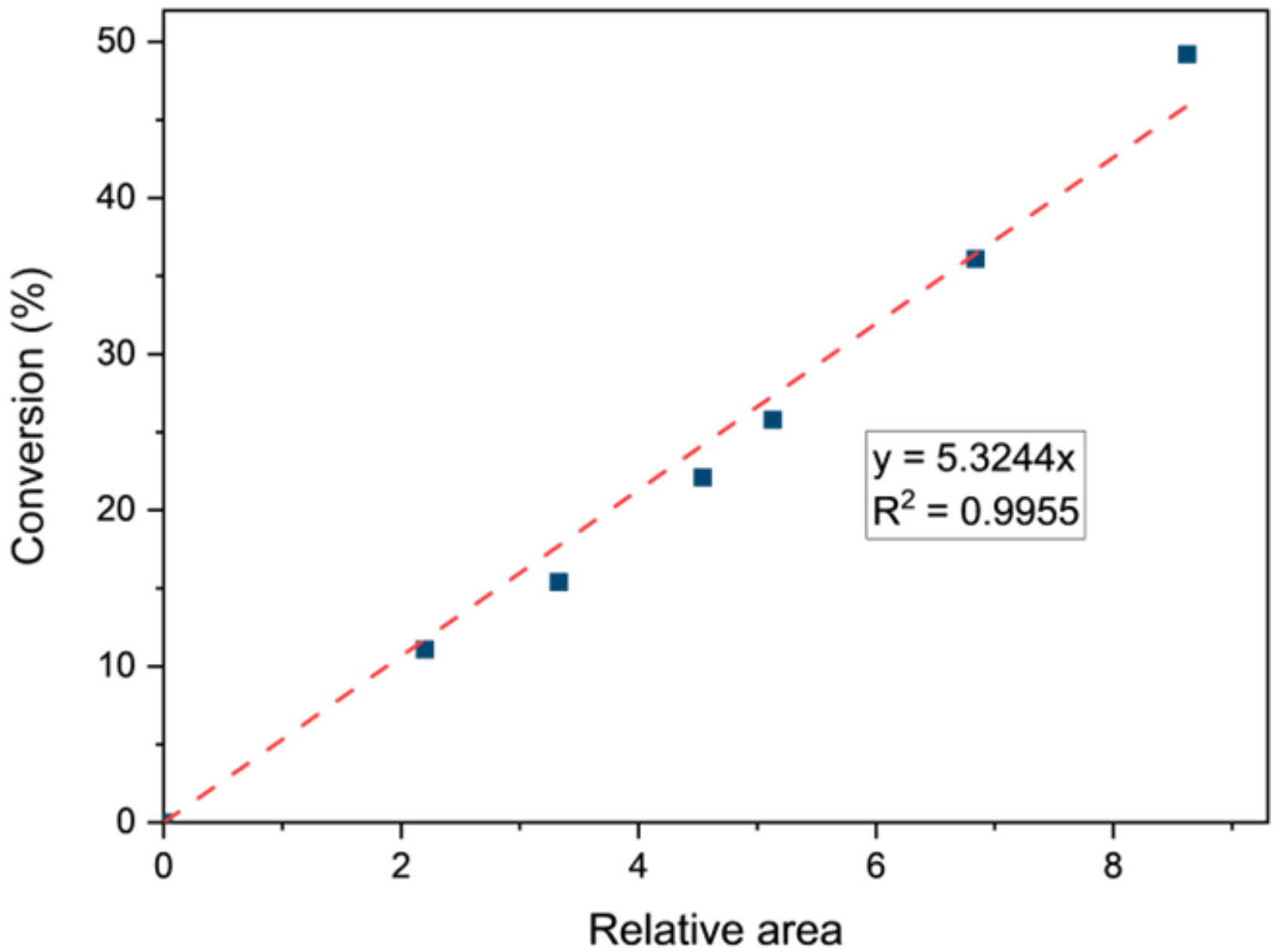


**Figure S12.** FTIR spectra of the cycloaddition reactions of $CO_2$ to epichlorohydrin at different reaction time and the calibration curve obtained from the relative area between the CO st band at 1799 $cm^{-1}$ and the CH st bands between 2797 and 3124 $cm^{-1}$.

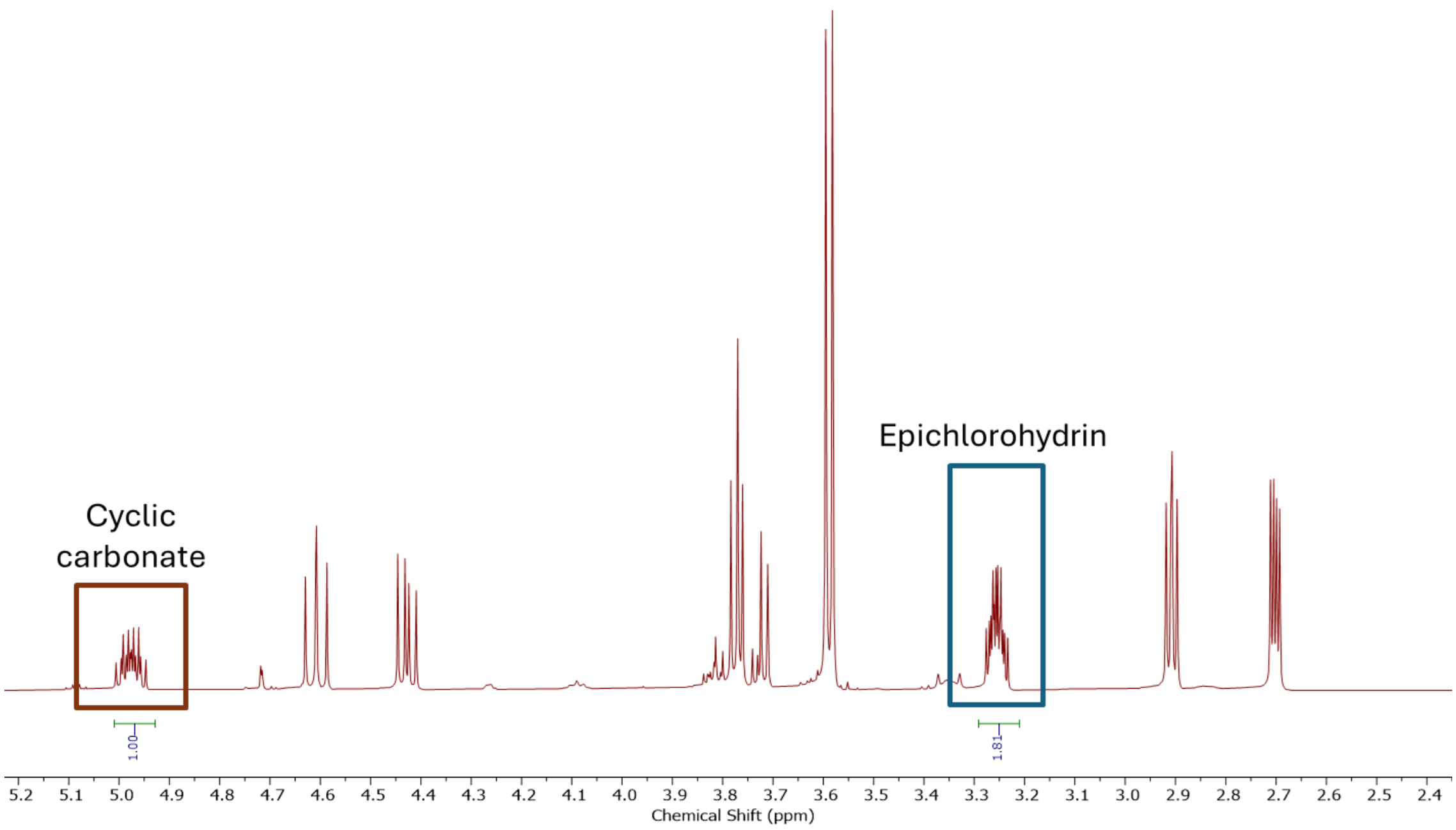


**Figure S13.** $^{1}$H NMR of the cycloaddition reaction between epichlorohydrin and $CO_2$ at 80 ºC and $CO_2$ balloon after 90 min using 1 mol% tetrabutylammonium chloride as catalyst.

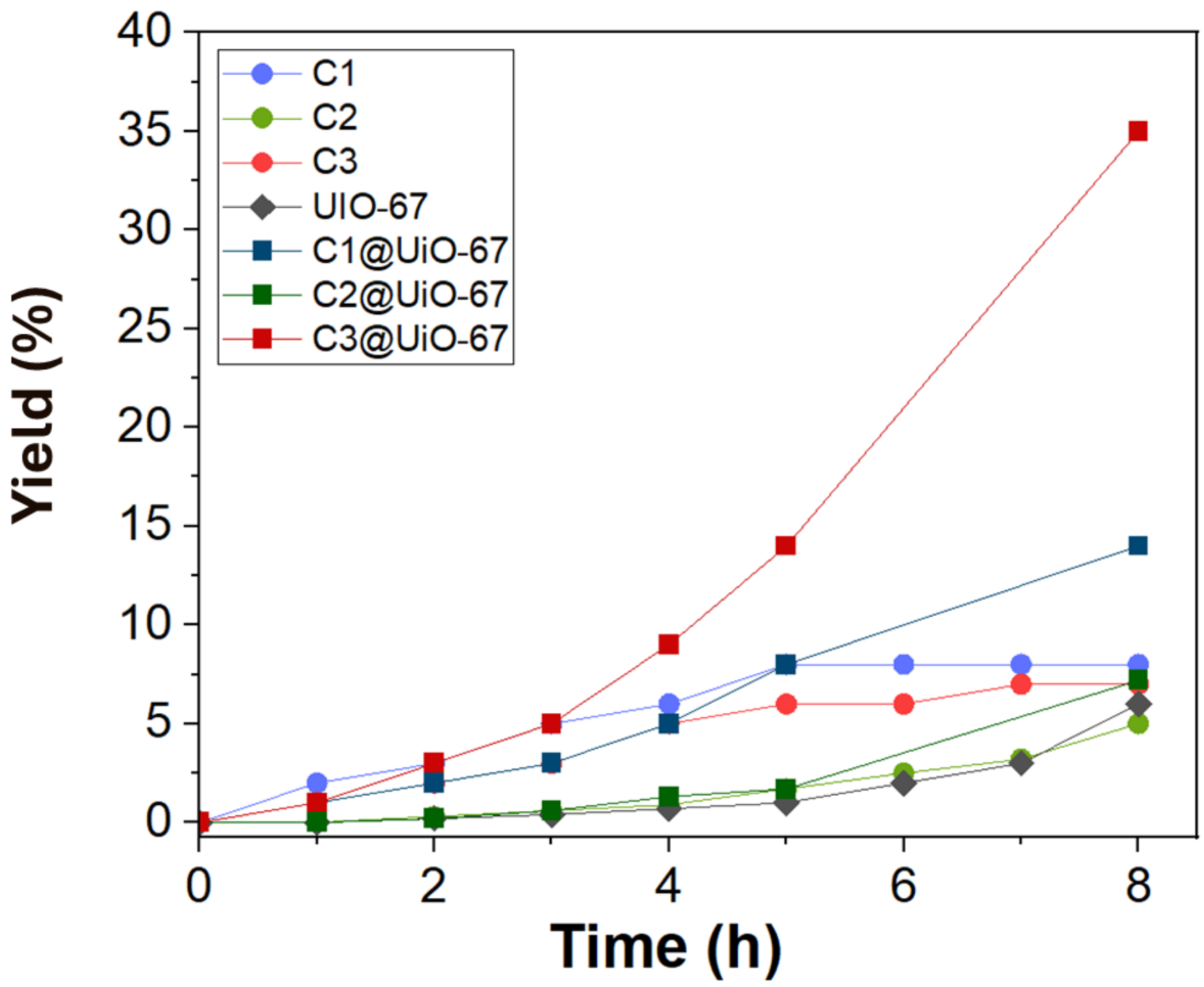


**Figure S14.** 8h FTIR kinetic profiles for all the tested catalysts and materials.

**Table S2.** Yield, TON and TOF for the cycloaddition reaction studied using homogeneous and heterogeneuos catalyst.

| Homogeneous catalyst | | | | Heterogeneous hybrid catalyst | | | | | | |
|---|---|---|---|---|---|---|---|---|---|---|
| Metal Complex | 8h | | | Catalyst | 8h | | | 24h | | |
| | Yield | TON | TOF ($h^{-1}$) | | Yield | TON | TOF ($h^{-1}$) [a] | Yield | TON[a] | TOF ($h^{-1}$) [a] |
| **C1** | 8 | 78 | 10 | **C1@UiO-67** | 14 | 137[a] | 17[a] | 82 | 805[a] | 33[a] |
| **C2** | 5 | 49 | 6 | **C2@UiO-67** | 7 | 71[a] | 9[a] | n.d. | -- | -- |
| **C3** | 7 | 68 | 9 | **C3@UiO-67** | 35 | 343[a] | 43[a] | 90 | 883[a] | 37[a] |
| | | | | **UiO-67** | 6 | 2[b] | 0.3[b] | 71 | 25[b] | 1[b] |

[a] Related to Zn; [b] Related to Zr. n.d not determined.

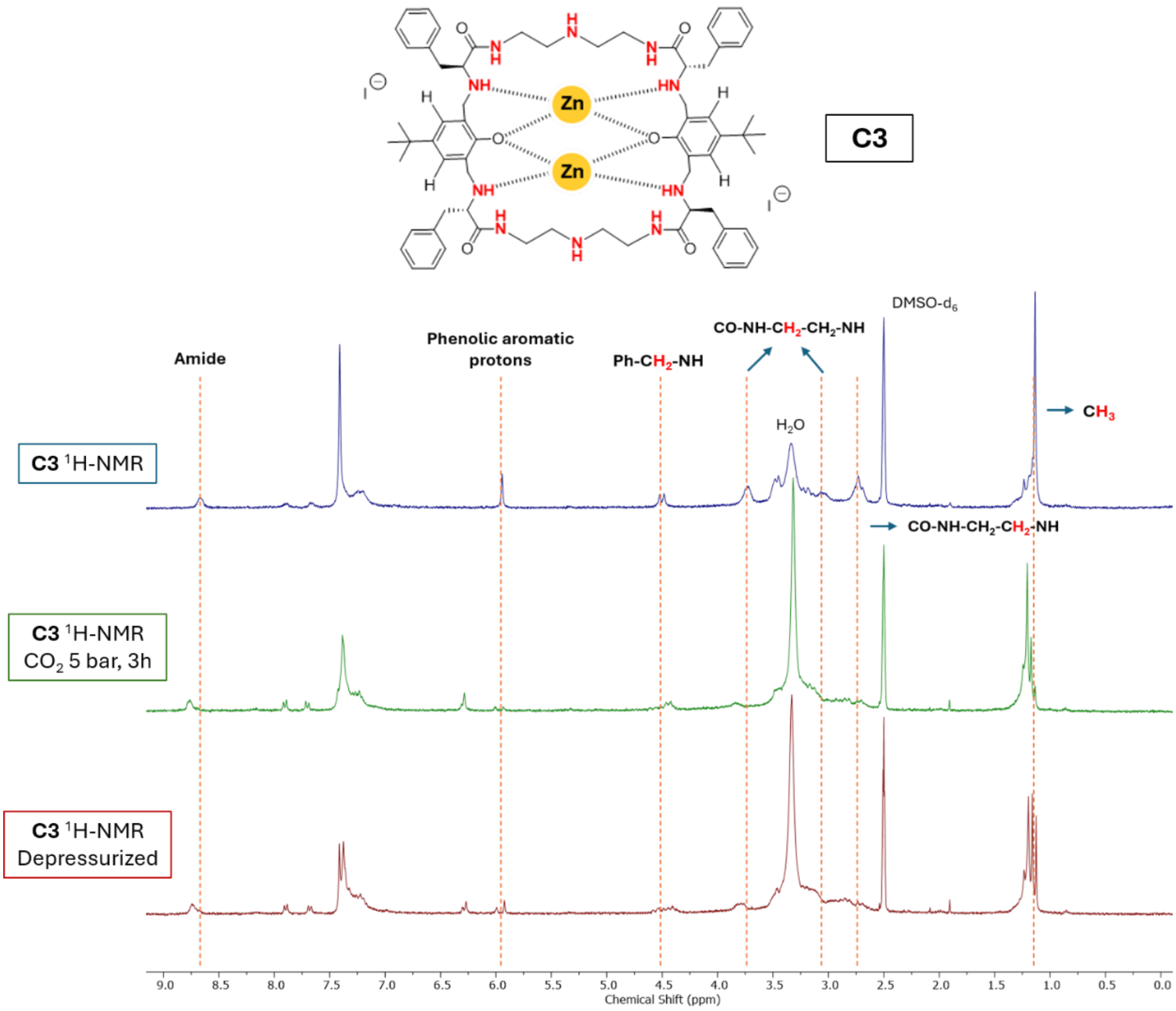


**Figure S15.** Reversible interaction of **C3** with $CO_2$ monitored by $^{1}H$ NMR (300 MHz, DMSO-$d_6$, 25 ºC).

## 4. EXPERIMENTAL METHODOLOGY

**4.1. General:** All reagents were commercially sourced from Sigma-Aldrich, Thermo Fisher Scientific, Fluorochem, and TCI, and used without additional purification. Spectroscopic grade solvents were purchased from Scharlau and Thermo Fisher Scientific, while deuterated solvents were obtained from Euriso-TOP. Open-chain pseudopeptidic precursors were synthesized according to previously reported procedures [1]. Macrocyclic pseudopeptidic ligand **L1** was prepared according to the procedure described in the literature [2].

### 4.2. Synthesis of the macrocyclic pseudopeptidic ligands

*Synthesis of* ***L2****:* In a round-bottom flask, the bis(amino amide) (**BA**,0.593 g, 1.492 mmol) was dissolved in chloroform (150 mL). Under constant stirring, a solution of 4-tert-butyl-2,6-diformylphenol (**DFP**, 0.320 g, 1.492 mmol) in chloroform (150 mL) was added (final concentration: 5 mM). After 3.5 h of reaction, methanol (50 mL) and an excess of $NaBH_4$ (8 eq.) were added. The resulting white suspension was stirred at room temperature for 4 h. The suspension was then filtered off, and the filtrate was concentrated to dryness under reduced pressure. The resulting solid was washed twice with basic aqueous $Na_2CO_3$ solution, recovering the solid by centrifugation. After drying under vacuum, the crude product was purified by silica gel column chromatography using DCM:MeOH (9:1) as eluent to afford **L2A** (0.321 g, 0.541 mmol, 36% yield) as a pale-yellow powder. A small fraction of **L2B** (0.032 g, 4% yield) was also obtained as a powder with the same appearance. Characterization for **L2A** was as follows: $^1$H NMR (400 MHz, $CDCl_3$, 25 ºC) δ = 7.73 (s, 2H), 7.34 – 7.17 (m, 10H), 6.86 (s, 2H), 3.88 (d, *J* = 12.3 Hz, 2H), 3.64 – 3.54 (m, 4H), 3.47 (dd, *J* = 8.3, 4.5 Hz, 2H), 3.24 (dd, *J* = 14.0, 4.6 Hz, 2H), 3.19 – 3.10 (m, 2H), 2.89 (dd, *J* = 13.9, 8.3 Hz, 2H), 2.78 – 2.60 (m, 4H), 1.21 (s, 9H); $^{13}$C{$^1$H} NMR (400 MHz, $CDCl_3$, 25 ºC) δ = 172.62, 153.53, 142.61, 137.42, 129.40, 128.81, 127.02, 126.19, 123.70, 63.74, 50.34, 48.96, 39.39, 38.64, 31.59; FTIR (ATR): 3304, 2952, 1648, 1526, 1484, 1453, 1215, 1107, 743, 701 $cm^{-1}$; MS-ESI(+) m/z: 286.7 ($[M+3H-Na]^{2+}$), 572.4 ($[M+2H-Na]^{+}$). Characterization for **L2B** was as follows: $^1$H NMR (400 MHz, $CDCl_3$, 25 ºC) δ = 7.70 (s, 2H), 7.34 – 7.17 (m, 10H), 6.86 (s, 2H), 3.80 (d, J = 12.4 Hz, 2H), 3.65 (d, J = 12.4 Hz, 2H), 3.55 – 3.42 (m, 4H), 3.37 – 3.30 (m, 2H), 3.26 (dd, J = 13.9, 4.6 Hz, 2H), 2.92 (dd, J = 13.9, 8.2 Hz, 2H), 2.84 – 2.69 (m, 4H), 1.21 (s, 9H). $^{13}$C{$^1$H} NMR (400 MHz, $CDCl_3$) δ = 172.94, 153.57, 142.68, 137.44, 129.38, 128.89, 127.06, 126.48, 123.58, 63.22, 50.46, 49.30, 39.36, 38.18, 34.07, 31.61. FTIR (ATR): 3304, 2952, 1648, 1526, 1484, 1453, 1215, 1107, 743, 701 $cm^{-1}$; MS-ESI(+) m/z: 572.4 ($[M+2H-Na]^{+}$).

*Synthesis of* ***L3****:* In a round-bottom flask, the bis(amino amide) (**BA**,0.493 g, 1.240 mmol) was dissolved in ethanol (78 mL). Under constant stirring, a solution of 4-tert-butyl-2,6-diformylphenol (**DFP**, 0.266 g, 1.240 mmol) in ethanol (78 mL) was added (final concentration: 8 mM). After 3 h of reaction, an excess of $NaBH_4$ (8 eq.) was added. The resulting white suspension was stirred at room temperature for 4 h. The suspension was then filtered, and the filtrate was concentrated to dryness under reduced pressure. The resulting solid was washed twice with basic aqueous $Na_2CO_3$ solution, and the solid was recovered by centrifugation. After drying under vacuum and at 60 ºC, **L3** was obtained as a pale-yellow powder (0.608 g, 83% yield). Characterization for **L3** was as follows: $^1$H NMR (400 MHz, $CD_3OD$, 25ºC) δ = 7.30 – 7.08 (m, 20H), 6.92 (s, 4H), 3.72 (d, *J* = 13.0 Hz, 4H), 3.56 (d, *J* = 13.2 Hz, 4H), 3.38 (t, *J* = 7.2 Hz, 4H), 3.18 (t, *J* = 6.1 Hz, 8H), 2.94 (dd, *J* = 13.5, 6.7 Hz, 4H), 2.83 (dd, *J* = 13.5, 7.5 Hz, 4H), 2.54 (t, J = 6.2 Hz, 8H), 1.21 (s, 18H). $^{13}$C{$^1$H} NMR (400 MHz, $CD_3OD$, 25ºC) δ = 175.76, 154.78, 142.73, 138.79,

130.34, 129.54, 127.77, 126.54, 124.92, 64.23, 50.20, 49.13, 40.47, 39.91, 34.77, 32.06. FTIR (ATR): 3304, 2952, 1648, 1526, 1484, 1453, 1215, 1117, 743, 701 $cm^{-1}$. MS-ESI(+) m/z: 286.4 ($[M+6H-2Na]^{4+}$), 381.9 ($[M+5H-2Na]^{3+}$), 572.4 ($[M+4H-2Na]^{2+}$), 1143.7($[M+3H-2Na]^{+}$).

### 4.3. Synthesis of the organometallic pseudopeptidic complexes

Pseudopeptidic metal complex **C1** was prepared according to the well-described procedure in the literature.[2]

*Synthesis of* ***C2****:* Initially, two separate solutions (**A**: 1 eq of **L2** in 1 mL of methanol; **B**:2 eq of $ZnI_2$ in 1 mL of methanol) were prepared and added under static conditions (**A** over **B**). Then, the mixture was stirred for 30 min and subsequently filtered off through a 0.45 µm nylon membrane filter. After concentrating the product to dryness under reduced pressure, it was washed with Milli-Q water. The solid was then recovered by centrifugation and dried under vacuum and at 60 ºC to afford a pale-yellow powder, 0.063 g, 0.083 mmol, 95% yield. Characterization for **C2** was as follows: FTIR (ATR): 3226, 2952, 1624, 1548, 1479, 1453, 1300, 1217, 1083, 828, 744, 701 $cm^{-1}$. MS-ESI(+) m/z: 286.7 ($[L2-Na+3H]^{2+}$), 572.4 ($[L2-Na+2H]^{+}$), 633.3 ($[M-I]^{+}$).

*Synthesis of* ***C3****:* **C3** was synthesized following the protocol described for **C2** but using **L3** as ligand (yellowish powder, 0.218 g, 0.143 mmol, 85% yield). Characterization was as follows: $^{1}H$ NMR (300 MHz, $CD_3OD$, 25ºC) δ = 7.58 – 7.50 (m, 8H), 7.48 – 7.38 (m, 12H), 6.10 (s, 4H), 4.57 (d, $J$ = 13.4 Hz, 4H), 4.09 – 3.93 (m, 4H), 3.82 – 3.69 (m, 4H), 3.65 – 3.40 (m, 8H), 3.29 – 3.20 (m, 4H), 3.18 – 2.99 (m, 8H), 2.91 (d, $J$ = 13.6 Hz, 4H), 1.18 (s, 18H). $^{13}C\{^{1}H\}$ NMR (400 MHz, $CD_3OD$, 25ºC) δ = 178.80, 162.34, 139.58, 138.99, 131.50, 130.95, 130.18, 128.39, 121.85, 57.80, 53.37, 50.20, 38.97, 38.56, 34.50, 32.14. FTIR (ATR): 3226, 2952, 1624, 1548, 1479, 1453, 1288, 1217, 1097, 828, 744, 701 $cm^{-1}$. MS-ESI(+) m/z: 317.6 ($[M+2H-2I]^{4+}$), 424.5 ($[M+H-2I]^{3+}$), 635.3 ($[M-2I]^{2+}$).

### 4.4. Synthesis and impregnation of MOF UiO-67

*Synthesis of* ***UiO-67****:* In a reaction vial, $ZrCl_4$ (0.448 g, 1.922 mmol) and 4,4'-biphenyl dicarboxylic acid, abbreviated as BPDC (0.480 g, 1.922 mmol) were weighed and combined. DMF (72 mL) and glacial acetic acid (8 mL) were added, and the resulting white suspension was sonicated for 5 min. The mixture was then heated at 120 °C for 72 h. After this time, the solid was separated by centrifugation. The white solid was washed twice with DMF and twice with methanol. Finally, the solid was recovered by centrifugation and dried under reduced pressure at 60 °C to afford a white powder (0.644g, 95% yield).

*General procedure for MOF wet impregnation:* In a vial, MOF UiO-67 (186 mg) was weighed and placed. A 12.2 mM solution of the corresponding macrocyclic complex in THF (1 mL) was added. The resulting mixture was stirred under magnetic agitation (600 rpm) for 4 h. The suspension was then allowed to dry at room temperature, and any residual solvent was removed under reduced pressure at 60 °C.

**4.1.1 Characterization of the materials and supramolecular ligands/complexes:** NMR spectra were obtained using Bruker Avance III HD 400 (400 MHz for $^{1}H$ and 100 MHz for $^{13}C\{^{1}H\}$) and Agilent VNMR System 500 MHz instruments (500 MHz for $^{1}H$ and 125 MHz for $^{13}C\{^{1}H\}$). Processing and analysis of the NMR data were carried out with MestReNova 14 software. Chemical shifts are reported in parts per million (ppm). To prevent solvent evaporation and concentration variations, NMR samples were sealed in tubes fitted with Teflon caps. Coupling constants (J) are expressed in hertz (Hz). Signal

multiplicities are indicated as singlet (s), doublet (d), triplet (t), doublet of doublets (dd), and multiplet (m). Unless stated otherwise, all spectra were recorded at 25 ºC. FTIR spectra were acquired using a Jasco instrument equipped with an attenuated total reflection (ATR) accessory. High-resolution mass spectra (HRMS) with electrospray ionization (ESI) were obtained via direct injection on a Waters Xevo G2-XS QTof spectrometer. Thermogravimetric analyses (TGA) were performed using a Mettler Toledo TGA-DSC system. Microscopy images were obtained with a Hitachi S4800 SEM-FEG (scanning electron microscopy- field emission gun) equipment, with an accelerating voltage of 20kV, coupled with an Energy Dispersive X-ray (EDX) detector. Transmission electron microscopy (TEM) images were captured with a 200 kV TEM (Jeol JEM 2100plus, 0.23 nm) with CMOS camera (2k × 2k). X-ray diffraction patterns were obtained with a D4 Endeavor, Bruker-AXS powder diffractometer. Gas physisorption analysis was performed in an AUTOSORB-6 volumetric equipment and the MOF activation (150ºC, 3h) was performed in an AUTOSORB DEGASSER, both from QUANTACHROME INSTRUMENTS, using $N_2$ (77K) or $CO_2$ (273 K).

**4.5. General procedure for the $CO_2$ cycloaddition to epichlorohydrin:** In a 25 mL two-necked round-bottom flask, 100 mg of MOF-impregnated catalyst (0.0065 mmol of macrocyclic complex for the reactions without MOF) were weighed, and epichlorohydrin (1 mL, 12.8 mmol) was added. The system was purged first with $N_2$ and then with $CO_2$. After the purges, $CO_2$ pressure was maintained throughout the reaction using a balloon. The mixture was stirred at 300 rpm and heated to 80 °C using a sand bath. Aliquots of the reaction mixture were taken at the desired times to determine conversion.

**4.6. Procedure for stability and recyclability evaluation:** The cycloaddition of $CO_2$ to epichlorohydrin was carried out following the procedure described above. After 24 h of reaction, an aliquot was withdrawn and the reaction yield was determined by $^1H$ NMR spectroscopy. Subsequently, epichlorohydrin (1 mL, 12.8 mmol) was added again, and the $CO_2$ balloon was replaced with a fresh one. This procedure was repeated over six consecutive catalytic cycles.

Finally, to assess whether the active catalytic species remained associated with the solid phase or had leached into solution, the resulting suspension was filtered through a 0.45 µm nylon membrane filter, affording a clear orange solution. Two additional catalytic cycles were then carried out in the absence of the solid catalyst following the procedure described above.

**4.7. General procedure for FTIR conversion determination:** The conversion was determined using a calibration curve obtained from the $CO_2$ cycloaddition to epichlorohydrin catalyzed by tetrabutylammonium chloride. To construct the calibration curve, the relative area of the band between 1722–1888 $cm^{-1}$, corresponding to the C=O stretching vibration of the formed carbonate, was measured with respect to the band between 2797–3124 $cm^{-1}$, which remains unaffected by the reaction. The conversion for each calibration point was independently determined by $^1H$ NMR spectroscopy (Figure S12). This calibration was applied for conversion values below 50%.

**4.8. General procedure for $^1H$ NMR conversion determination:** $^1H$ NMR spectra were recorded by diluting 10 µL of the reaction crude with 490 µL of $CDCl_3$. The signals δ (parts per million) for epichlorohydrin are as follows: 3.57 (d, 2H), 3.29 – 3.19 (m, 1H), 2.89 (dd, 1H), 2.68 (dd, 1H). The signals δ (parts per million) for the cyclic carbonate are as follows: 5.03 – 4.93 (m, 1H), 4.61 (dd, 1H), 4.43 (dd, 1H), 3.82 – 3.68 (m, 2H). Conversion was measured by $^1H$ NMR integration, considering the signals at 3.29 – 3.19

for epichlorohydrin and 5.03 – 4.93 for the cyclic carbonate as follows: c (%) = [I cyclic carbonate / (I cyclic carbonate + I epiclorohydrin)]x 100 (Figure S13). $^{1}$H NMR was applied for the construction of the IR calibration curve and for the determination of conversion above 50%.

**4.9. Crystal structure:** Single crystals suitable for X-ray crystallography were obtained by slow evaporation of a chloroform-methanol mixture solution of **C3**. A suitable crystal was selected and mounted on a SuperNova Dual Cu at zero Atlas diffractometer. Data collection, integration, and reduction were performed using CrysAlisPro software. The structure was solved automatically using AutoChem and refined with Olex2 (v1.5). Artwork representations were generated using Mercury software. The refined structure has been deposited at the CCDC under deposition number 2550493. See (Figure S43 and Table S3).

**4.10. Computational Conformer Analysis:** Equilibrium conformations of the reaction intermediate for the cyclization of **BA** with **DFP** were explored using the CREST (Conformer-Rotamer Ensemble Sampling Tool) program at the GFN2-xTB level of theory [3,4]. Conformational sampling was performed using the default iMTD-GC workflow with an implicit solvation model (GBSA) for chloroform and ethanol respectively. The "-quick" option was employed to accelerate the sampling. The resulting conformers were subsequently optimized at the same level of theory, and redundant structures were removed based on energy and structural similarity criteria. Molecular graphics and artwork representations were generated using Mercury and Materials Studio software.

## 5. SUBSTRATES CHARACTERIZATION

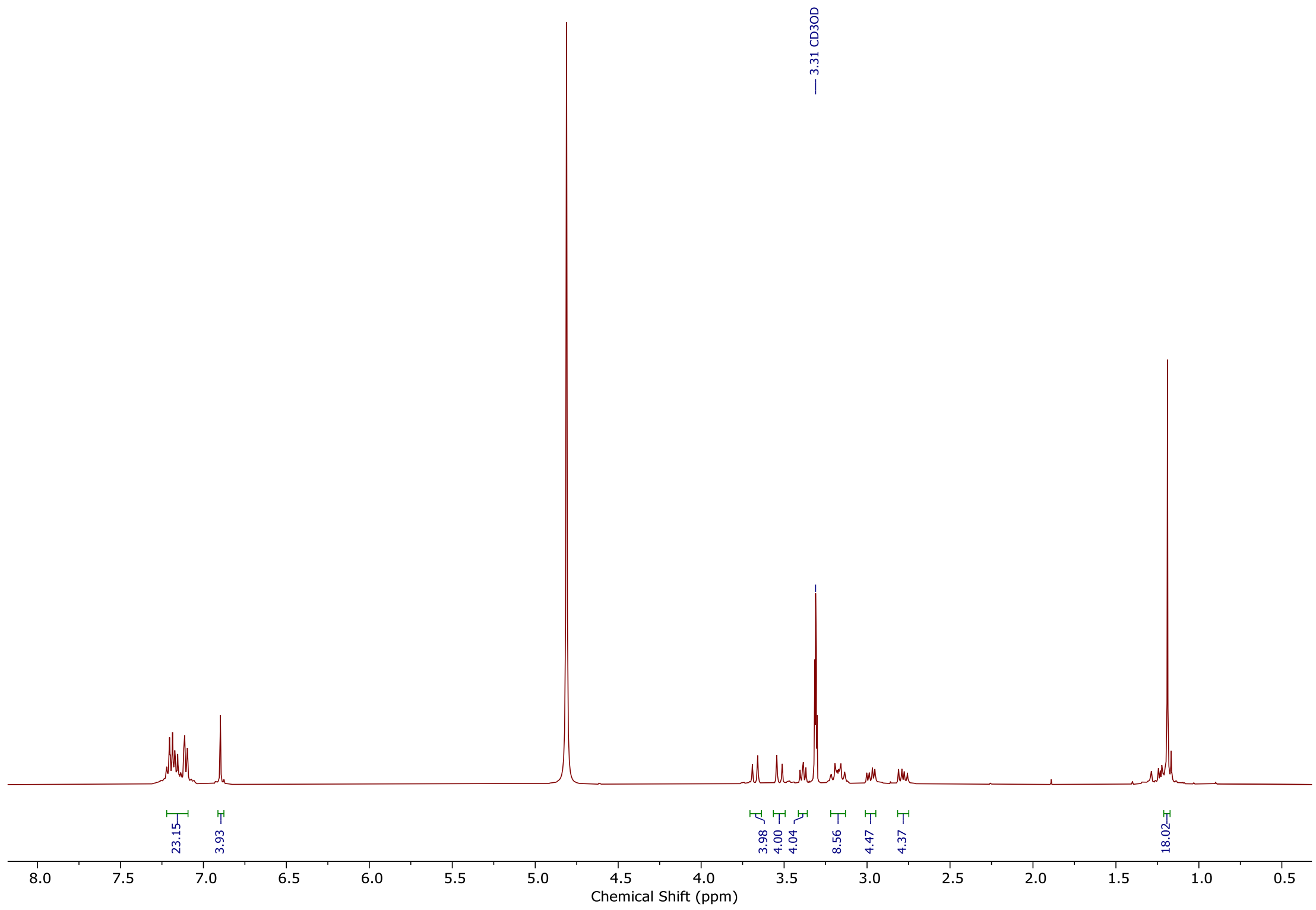


**Figure S16**. $^{1}$H NMR (400 MHz, $CD_3OD$, 25ºC) for **L1** (5 mM).

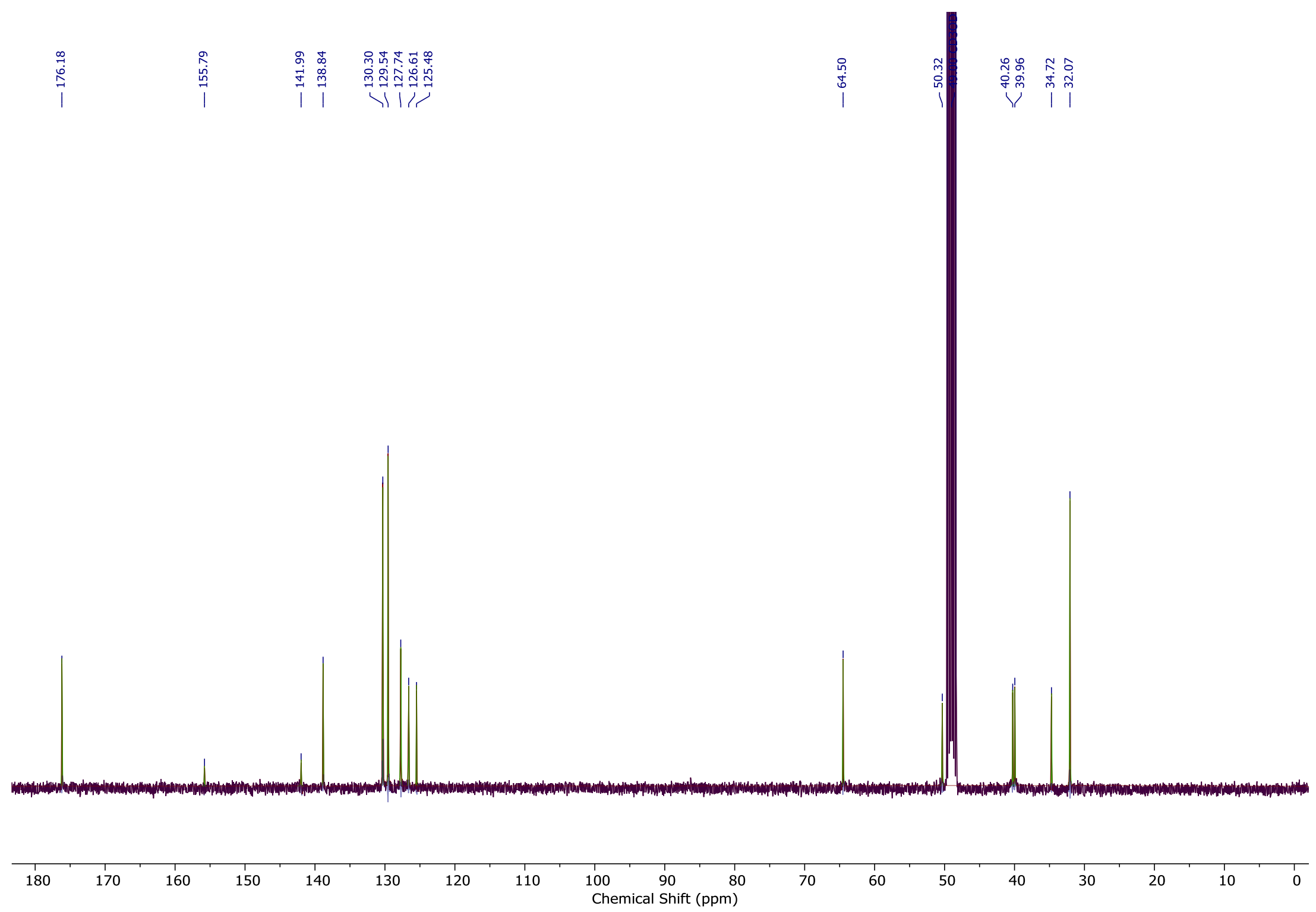


**Figure S17**. $^{13}$C NMR (400 MHz, $CD_3OD$, 25ºC) for **L1** (5 mM).

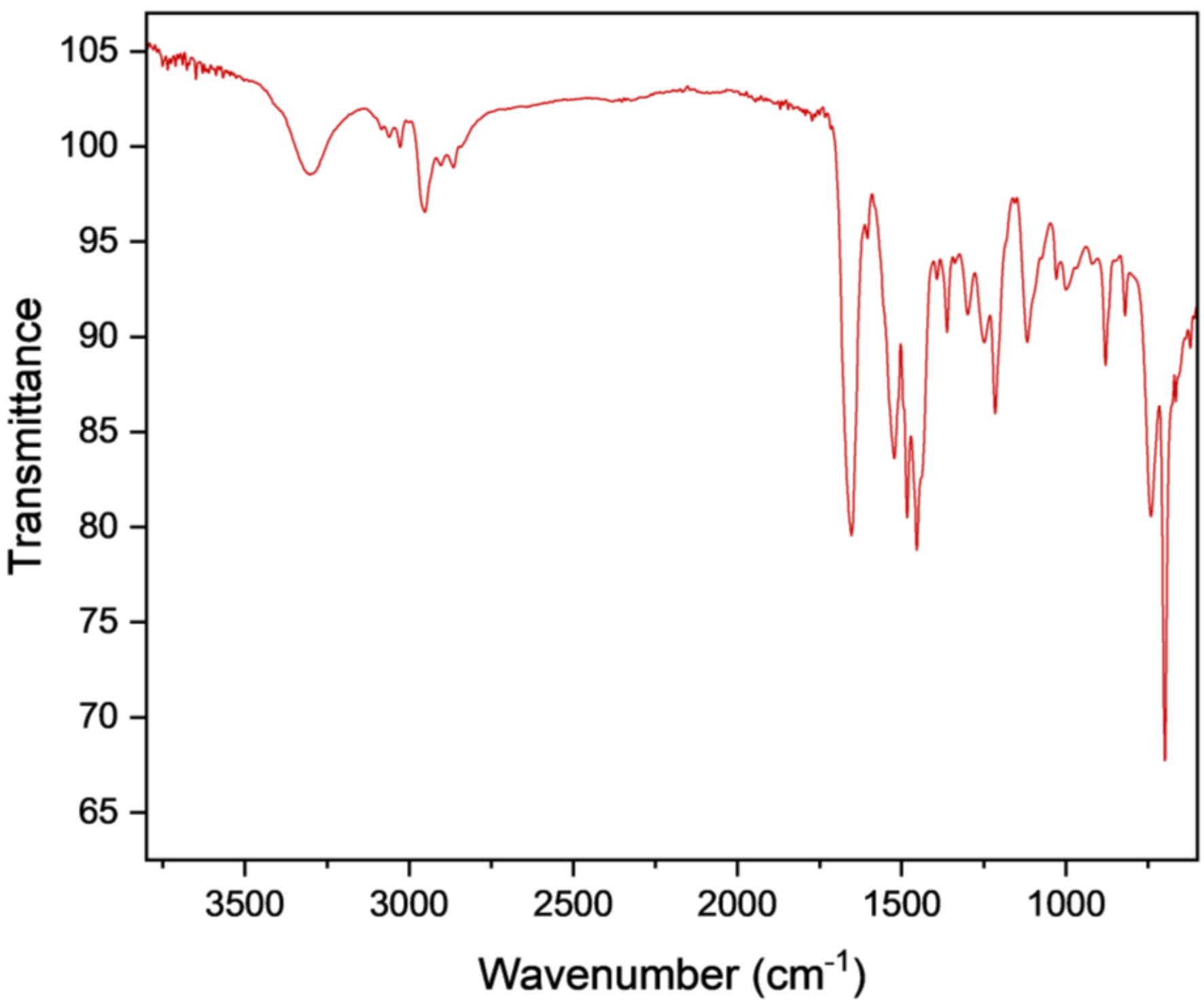


**Figure S18**. FTIR (ATR) for **L1**.

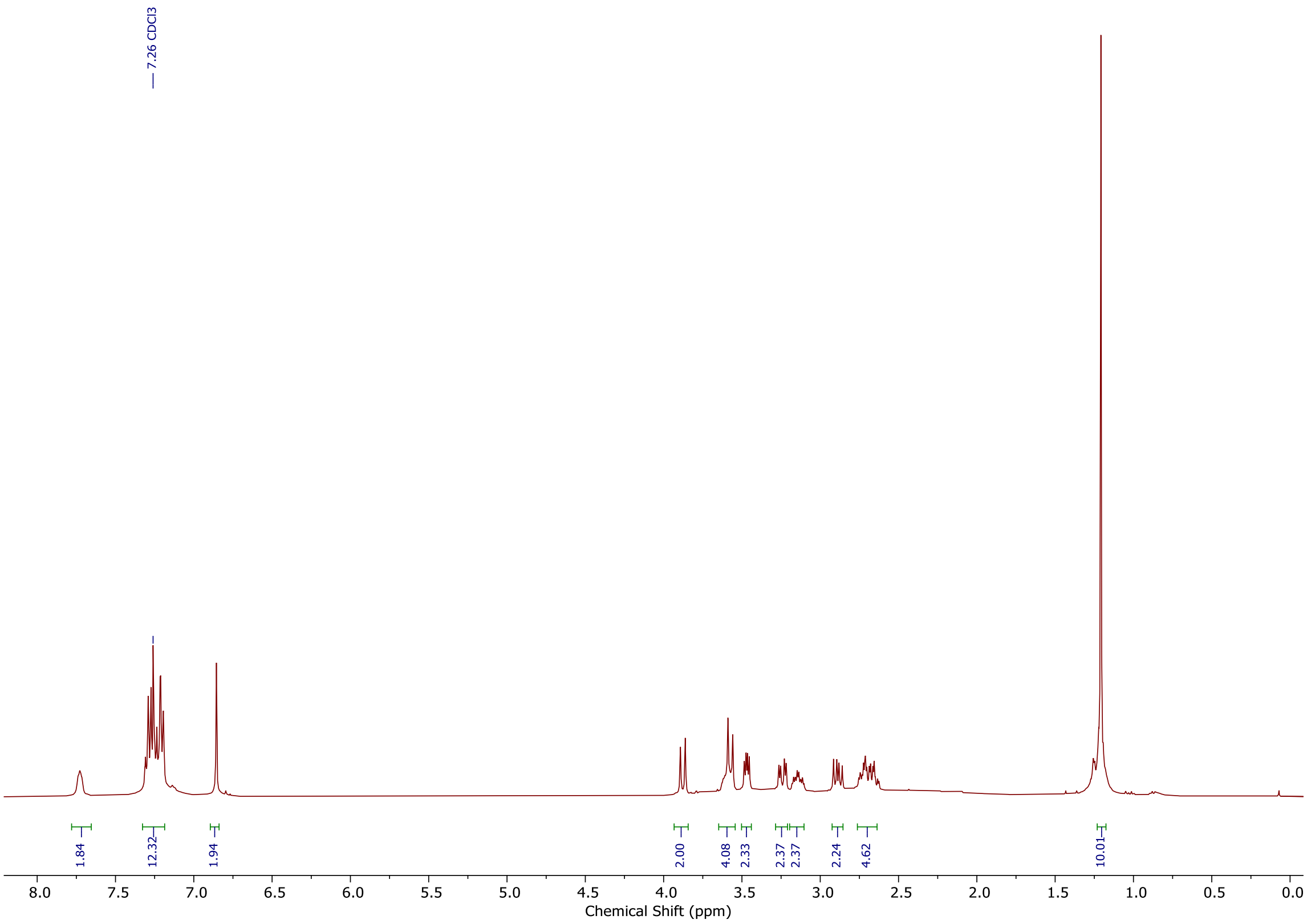


**Figure S19**. $^{1}$H NMR (400 MHz, $CDCl_3$, 25ºC) for **L2A** (5 mM).

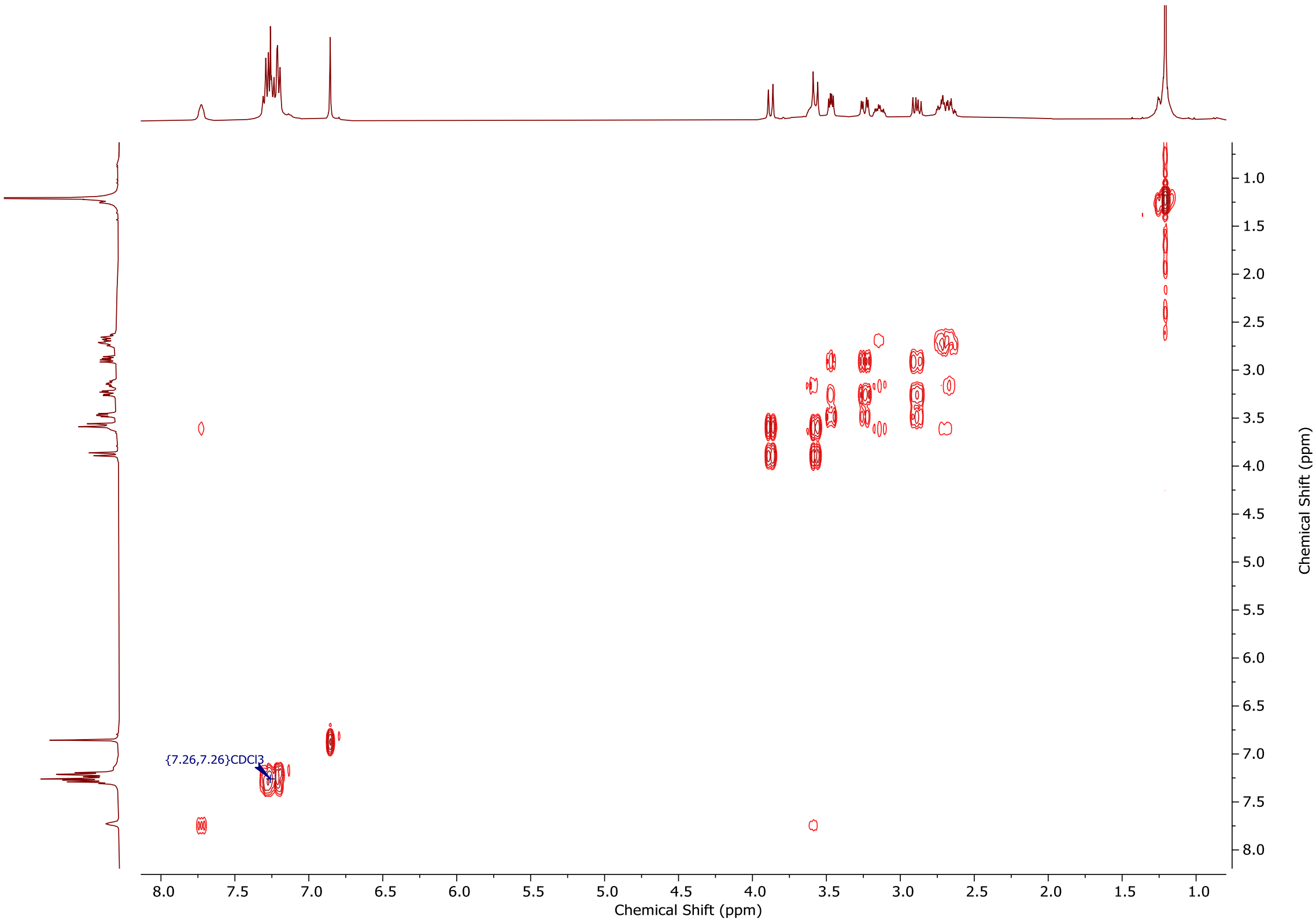


**Figure S20**. 2D COSY NMR (400 MHz, $CDCl_3$, 25ºC) for **L2A** (5 mM).

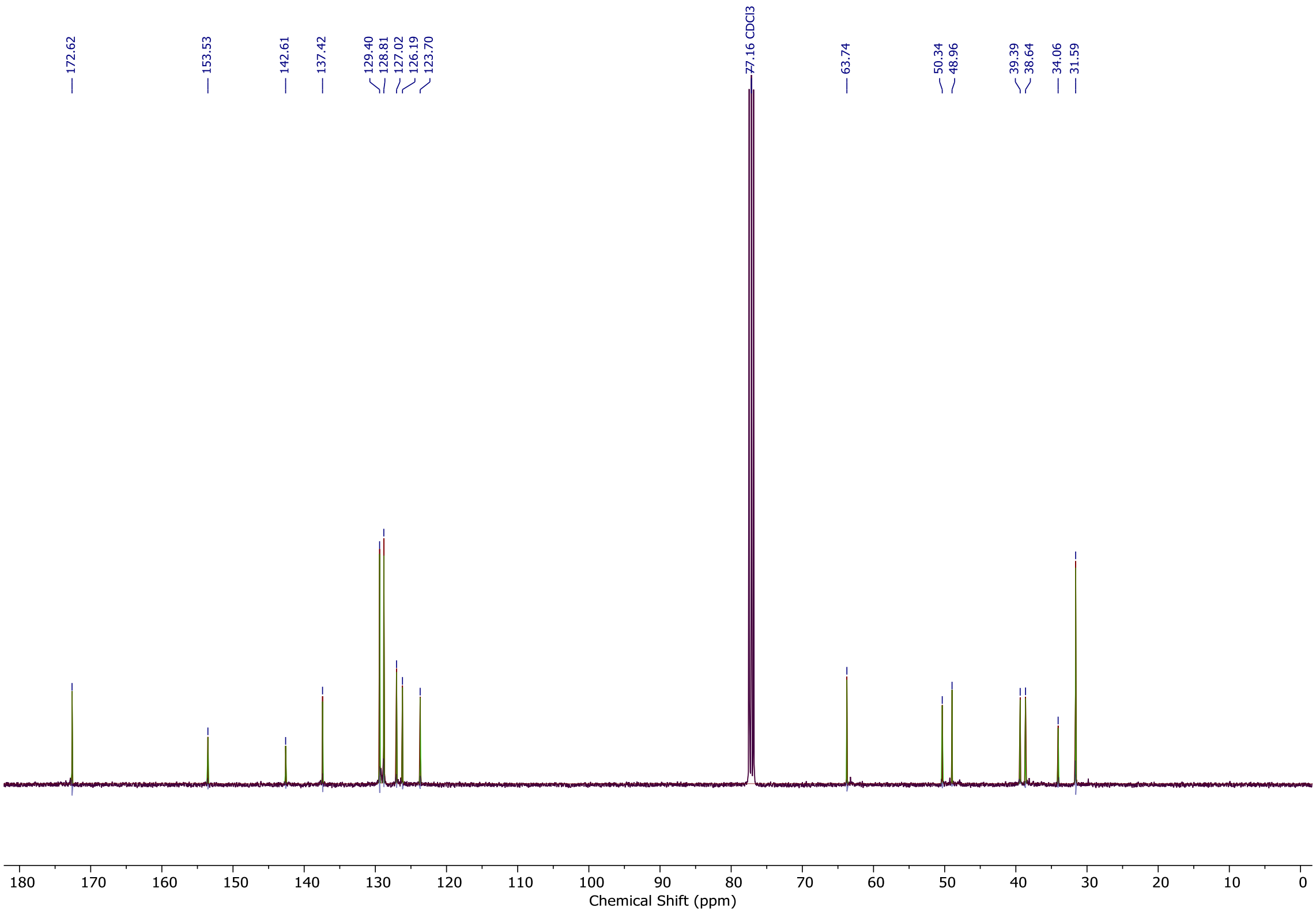


**Figure S21**. $^{13}$CNMR (400 MHz, $CDCl_3$, 25ºC) for **L2A** (5 mM).

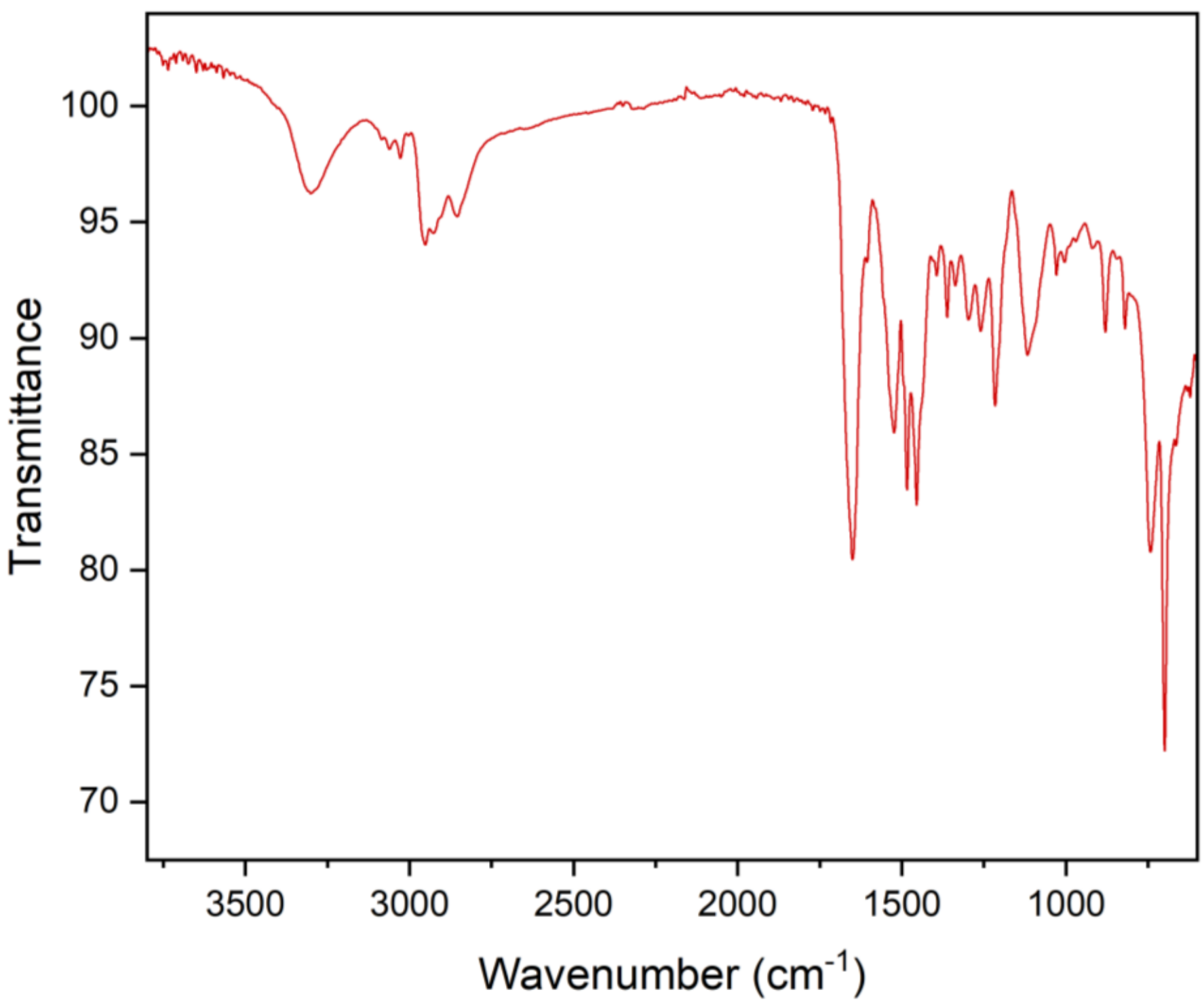


**Figure S22**. FTIR (ATR) for **L2A**.

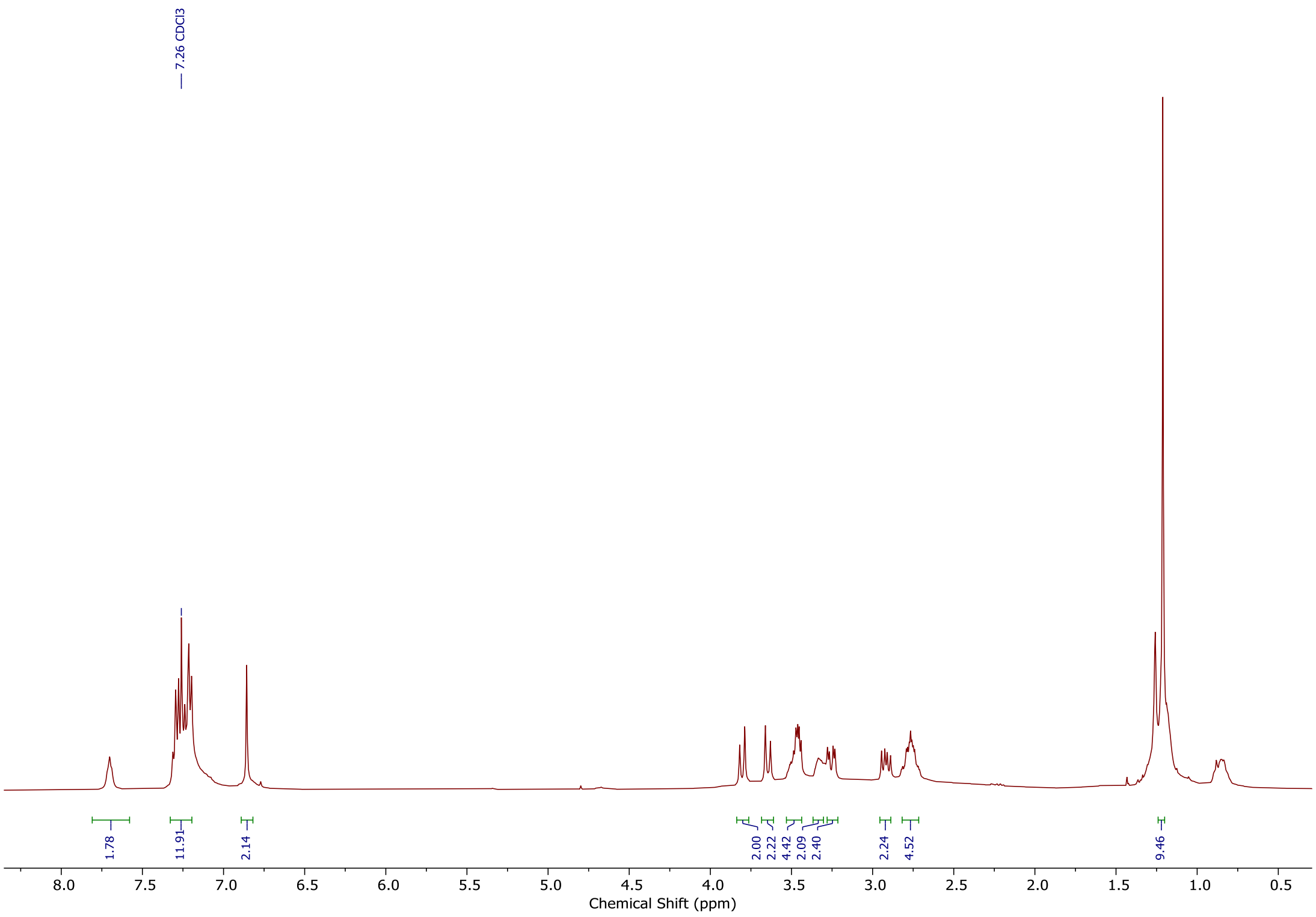


**Figure S23**. $^{1}$H NMR (400 MHz, $CD_3OD$, 25ºC) for **L2B** (5 mM).

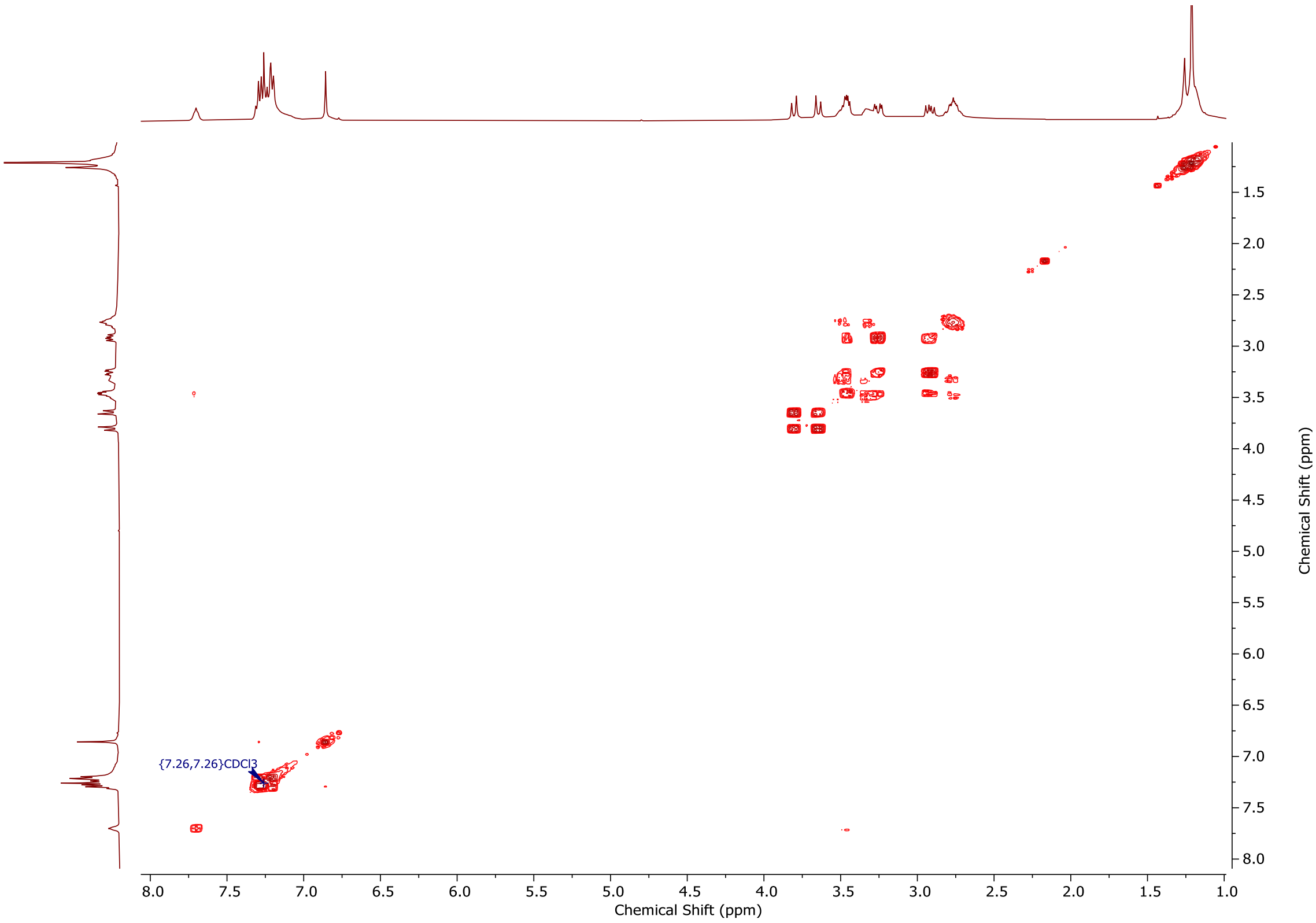


**Figure S24**. 2D COSY NMR (400 MHz, $CDCl_3$, 25ºC) for **L2B** (5 mM).

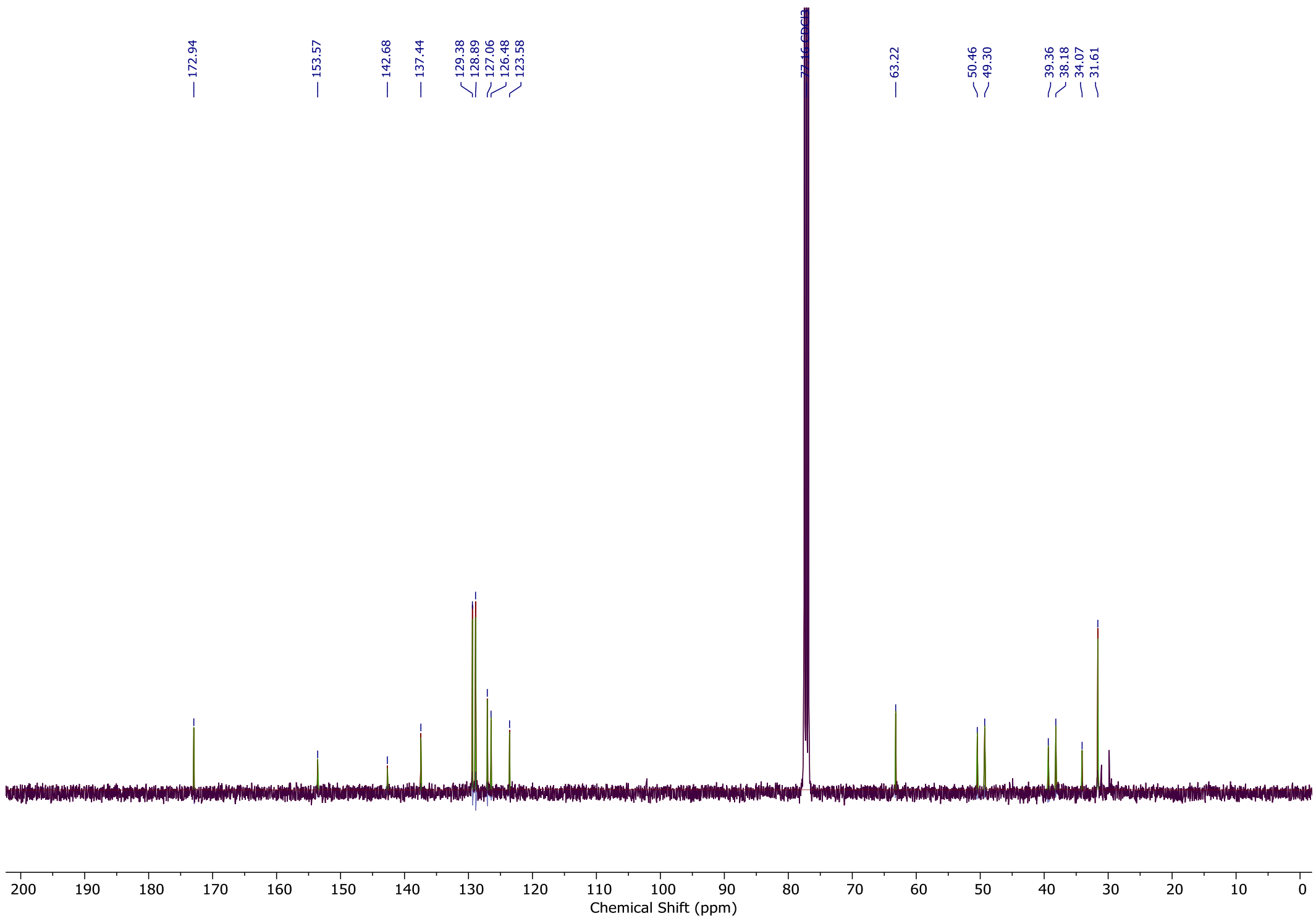


**Figure S25**. $^{13}C$ NMR (400 MHz, $CDCl_3$, 25ºC) for **L2B** (5 mM).

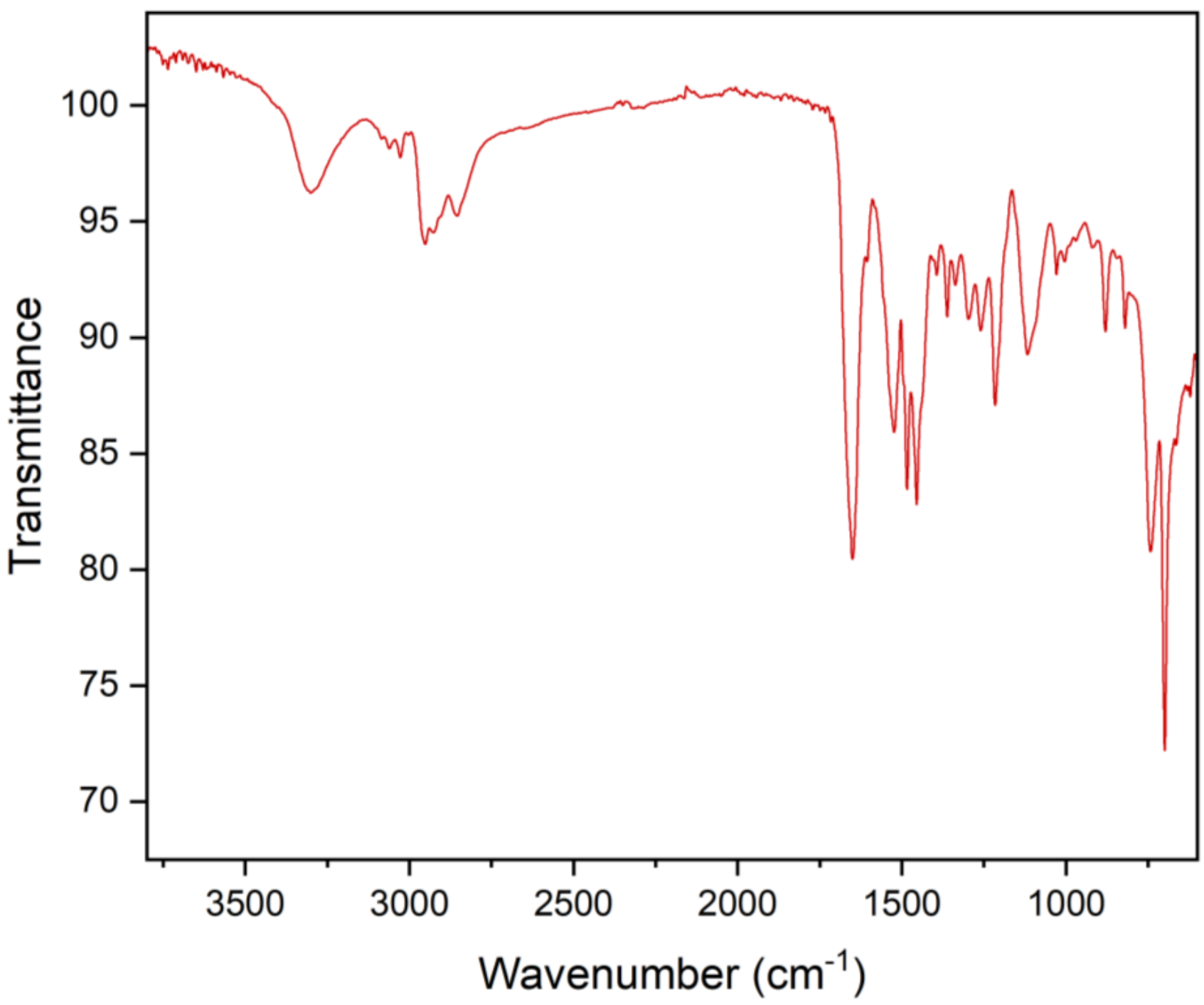


**Figure S26**. FTIR (ATR) for **L2B**.

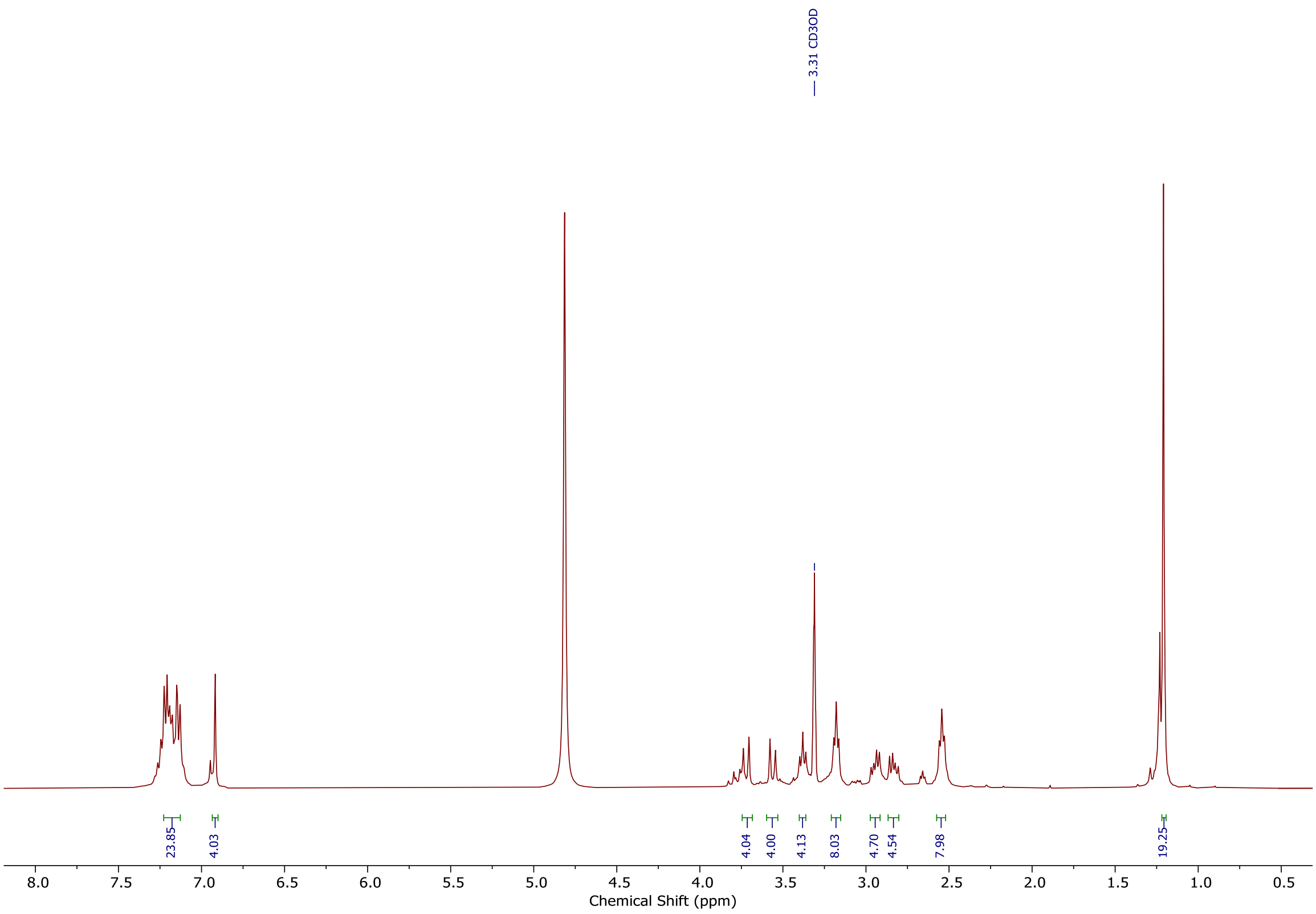


**Figure S27**. $^{1}$H NMR (400 MHz, $CD_3OD$, 25ºC) for **L3** (5 mM).

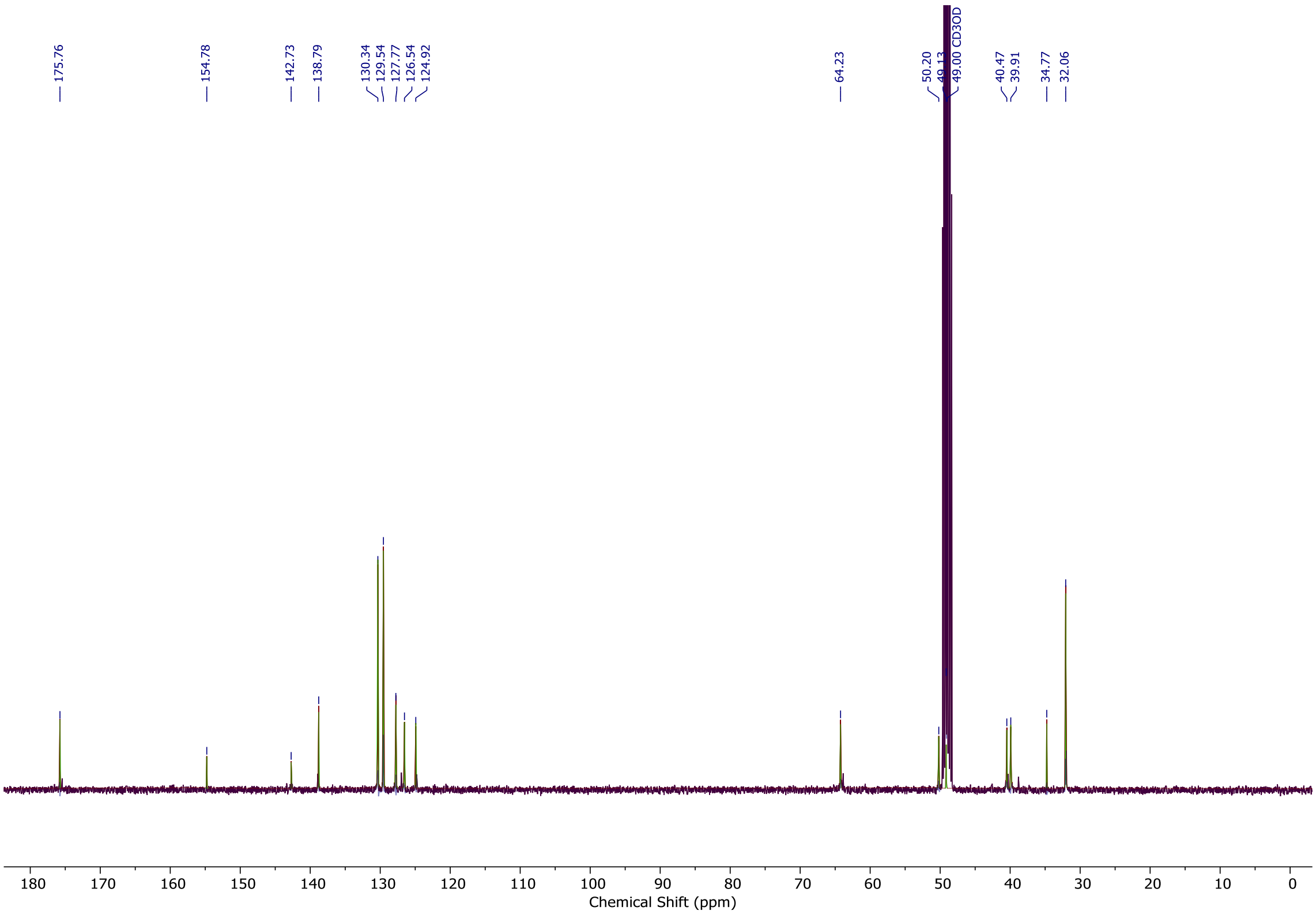


**Figure S28**.$^{13}$C NMR (400 MHz, $CD_3OD$, 25ºC) for **L3** (5 mM).

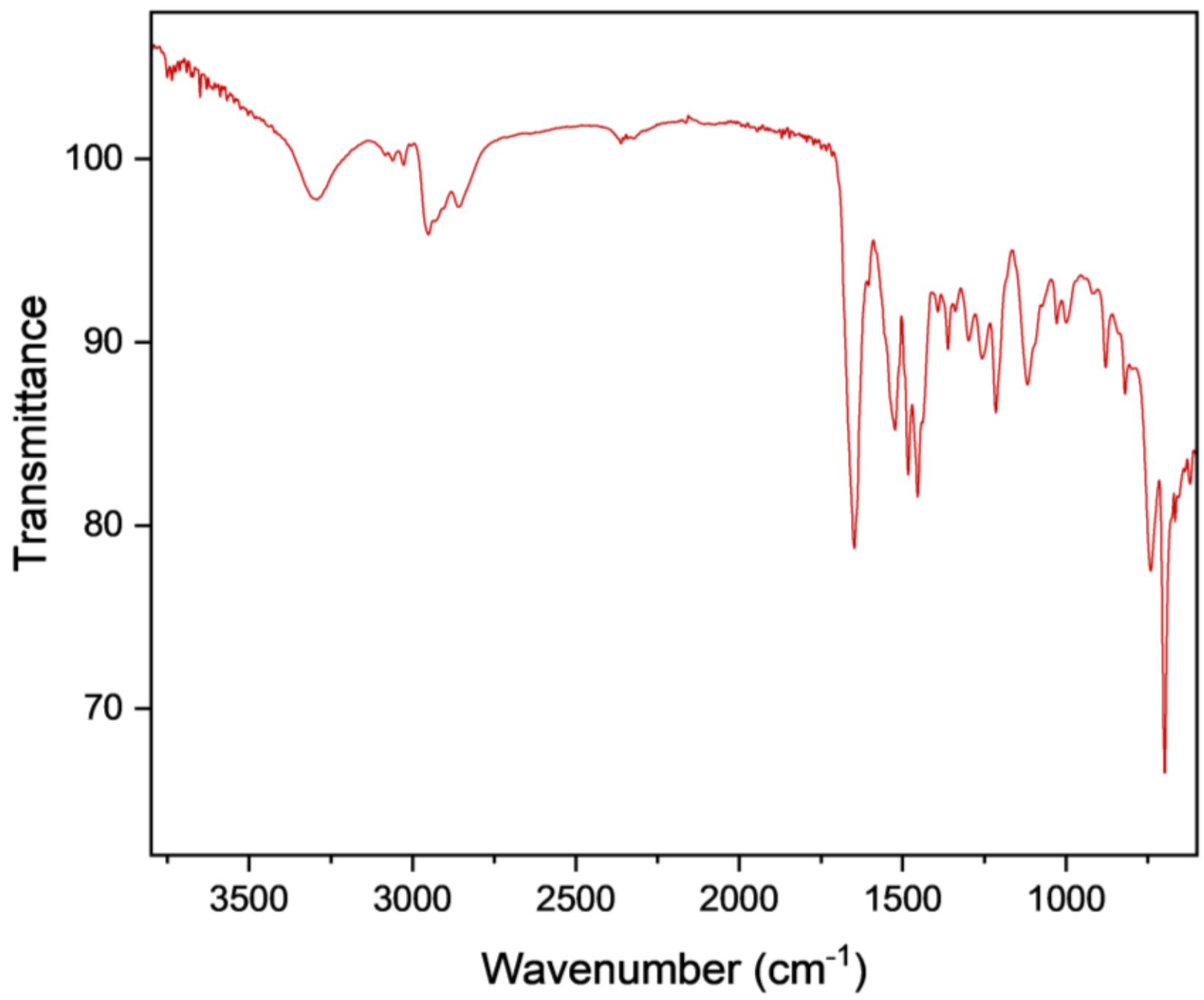


**Figure S29**. FTIR (ATR) for **L3**.

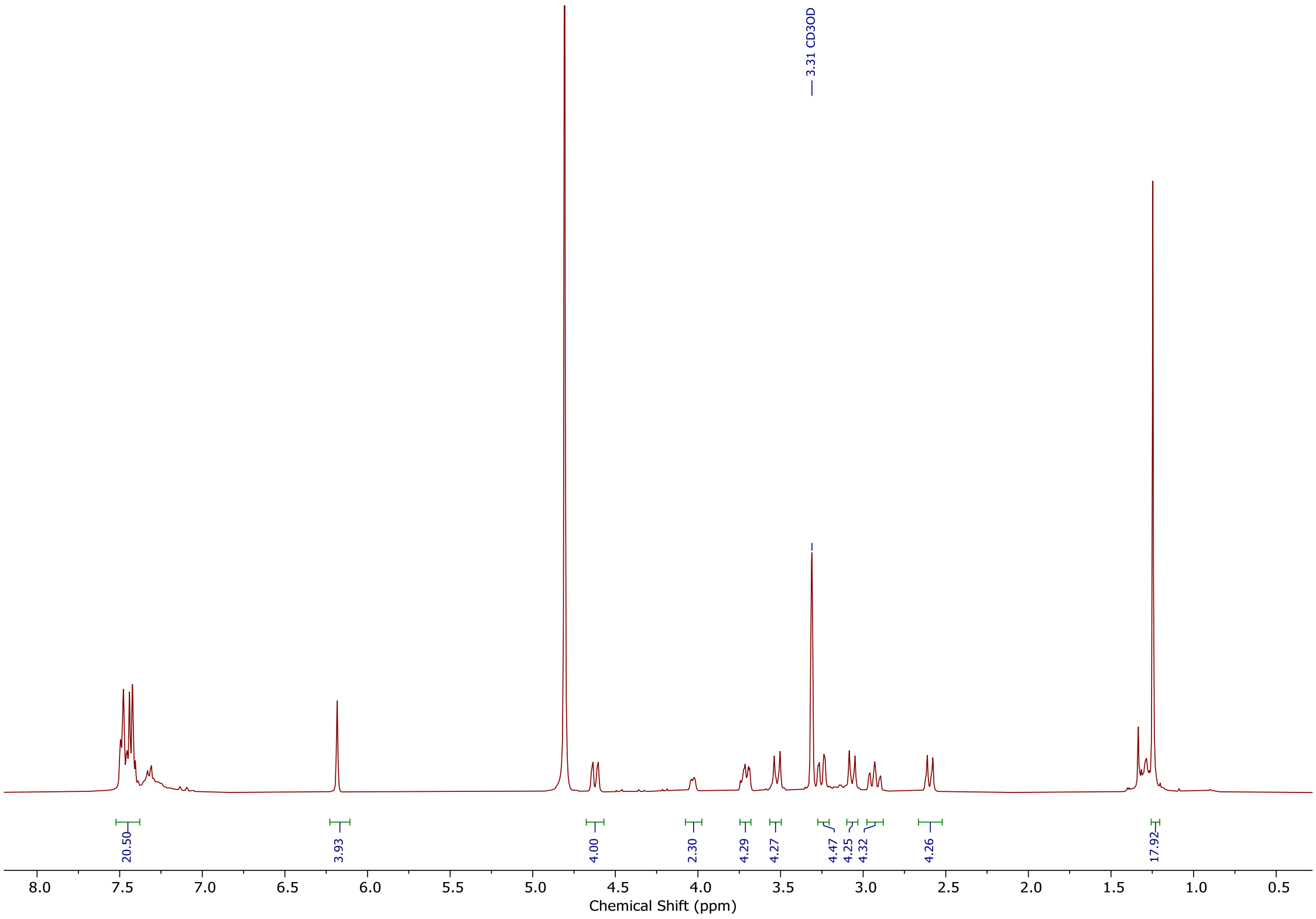


**Figure S30**. $^{1}$H NMR (400 MHz, $CD_3OD$, 25ºC) for **C1** (5 mM).

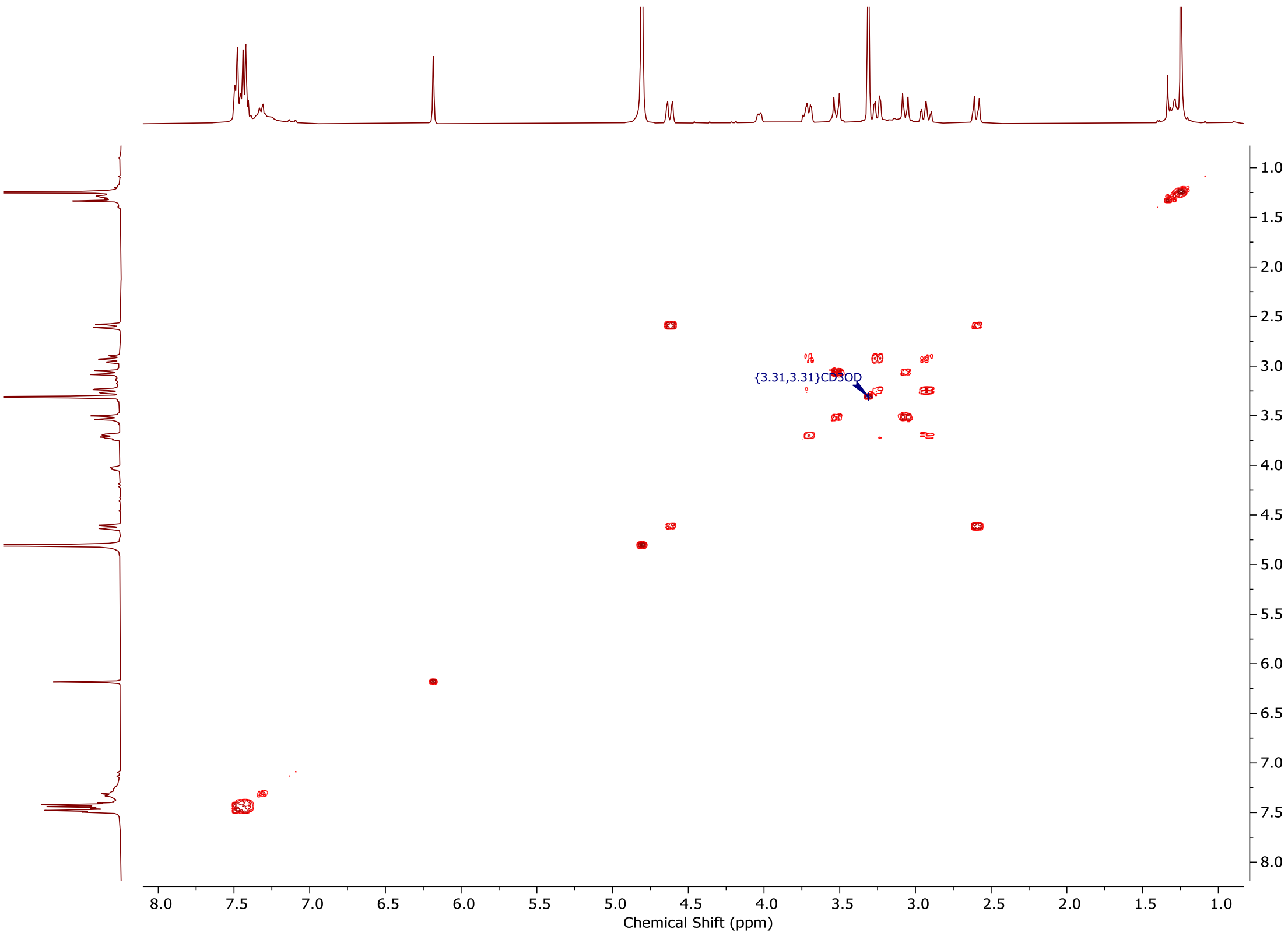


**Figure S31**. 2D COSY NMR (400 MHz, $CD_3OD$, 25ºC) for **C1** (5 mM).

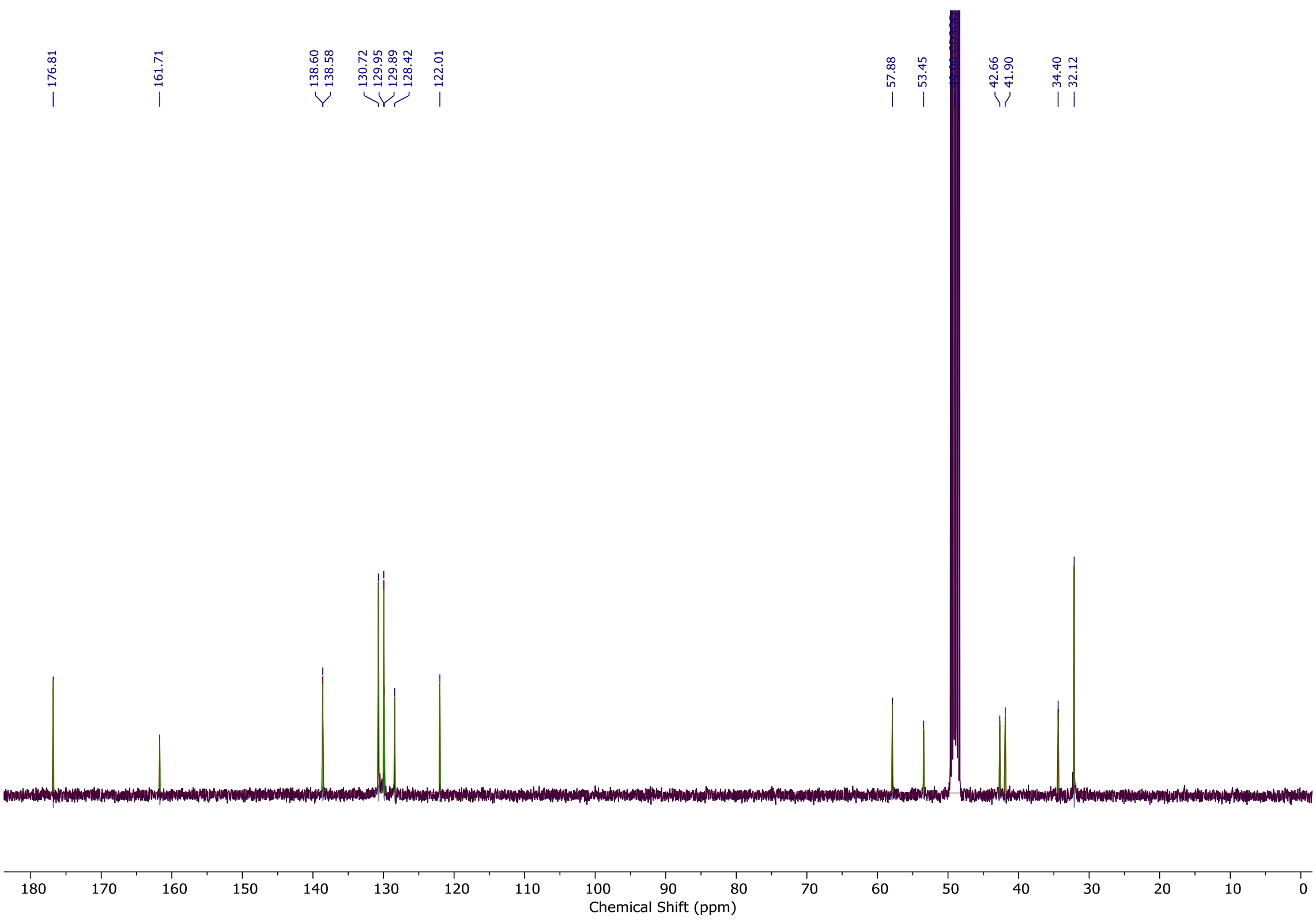


**Figure S32**. $^{13}$C NMR (400 MHz, $CD_3OD$, 25ºC) for **C1** (5 mM).

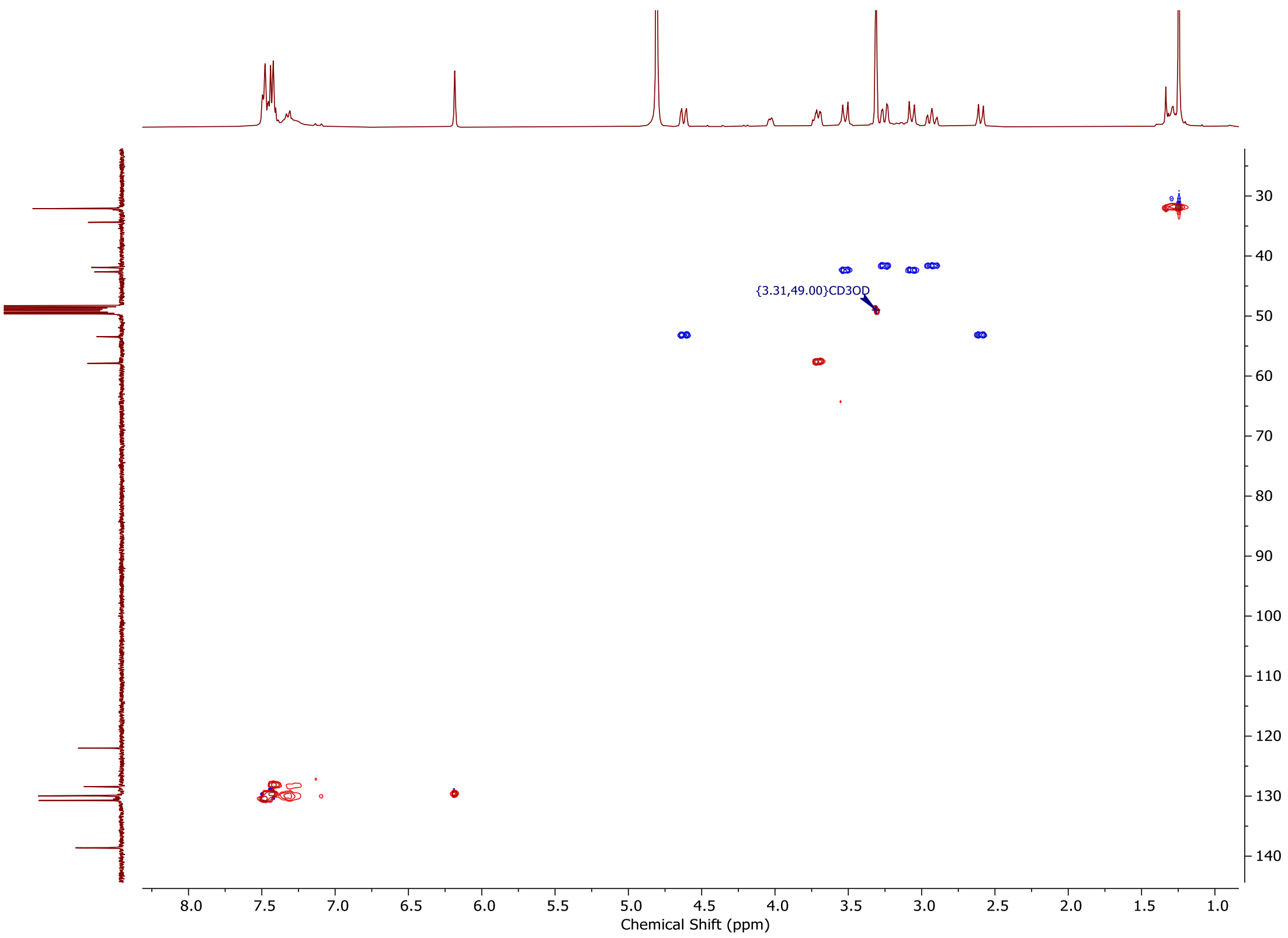


**Figure S33**. 2D HSQC NMR (400 MHz, $CD_3OD$, 25ºC) for **C1** (5 mM).

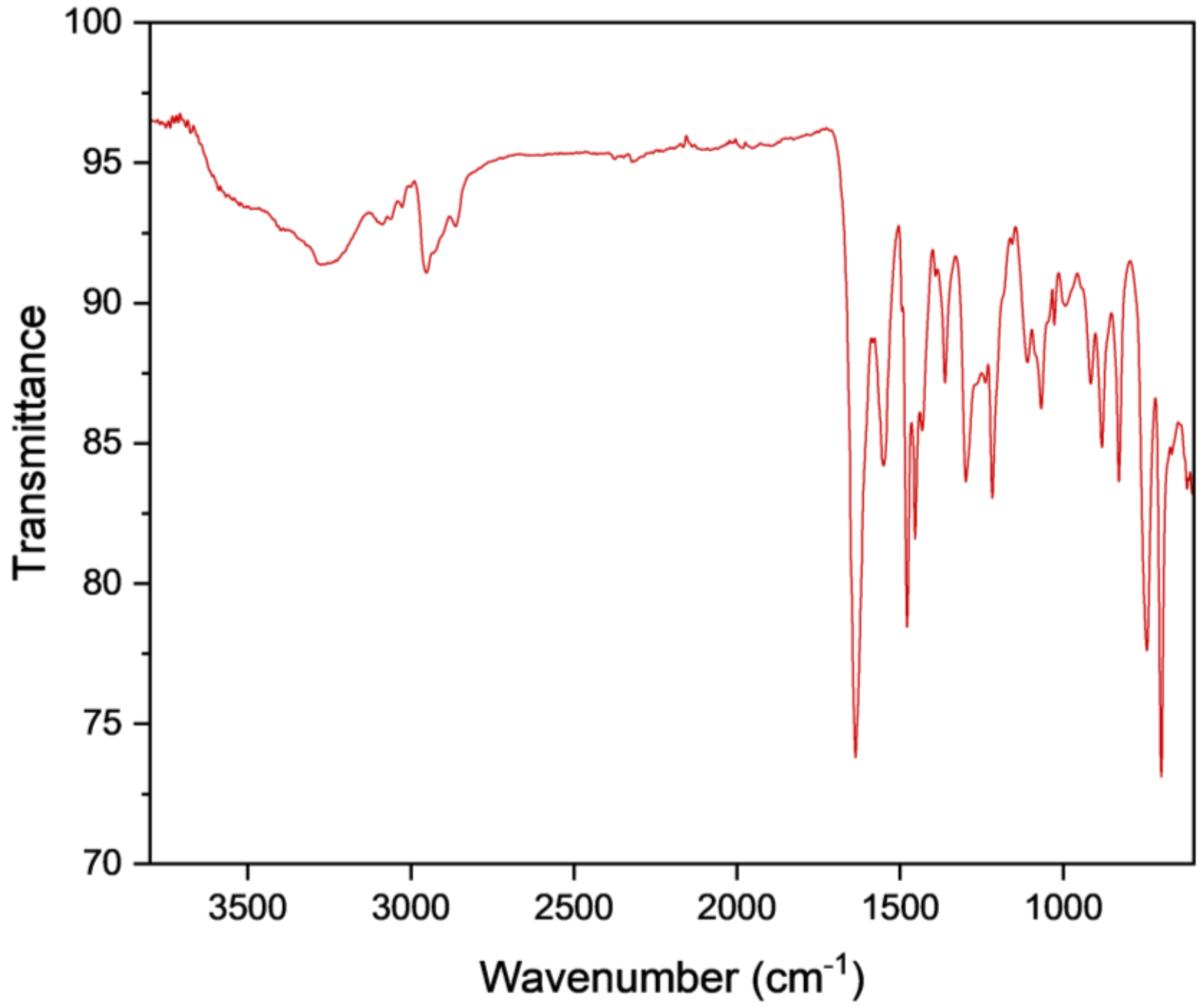


**Figure S34**. FTIR (ATR) for **C1**.

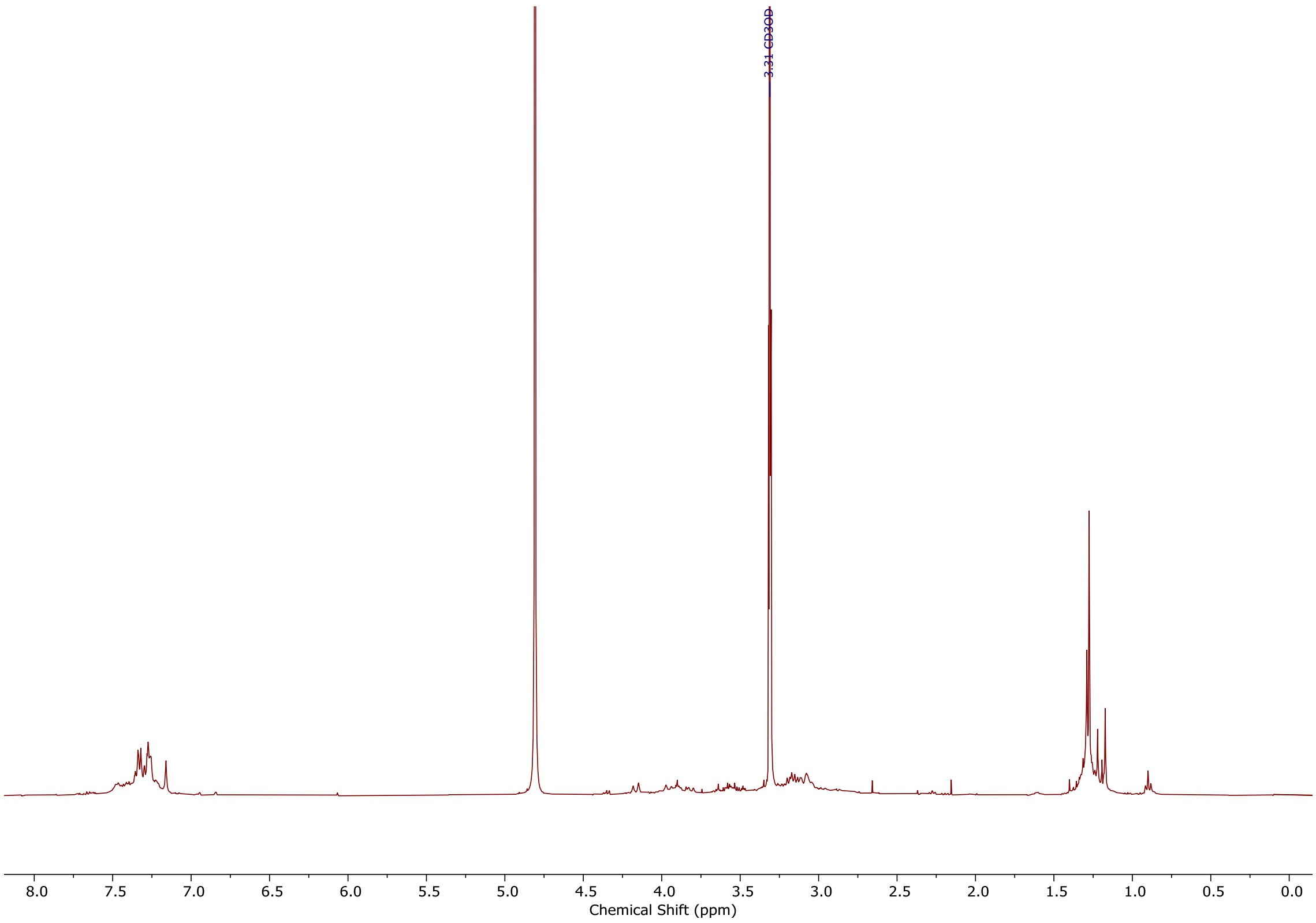


**Figure S35**. $^1$H NMR (400 MHz, $CD_3OD$, 25ºC) for **C2** (5 mM).

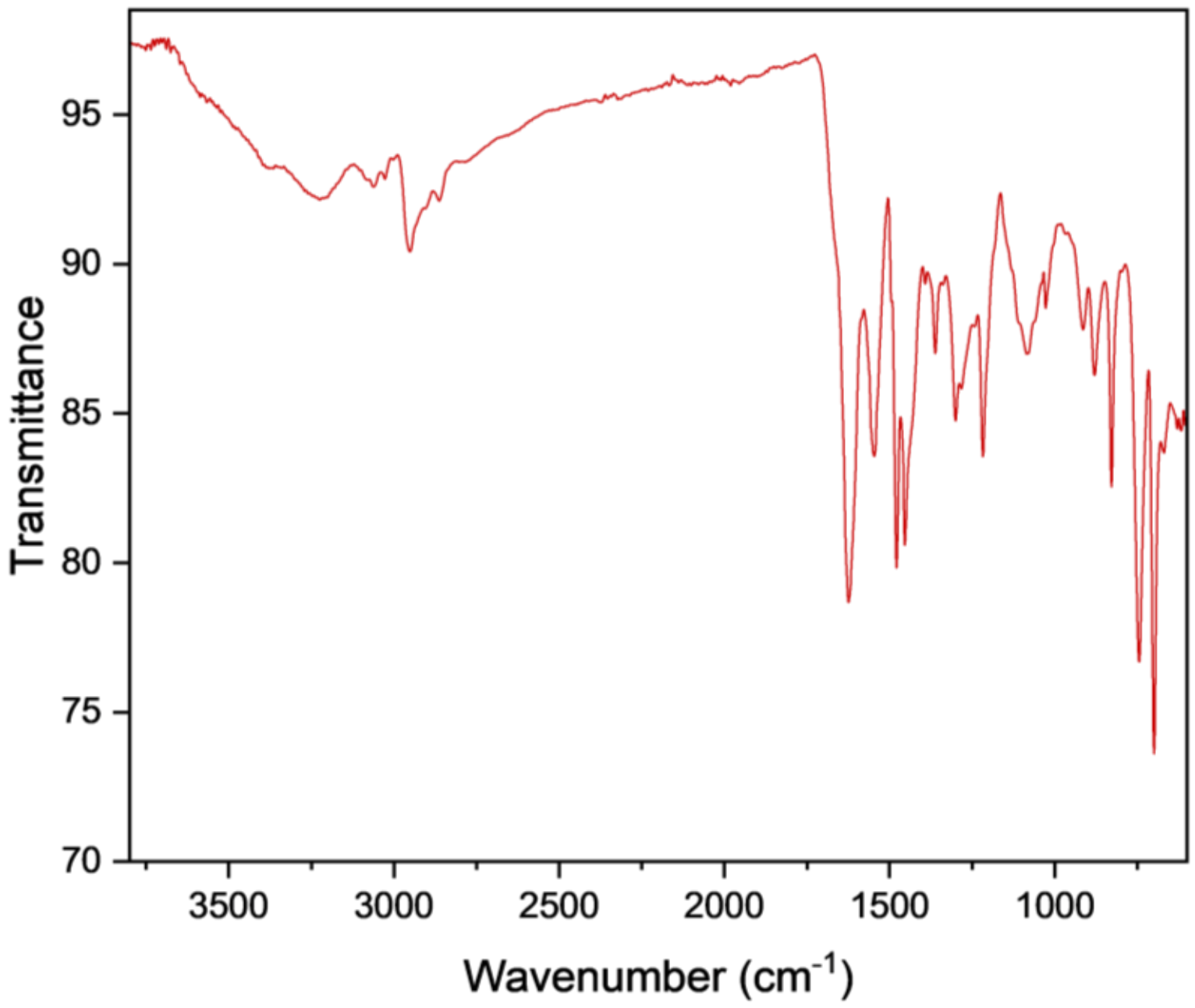


**Figure S36**. FTIR (ATR) for **C2**.

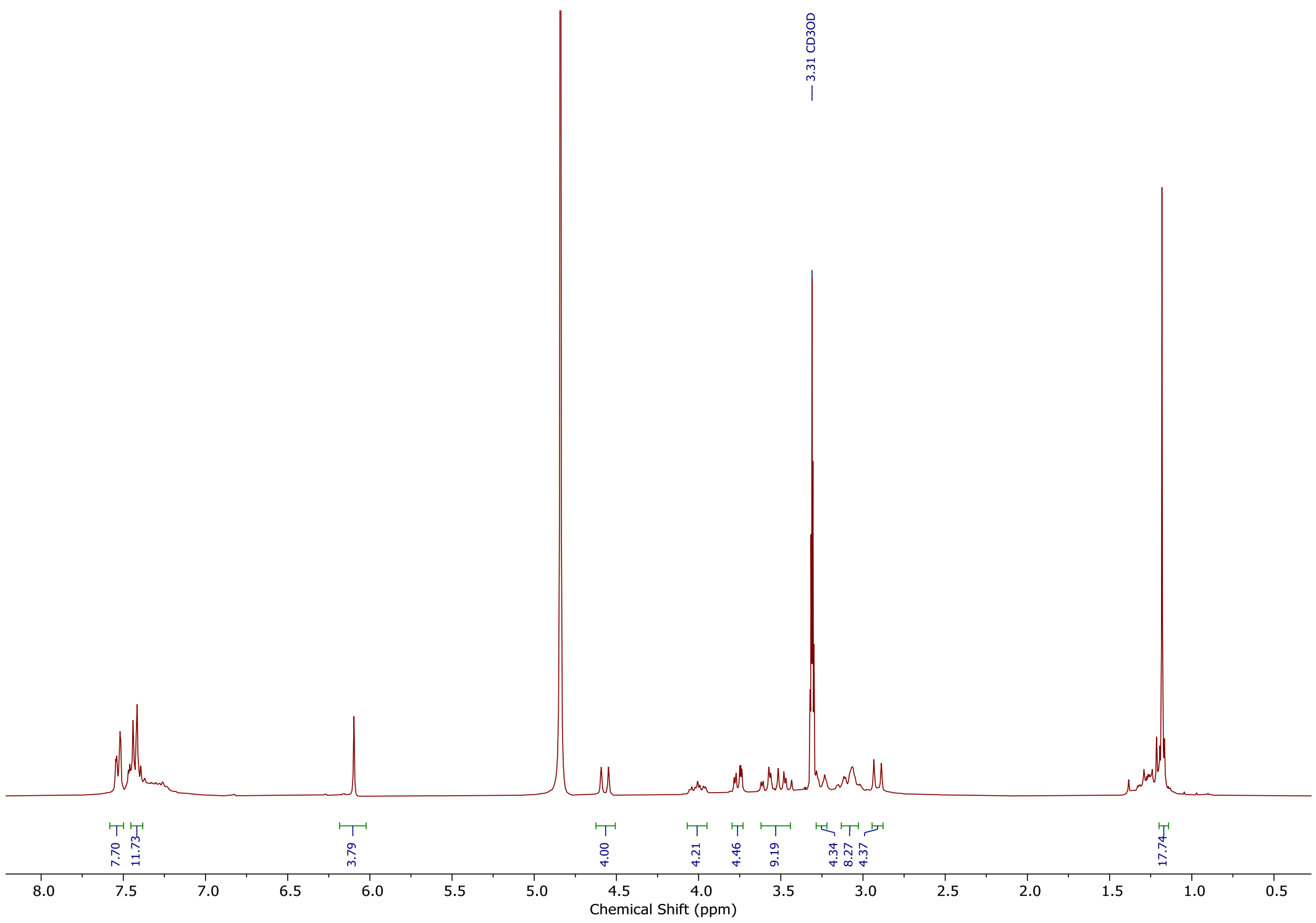


**Figure S37**. $^{1}$H NMR (400 MHz, $CD_3OD$, 25ºC) for **C3** (5 mM).

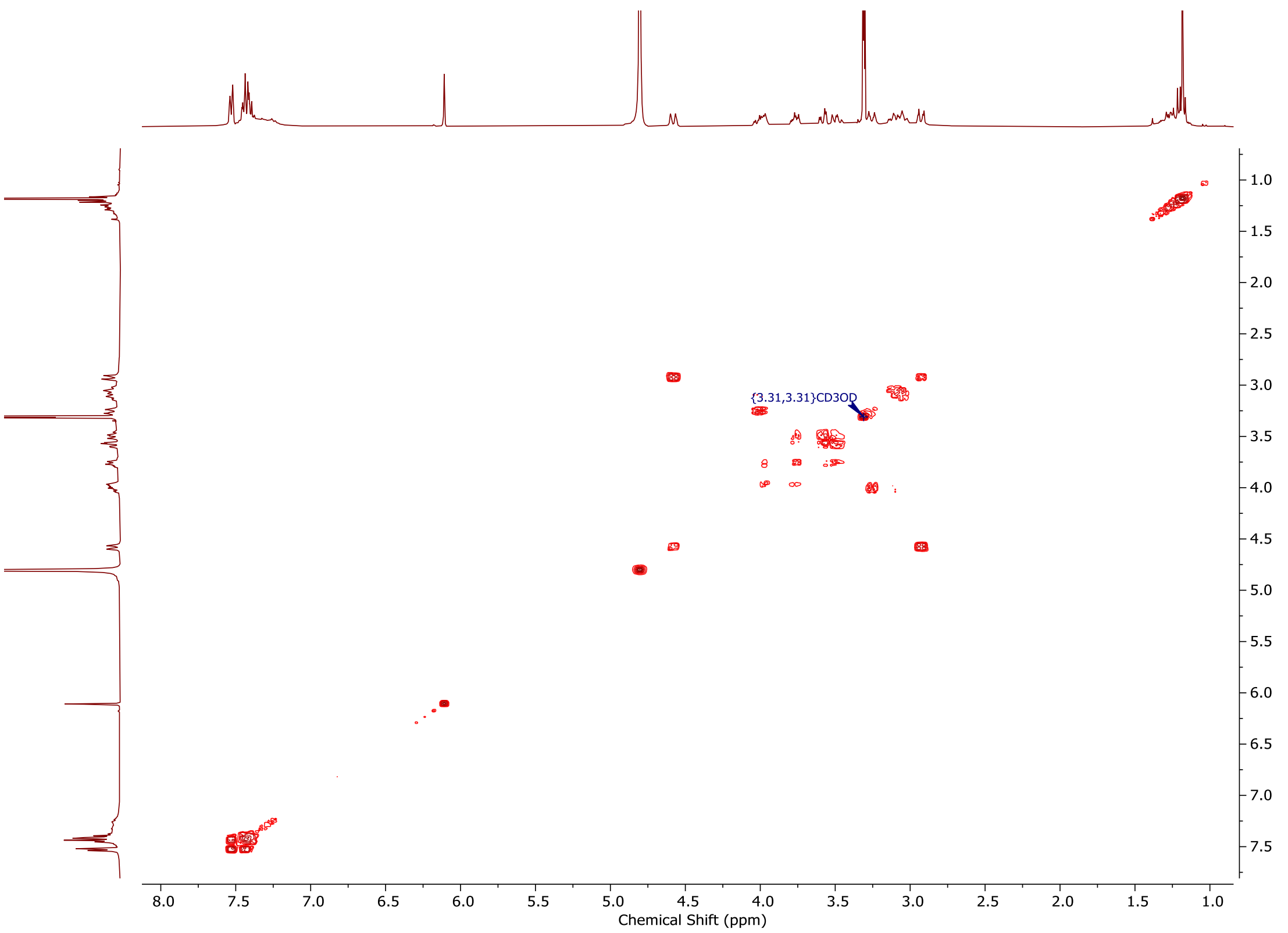


**Figure S38**. 2D COSY NMR (400 MHz, $CD_3OD$, 25ºC) for **C3** (5 mM).

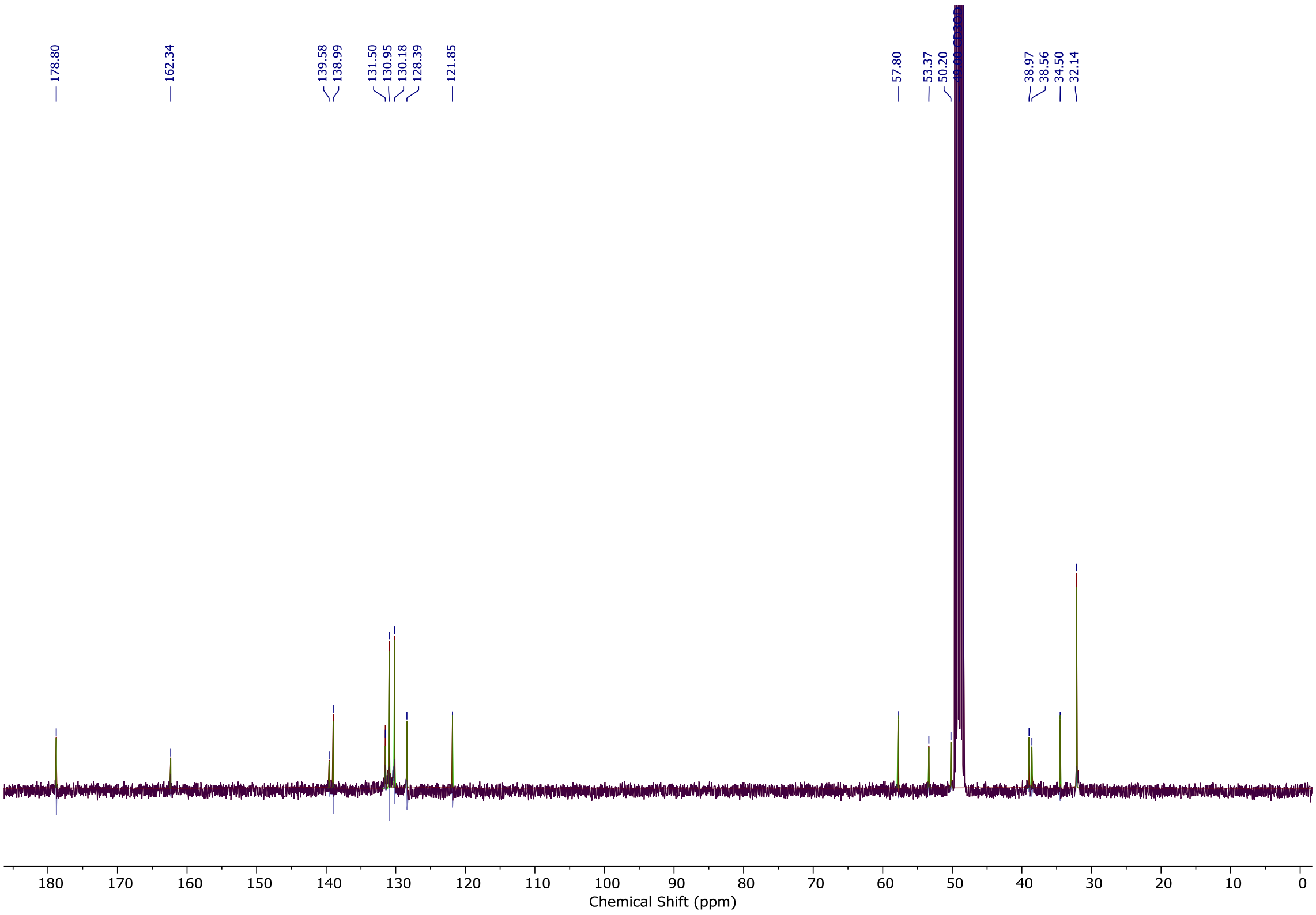


**Figure S39**. $^{13}C$ NMR (400 MHz, $CD_3OD$, 25ºC) for **C3** (5 mM).

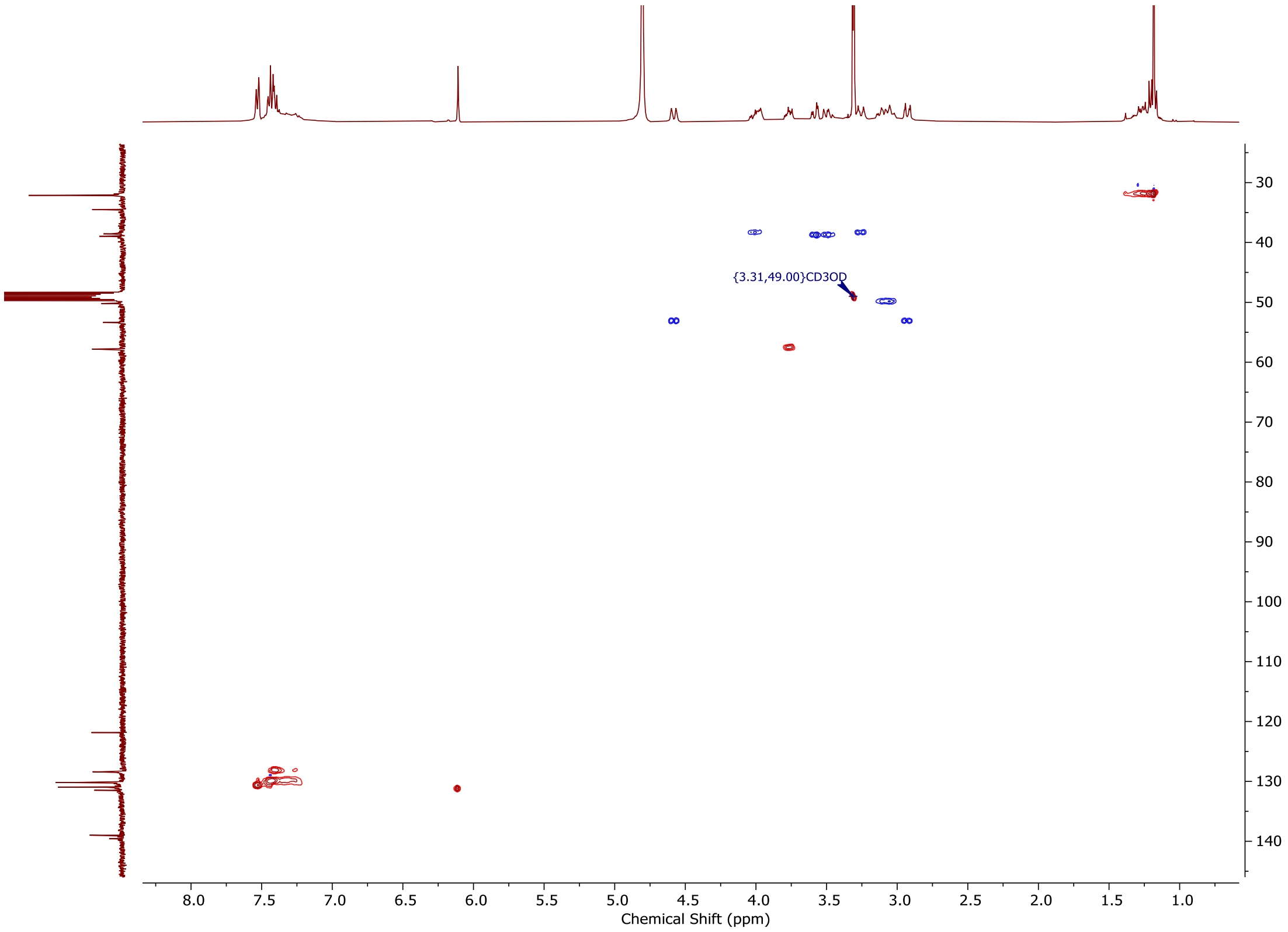


**Figure S40**. 2D HSQC NMR (400 MHz, $CD_3OD$, 25ºC) for **C3** (5 mM).

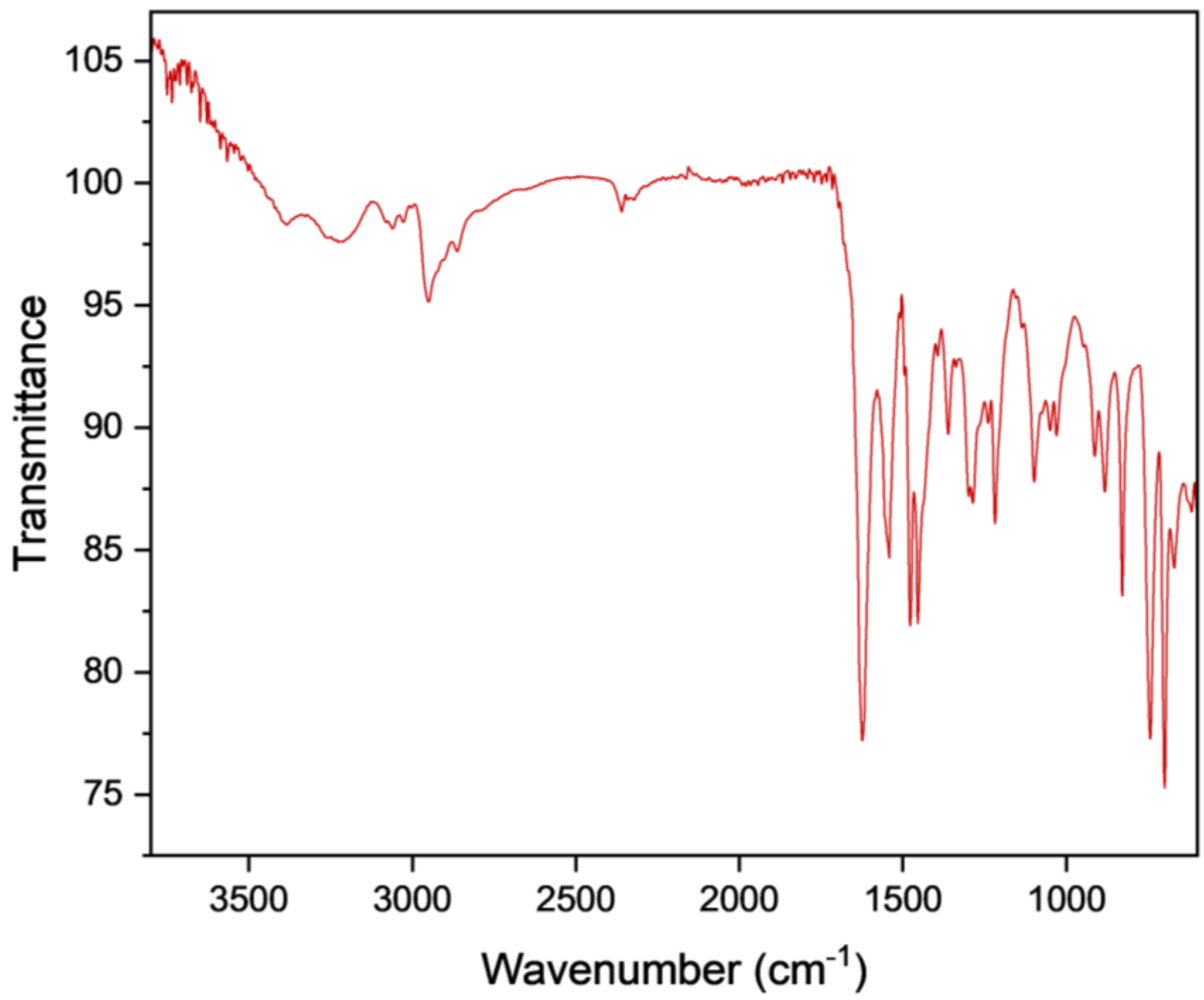


**Figure S41**. FTIR (ATR) for **C3**.

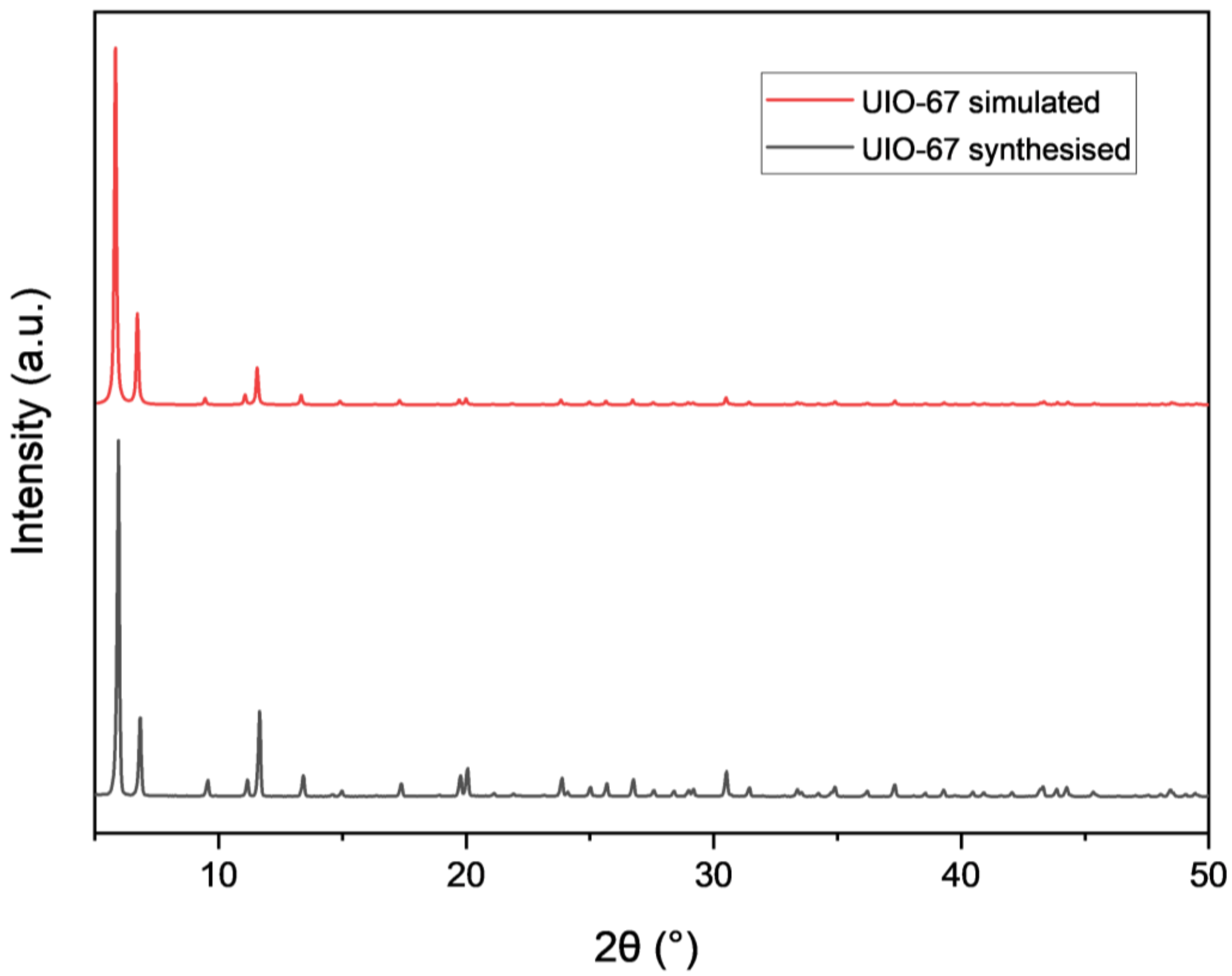


**Figure S42**. XRD pattern of **UiO-67** vs simulated XRD pattern.

## 6. CRYSTAL STRUCTURE OF C3

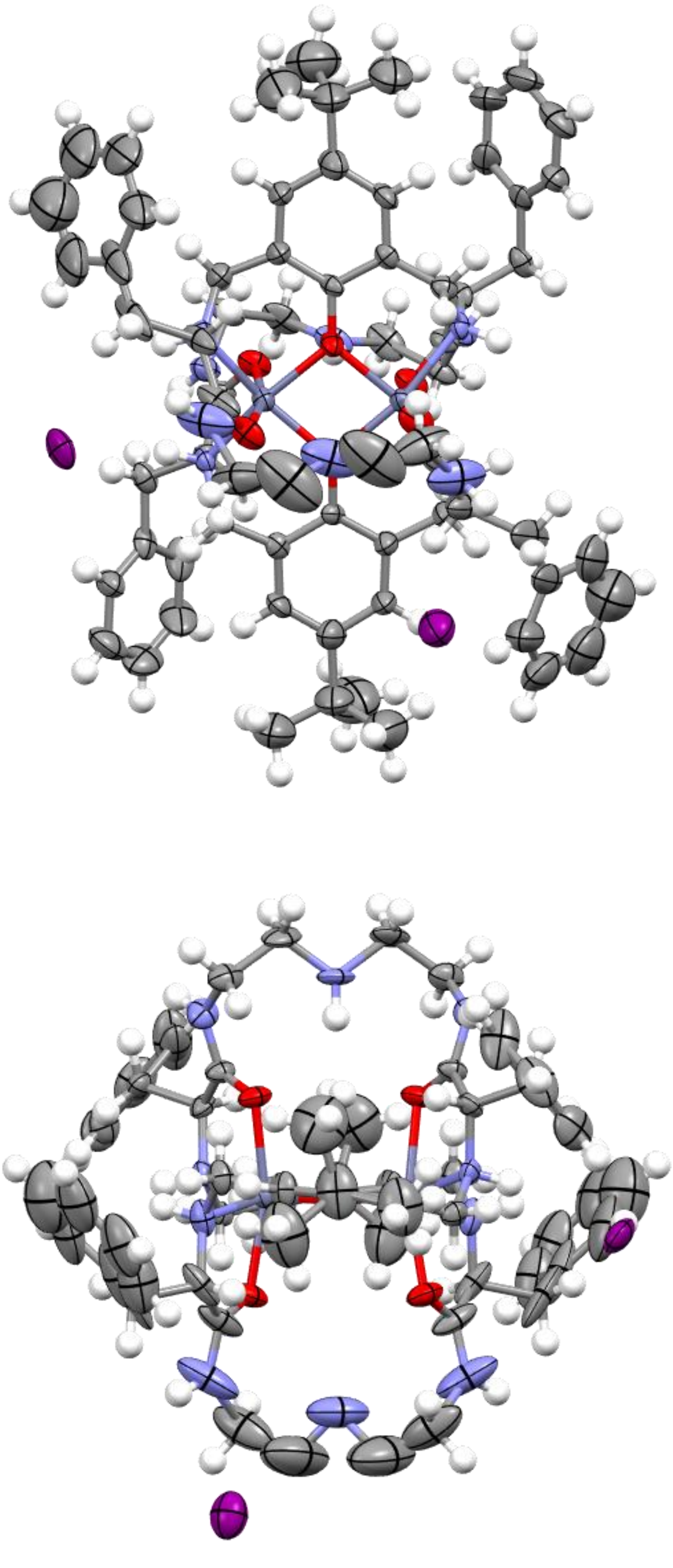

**Figure S43.** Thermal ellipsoid plot for the crystal structure of **C3**. Ellipsoids at 50% of probability.

**Table S3**. Crystallographic and structural refinement data for compound **C3**.

| Empirical Formula | C38.5H58.5Cl3I2N5O7.5Zn |
|---|---|
| Formula weight | 1136.985 |
| Temperature/K | 200.15 |
| Crystal system | orthorhombic |
| Space group | $C222_1$ |
| a/Å | 14.7053(2) |
| b/Å | 22.6803(3) |
| c/Å | 29.6309(4) |
| α/° | 90 |
| β/° | 90 |
| γ/° | 90 |
| Volume/Å$^3$ | 9882.5(2) |
| Z | 8 |
| ρ_calc / g·cm$^{-3}$ | 1.528 |
| F(000) | 4583.4 |
| Crystal size / mm$^3$ | 0.263 × 0.246 × 0.148 |
| Radiation | Cu Kα (λ = 1.54184) |
| 2Θ range for data collection / ° | 7.16 to 143.66 |
| Index ranges | -11 ≤ h ≤ 18, -27 ≤ k ≤ 25, -36 ≤ l ≤ 32 |
| Reflections collected | 30751 |
| Independent reflections | 9467 [Rint = 0.0734, Rsigma = 0.0443] |
| Data/restraints/parameters | 9467/110/410 |
| Goodness-of-fit on F$^2$ | 1.024 |
| Final R indexes [I ≥ 2σ(I)] | R1 = 0.0697, wR2 = 0.1955 |
| Final R indexes [all data] | R1 = 0.0725, wR2 = 0.1992 |
| Largest diff. peak/hole / e Å$^{-3}$ | 1.19 / -0.76 |
| Flack parameter | 0.048(3) |

## REFERENCES


[1] Z. Yang, F. Esteve, C. Antheaume, J.-M. Lehn, "Dynamic covalent self-assembly and self-sorting processes in the formation of imine-based macrocycles and macrobicyclic cages" Chemical Science **14**, (2023): 6631.

[2] F. Esteve, R. Porcar, M. Bolte, B. Altava, S. V. Luis, E. García-Verdugo, "A bioinspired approach toward efficient supramolecular catalysts for $CO_2$ conversion" Chem Catalysis **3**, (2023): 100482.

[3] P. Pracht, F. Bohle, S. Grimme, "Automated exploration of the low-energy chemical space with fast quantum chemical methods" Physical Chemistry Chemical Physics **22**, (2020): 7169-7192.

[4] C. Bannwarth, S. Ehlert, S. Grimme, "GFN2-xTB—An Accurate and Broadly Parametrized Self-Consistent Tight-Binding Quantum Chemical Method with Multipole Electrostatics and Density-Dependent Dispersion Contributions" Journal of Chemical Theory and Computation **15**, (2019): 1652-1671.
